# ALS/FTD-Linked Mutation in FUS Suppresses Intra-axonal Protein Synthesis and Drives Disease Without Nuclear Loss-of-Function of FUS

**DOI:** 10.1016/j.neuron.2018.09.044

**Published:** 2018-11-21

**Authors:** Jone López-Erauskin, Takahiro Tadokoro, Michael W. Baughn, Brian Myers, Melissa McAlonis-Downes, Carlos Chillon-Marinas, Joshua N. Asiaban, Jonathan Artates, Anh T. Bui, Anne P. Vetto, Sandra K. Lee, Ai Vy Le, Ying Sun, Mélanie Jambeau, Jihane Boubaker, Deborah Swing, Jinsong Qiu, Geoffrey G. Hicks, Zhengyu Ouyang, Xiang-Dong Fu, Lino Tessarollo, Shuo-Chien Ling, Philippe A. Parone, Christopher E. Shaw, Martin Marsala, Clotilde Lagier-Tourenne, Don W. Cleveland, Sandrine Da Cruz

**Affiliations:** 1 Ludwig Institute for Cancer Research, University of California at San Diego, La Jolla, CA 92093, USA; 2 Department of Cellular and Molecular Medicine, University of California at San Diego, La Jolla, CA 92093, USA; 3 Department of Neurosciences, University of California at San Diego, La Jolla, CA 92093, USA; 4 Department of Anesthesiology, University of California at San Diego, La Jolla, CA 92093, USA; 5 Mouse Cancer Genetics Program, National Cancer Institute, Frederick, MD, 21702, USA; 6 Regenerative Medicine Program and Department of Biochemistry & Medical Genetics, University of Manitoba, Winnipeg, R3T 2N2, Canada; 7 United Kingdom Dementia Research Institute Centre, Maurice Wohl Clinical Neuroscience Institute, Institute of Psychiatry, Psychology and Neuroscience, King’s College London, SE5 9NU London, U.K; 8 Centre for Brain Research, University of Auckland, Auckland, New Zealand

**Keywords:** Amyotrophic Lateral Sclerosis, ALS, Frontotemporal dementia, FTD, FUS, TLS, neurodegenerative disease, axonal translation, RNA-binding proteins, protein synthesis

## Abstract

Through the generation of humanized FUS mice expressing full-length human FUS, we identify that when expressed at near endogenous murine FUS levels, both wild-type and ALS-causing and frontotemporal dementia (FTD)-causing mutations complement the essential function(s) of murine FUS. Replacement of murine FUS with mutant, but not wild-type, human FUS causes stress-mediated induction of chaperones, decreased expression of ion channels and transporters essential for synaptic function, and reduced synaptic activity without loss of nuclear FUS or its cytoplasmic aggregation. Most strikingly, accumulation of mutant human FUS is shown to activate an integrated stress response and to inhibit local, intra-axonal protein synthesis in hippocampal neurons and sciatic nerves. Collectively, our evidence demonstrates that human ALS/FTD-linked mutations in FUS induce a gain of toxicity that includes stress-mediated suppression in intra-axonal translation, synaptic dysfunction, and progressive age-dependent motor and cognitive disease without cytoplasmic aggregation, altered nuclear localization, or aberrant splicing of FUS-bound pre-mRNAs.

**Video Abstract:**

## Highlights

• Toxicity, not loss of function, of ALS/FTD-linked mutant FUS drives disease
• Intra-axonal protein synthesis is inhibited by ALS/FTD-causing mutants in FUS
• Toxicity from ALS/FTD-linked mutants in FUS induces an integrated stress response
• ALS/FTD mutants in FUS reduce synaptic activity without loss of nuclear FUS

## Graphical Abstract

**Figure:**
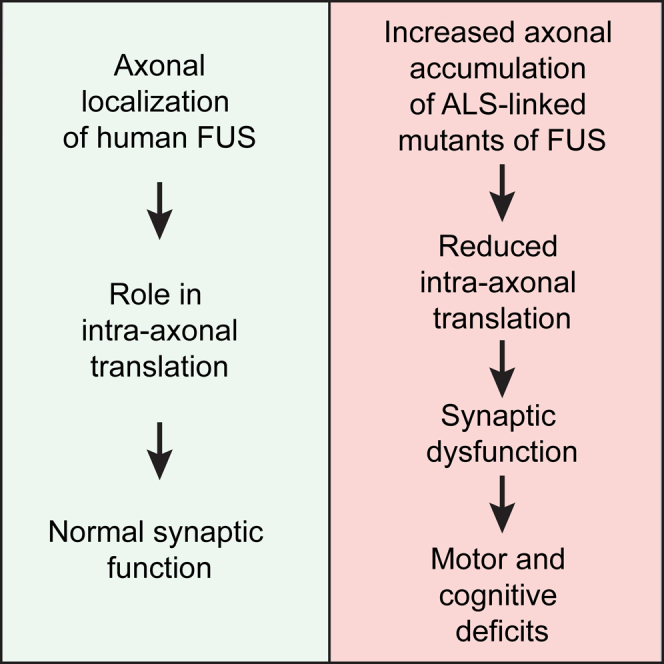

## Teaser

Mutations in FUS are causative of ALS and frontotemporal dementia (FTD). López-Erauskin et al. show that disease-causing mutant FUS inhibits intra-axonal protein synthesis and provokes hippocampal synaptic loss and dysfunction without loss of nuclear FUS function or FUS aggregation.

## Introduction

Amyotrophic Lateral Sclerosis (ALS) is a neurodegenerative disorder leading to paralysis from death of motor neurons. Mutations in the gene encoding the RNA binding protein FUS (fused in sarcoma) are causative of cases of familial ALS (Kwiatkowski et al., 2009, Vance et al., 2009) as well as instances of frontotemporal dementia (FTD) (Broustal et al., 2010, Van Langenhove et al., 2010, Yan et al., 2010). FUS is present in the pathological inclusions of patients with FUS-mediated ALS and most of FTD instances without TDP-43 or Tau-containing aggregates, accounting for about 10% of the frontotemporal lobar degeneration (FTLD) cases, known as FTLD-FUS (Mackenzie et al., 2010).

FUS-containing aggregates have been reported in both the nucleus and the cytoplasm of neurons and glial cells in the central nervous system (CNS) of patients with *FUS* mutations or sporadic disease (Belzil et al., 2011, Chio et al., 2011, DeJesus-Hernandez et al., 2010, Kim et al., 2014b, Rademakers et al., 2010, Vance et al., 2009) as well as neurodegenerative conditions including Huntington’s disease (Doi et al., 2008). The relocalization of FUS from the nucleus to the cytoplasm is incomplete, with at least some nuclear retention in neurons with FUS inclusions (Munoz et al., 2009, Neumann et al., 2009, Rademakers et al., 2010).

The 526-amino-acid FUS protein includes a glycine-rich, low-complexity, prion-like domain and a C-terminal, non-classical PY nuclear localization signal (PY-NLS) in which most of the ALS/FTLD-linked mutations are clustered (Da Cruz and Cleveland, 2011). Like TDP-43, in most cell types FUS is mainly nuclear (Andersson et al., 2008). Beyond nuclei, FUS has been reported in neurons to be enriched in dendrites (Belly et al., 2005, Yasuda et al., 2013), spines (Fujii et al., 2005), in close proximity of presynaptic vesicles (Schoen et al., 2016), and at the neuromuscular junctions (So et al., 2018). Reduction (Udagawa et al., 2015) or deletion (Hicks et al., 2000) of *Fus* in mouse hippocampal neurons causes abnormal spine morphology and density, consistent with a role for FUS in modulation of neuronal plasticity or synaptic activity—potentially through the alteration of mRNA stability (Udagawa et al., 2015) or transport and local translation in neurons (Fujii et al., 2005). While some studies have reported that a proportion of wild-type FUS is recruited to cytoplasmic stress granules, others have found that only FUS mutants localize to stress granules (Aulas and Vande Velde, 2015).

FUS has been proposed to participate in a range of cellular processes including transcription, splicing, RNA localization and degradation, and DNA damage (Lagier-Tourenne et al., 2010). Genome-wide analyses have reported that FUS binds between 5,000 and 8,000 mammalian RNA targets (reviewed in Nussbacher et al., 2015) and that deletion of the gene is associated with alterations in levels and splicing of ∼1,000 mRNAs (Honda et al., 2013, Ichiyanagi et al., 2016, Kapeli et al., 2016, Lagier-Tourenne et al., 2012). Nevertheless, deletion of *Fus* from outbred mice (Kuroda et al., 2000) or from inbred mature mouse motor neurons does not cause their degeneration (Sharma et al., 2016). Efforts to model FUS-mediated disease in rodents have used heterologous promoters (that do not fully recapitulate the pattern of expression of endogenous FUS) to drive, in most cases, elevated levels of human wild-type (Mitchell et al., 2013, So et al., 2018) or mutant FUS (Huang et al., 2011, Qiu et al., 2014, Sephton et al., 2014, Sharma et al., 2016, Shelkovnikova et al., 2013, Shiihashi et al., 2016, Verbeeck et al., 2012). Others have used heterozygous or homozygous removal of the nuclear localization domain of mouse *Fus* (Devoy et al., 2017, Scekic-Zahirovic et al., 2017, Scekic-Zahirovic et al., 2016). Both sets of efforts have suggested that gain of toxicity is a component of pathogenesis. However, the identity(ies) of possible acquired toxicities remains unknown, and whether loss of function contributes to disease is not established.

We have now generated humanized FUS mice in which the full-length human *FUS* gene, encoding wild-type or ALS-linked mutations, replaces murine *Fus*. With expression levels close to the normal levels of endogenous FUS, wild-type or mutant FUS mimics the predominantly nuclear localization of endogenous FUS and complements its essential function(s). Mutant FUS is shown to cause progressive motor and cognitive deficits—without detectable cytoplasmic aggregation—accompanied by RNA alterations in expression that are driven by a gain-of-toxicity rather than a loss of FUS function. Most strikingly, mutant FUS is shown to inhibit local intra-axonal protein translation, drive synaptic loss, elevate stress-induced chaperones, and suppress RNAs encoding ion channels and transporters essential for synaptic function.

## Results

### Mice with Human FUS Expression Mimicking Murine FUS

**Figure 1:**
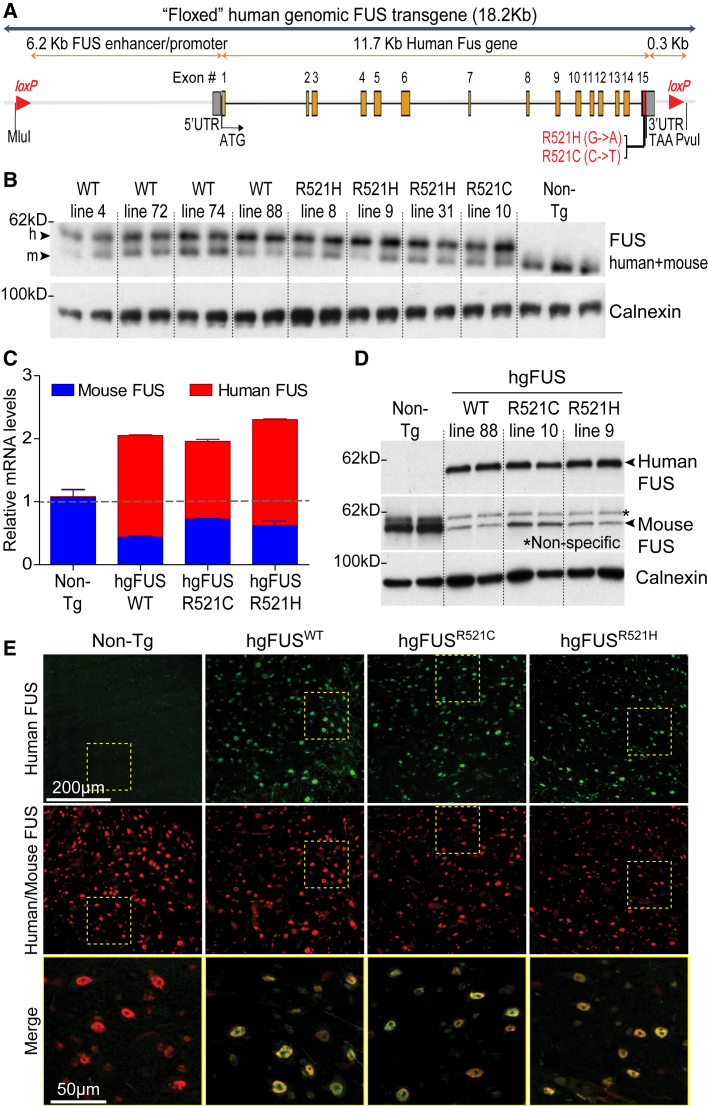
Expression Levels and Cellular Pattern of the Human FUS Transgene Mimics That of Endogenous Protein with an Auto-regulation Mechanism (A) Schematic of the human BAC containing human *FUS* gene wild-type (WT) or either ALS-linked mutation R521C or R521H. (B) Protein levels of the 54 and 53kDa human and mouse FUS using an antibody recognizing both species with equal affinity. Calnexin was used as a loading control. Each lane represents an independent mouse spinal cord. (C) Expression levels of total (mouse, blue bars and human, red bars) *FUS* RNAs in Non-Tg, hg*FUS*^WT^, hg*FUS*^R521C^, and hg*FUS*^R521H^ spinal cords. Data are represented as mean ± SEM (n = 3 per group). See also Figure S1. (D) Protein levels of the human FUS transgene using an antibody specific to the human protein and mouse FUS using an antibody specifically recognizing the mouse protein in 2-month-old animals (^∗^ non-specific band). Each lane represents an independent mouse spinal cord per transgenic line. Calnexin was used as a loading control. (E) Lumbar spinal cord sections from 2-month-old Non-Tg, hg*FUS*^WT^, hg*FUS*^R521C^, and hg*FUS*^R521H^ mice immunostained for human FUS transgene (green) and total FUS (red). Scale bars, 200 and 50 μm.

Transgenic mice were generated in a C57BL/6 congenic background to express wild-type (WT) FUS or either of two ALS-linked mutations (R521C and R521H), each transcribed from the endogenous human *FUS* promoter (Figure 1A). Twenty-six founder mice were obtained expressing human FUS WT (hg*FUS*^WT^), R521C (hg*FUS*^R521C^), or R521H (hg*FUS*^R521H^) (Figure S1A). Eight lines were established (Figure S1B).

Accumulated human FUS levels were comparable in most lines using an antibody recognizing both human and mouse FUS proteins with equivalent affinity (Figures 1B, S1D, and S1E). Endogenous FUS was reduced by more than 50% at mRNA (Figures 1C and S1C) and protein (Figures 1B and 1D) levels, resulting in overall FUS protein levels similar to endogenous mouse FUS levels in non-transgenic (Non-Tg) animals (Figure 1B). Lines with matching levels (line 88 for WT, line 10 for R521C, and line 9 for R521H; Figures 1C and 1D) and expression pattern of human FUS (determined in spinal cord sections stained with an antibody specifically recognizing human, but not mouse, FUS) mimicking that of the endogenous mouse FUS (Figure 1E) were further characterized.

### Age-Dependent Motor Deficits Without Nuclear FUS Mislocalization or Cytoplasmic Aggregation in ALS/FTD-Linked Mutant FUS Mice

**Figure 2:**
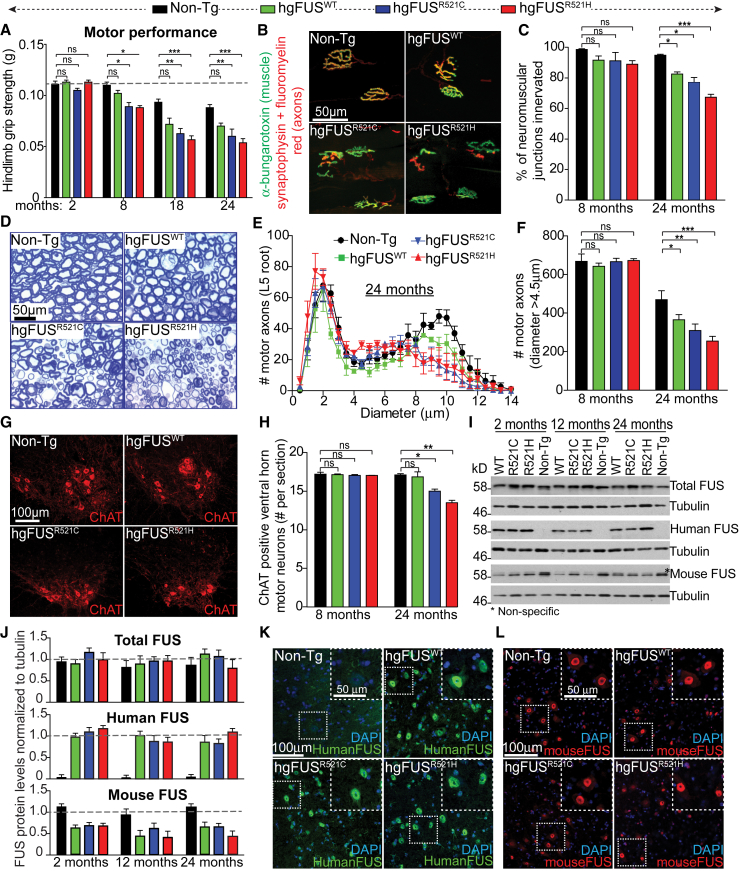
ALS-Linked FUS Mutants Show Age-Dependent Progressive Motor Impairments without Nuclear Mislocalization or Aggregation of FUS (A) Hindlimb grip strength measured up to 24 months. Data are represented as mean ± SEM (n ≥ 12 per group). ^∗^p < 0.05, ^∗∗^p < 0.01, ^∗∗∗^p < 0.001, ns: non-significant using one-way ANOVA. (B) Gastrocnemius muscle sections from 24-month-old Non-Tg, hg*FUS*^WT^, hg*FUS*^R521C^, and hg*FUS*^R521H^ mice with axons immunostained using a synaptophysin antibody and FluoroMyelin™ red (red) and muscle endplates using α-bungarotoxin (green). Scale bar, 50 μm. (C) NMJ innervation of the gastrocnemius from 8- and 24-month-old Non-Tg, hg*FUS*^WT^, hg*FUS*^R521C^, and hg*FUS*^R521H^ mice. Data are represented as mean ± SEM (n ≥ 3 mice per group). ^∗^p < 0.05, ^∗∗∗^p < 0.001, two-sided unpaired Student’s t test. (D) Lumbar motor axons from 24-month-old Non-Tg, hg*FUS*^WT^, hg*FUS*^R521C^, and hg*FUS*^R521H^ mice. Scale bar, 50 μm. (E) Size distribution per motor axon in the L5 motor root of 24-month-old Non-Tg, hg*FUS*^WT^, hg*FUS*^R521C^, and hg*FUS*^R521H^ mice. Data are represented as mean ± SEM (n ≥ 3 per group). ^∗^p < 0.05, ^∗∗^p < 0.01 and ^∗∗∗^p < 0.001, two-sided unpaired Student’s t test. (F) Total number of α-motor axons (caliber size > 4.5 μm) in the L5 motor root of 8- and 24-month-old Non-Tg, hg*FUS*^WT^, hg*FUS*^R521C^, and hg*FUS*^R521H^ mice. Data are represented as mean ± SEM (n ≥ 3 per group). (G) Lumbar spinal cord sections from 24-month-old Non-Tg, hg*FUS*^WT^, hg*FUS*^R521C^, and hg*FUS*^R521H^ mice immunostained using a ChAT antibody to reveal cholinergic motor neurons. Scale bar, 100 μm. (H) Total number of ChAT-positive motor neurons in lumbar spinal cords of 8- and 24-month-old Non-Tg, hg*FUS*^WT^, hg*FUS*^R521C^, and hg*FUS*^R521H^ mice. Data are represented as mean ± SEM (n ≥ 3 per group). ^∗^p < 0.05, ^∗∗^p < 0.01, two-sided unpaired Student’s t test. (I) Total human and/or mouse FUS protein levels in spinal cords of 2-, 12-, and 24-month-old hg*FUS*^WT^, hg*FUS*^R521C^, hg*FUS*^R521H^, and Non-Tg mice. Tubulin was used as a loading control. Asterisk illustrates a non-specific band recognized by the antibody against mouse FUS. (J) Quantification of immunoblots shown in (I). Data are represented as mean ± SEM from two independent experiments. (K and L) Lumbar spinal cord sections from 24-month-old Non-Tg, hg*FUS*^WT^, hg*FUS*^R521C^, and hg*FUS*^R521H^ mice immunostained using an antibody recognizing specifically (K) human or (L) mouse FUS. DNA is stained with DAPI. Scale bars, 100 μm and 50 μm (inset). See also Figure S2.

Motor performance of all three FUS (hg*FUS*^WT^, hg*FUS*^R521C^, and hg*FUS*^R521H^) mouse lines at 2 months of age was undistinguishable from that of Non-Tg littermates. By 8 months, both mutant FUS lines had developed significant reduction in hindlimb grip strength compared to age-matched Non-Tg and hg*FUS*^WT^ littermates (Figure 2A). Motor dysfunction in both mutant lines was further exacerbated during aging, reaching a loss of grip strength of over 50% by 18 months. Loss of motor performance was accompanied by age-dependent loss of muscle innervation (Figures 2B and 2C), α-motor axons (Figures 2D–2F), and spinal cord motor neurons (Figures 2G and 2H). There was also a modest decline in the number of innervated neuromuscular junctions (NMJs) (Figure 2C) and α-motor axons in age-matched hg*FUS*^WT^ mice relative to Non-Tg animals (Figures 2E and 2F).

Loss of motor neurons in mutant animals was accompanied by increased activation of astrocytes (Figures S2A and S2C) and microglia (Figures S2B and S2C) in spinal cord ventral horns of hg*FUS*^R521C^ and hg*FUS*^R521H^ mice (scored by GFAP and Iba1 immunoreactivity, respectively). FUS protein levels remained unchanged up to 24 months of age across all genotypes (Figures 2I and 2J). Similarly, although elevated expression of mutant FUS^R521C^ driven by a prion promoter has been proposed to cause accumulation of DNA damage in mouse spinal cords (Qiu et al., 2014), no such damage (measured by γH2A.X and 53BP1 co-localization) was observable in aged, diseased hg*FUS*^R521C^ and hg*FUS*^R521H^ mice (Figure S2D), although it was obvious in the spinal cords of γ-irradiated animals.

Age-dependent motor deficits in ALS-causing mutant FUS lines were not accompanied by cytoplasmic redistribution of either human (Figures 2K and S2E) or mouse (Figures 2L and S2E) FUS or FUS aggregates (Figures 2K–2L and S2F) in 24-month-old spinal cords. Taken together, neither FUS aggregates nor redistribution to the cytoplasm are necessary for age-dependent motor deficits in the hg*FUS*^R521C^ and hg*FUS*^R521H^ lines.

### ALS/FTD-Linked Mutations in FUS Drive Age-Dependent Cognitive Deficits and Synaptic Loss

**Figure 3:**
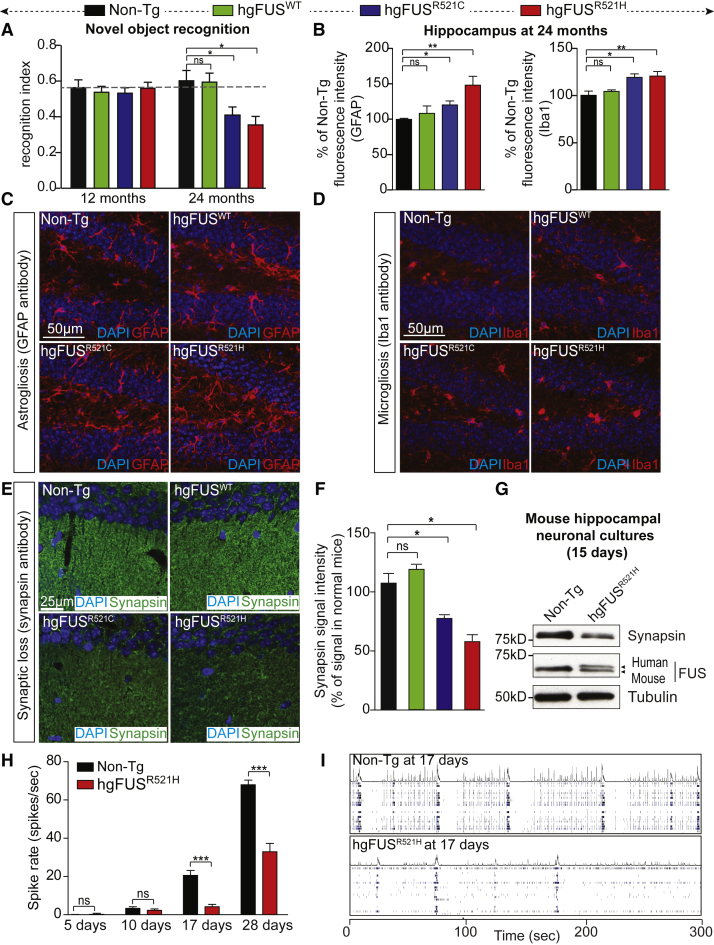
Age-Dependent Progressive Cognitive Impairments, Astrogliosis, and Microgliosis in Hippocampus of Mice Expressing ALS-Linked FUS Mutants (A) Novel object recognition was measured in 12- and 24-month old Non-Tg, hg*FUS*^WT^, hg*FUS*^R521C^, and hg*FUS*^R521H^ mice. Data are represented as mean ± SEM (n = 15 per group). ^∗^p < 0.05 using one-way ANOVA. See also Figure S3. (B) Quantification of the GFAP and Iba1 relative fluorescence intensities in hippocampal sections of 24-month-old Non-Tg, hg*FUS*^WT^, hg*FUS*^R521C^, and hg*FUS*^R521H^ mice. The bar graph represents mean ± SEM (n = 3 per group). ^∗^p < 0.05, ^∗∗^p < 0.01, two-sided unpaired Student’s t test. (C and D) Hippocampal sections from 24-month-old Non-Tg, hg*FUS*^WT^, hg*FUS*^R521C^, and hg*FUS*^R521H^ mice immunostained using an antibody detecting activated (C) astrocytes (GFAP) or (D) microglia (Iba1). DNA is stained with DAPI. Scale bar, 50 μm. (E) Hippocampus from 24-month-old Non-Tg, hg*FUS*^WT^, hg*FUS*^R521C^, and hg*FUS*^R521H^ mice immunostained to reveal synapses (green) using a synapsin antibody and DAPI for nuclei. Scale bar, 25 μm. (F) Quantification of the synapsin relative fluorescence intensity shown in (E). The bar graph represents mean ± SEM (n = 3 per group). ^∗^p < 0.05, two-sided unpaired Student’s t test. (G) Synapsin and FUS protein levels in hippocampal neuronal extracts from mutant hg*FUS*^R521H^ or Non-Tg mice (at 15 days of culture). Tubulin antibody was used as loading control. (H) Spontaneous neuronal spike rate (spikes per s) measured by multi-electrode arrays (MEA) in hippocampal neurons from mutant hg*FUS*^R521H^ compared to Non-Tg mice cultured under basal conditions. Data are represented as mean ± SEM (n = 4–8 numbers of wells per experiment from three independent experiments). ^∗∗∗^p < 0.001, two-sided unpaired Student’s t test. (I) Raster plots from MEA recordings showing neuronal spikes and burst of spikes in wild-type and mutant hg*FUS*^R521H^ hippocampal neurons for 300 s. Each lane represents spikes per s recorded per electrode.

Age-dependent deficits in cognition and memory were developed in hg*FUS*^R521C^ and hg*FUS*^R521H^ mice, including novel object recognition (Figure 3A), spatial memory (Figure S3A), sociability (Figures S3B and S3C) and anxiety (Figure S3D). These alterations were accompanied by modest increases in astrogliosis (Figures 3B and 3C) and microgliosis (Figures 3B and 3D), as well as a significant loss of synapses (marked by loss of synapsin; Figures 3E and 3F) in the hippocampus of aged, 24-month-old mice, without loss of NeuN positive neurons. Mutant-FUS-dependent loss of synapsin was recapitulated in cultured primary hippocampal neurons (Figure 3G). Neuronal activity measurements (using multi-electrode arrays [MEA]; Chailangkarn et al., 2016) revealed age- and mutant-dependent synaptic dysfunction, with a 50% decrease in spike rate developing by 28 days of culture (Figures 3H and 3I).

### Progressive Neurodegeneration in Humanized Mutant FUS Mice

**Figure 4:**
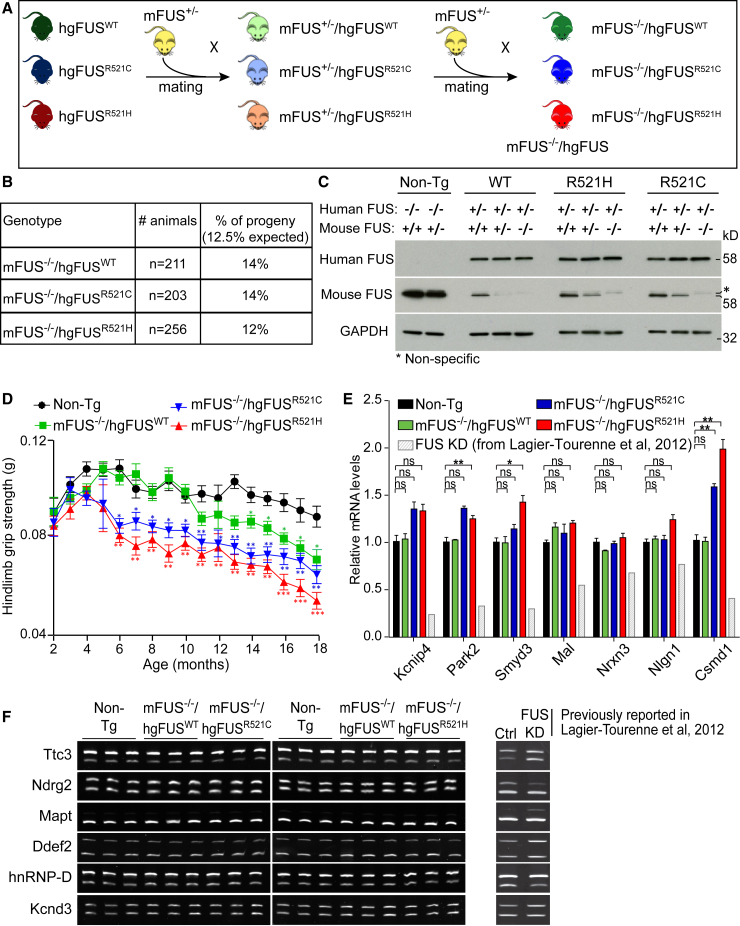
Progressive Motor Deficits Associated with ALS-Linked FUS Mutants Without Reduction in FUS Function (A) Schematic of the two-round mating of hg*FUS* mice with FUS mice heterozygous for mouse FUS (m*Fus*^+/−^) to produce animals in which all FUS is human wild-type or mutant (m*Fus*^−/−^/hg*FUS*). (B) Number and Mendelian ratio of animals obtained from the two-step mating. (C) Human and mouse FUS protein accumulation in 2-month old mouse spinal cords using FUS antibodies specific for human and mouse proteins. GAPDH was used as a loading control. Asterisk illustrates a non-specific band detected with the mouse-specific FUS antibody. (D) Hindlimb grip strength measured bi-weekly from 2 to 18 months of age. Data are represented as mean ± SEM (n ≥ 15 per group). ^∗^p < 0.05, ^∗∗^p < 0.01 and ^∗∗∗^p < 0.001 using one-way ANOVA. (E) Expression levels of candidate genes (known to be reduced in the CNS of mice depleted of FUS [light gray], previously reported in Lagier-Tourenne et al., 2012; figure 3I) in 18-month-old Non-Tg, m*Fus*^−/−^/hg*FUS*^WT^, m*Fus*^−/−^/hg*FUS*^R521C^, and m*Fus*^−/−^/hg*FUS*^R521H^ mouse spinal cords. Data are represented as mean ± SEM (n ≥ 3 per group). ^∗^p < 0.05, ^∗∗^p < 0.01, two-sided unpaired Student’s t test. (F) Splicing profile of candidate genes (known to be altered in the CNS of mice depleted of FUS, previously reported in Lagier-Tourenne et al., 2012; figure 4C) in 18-month-old Non-Tg, m*Fus*^−/−^/hg*FUS*^WT^, m*Fus*^−/−^/hg*FUS*^R521C^, and m*Fus*^−/−^/hg*FUS*^R521H^ mouse spinal cords. See also Figure S4.

We next generated humanized FUS mice (m*Fus*^−/−^/hg*FUS* mice) in which the sole source of FUS was human FUS. This was achieved by mating to produce mice that expressed our wild-type or mutant human FUS-encoding transgenes but in which both endogenous *Fus* alleles were inactivated (Figure 4A). While homozygous disruption of murine *Fus* is lethal in C57BL/6 mice, as previously reported (Hicks et al., 2000), lethality was completely rescued by expression of human wild-type or mutant FUS, and humanized FUS mice (either WT or mutants) were born with the expected Mendelian ratios (Figures 4B and 4C). Nevertheless, despite comparable human FUS mRNA (Figure S4A) and protein levels (Figure 4C), beginning at 6 months of age, hg*FUS*^R521C^ or hg*FUS*^R521H^ mice developed progressive motor deficits including age-dependent loss of hindlimb grip strength (Figure 4D) and loss of spinal cord ChAT-positive motor neurons (Figure S4B). Remarkably, reduction or elimination of endogenous *Fus* did not exacerbate mutant FUS-dependent motor deficits and neurotoxicity (Figures 2 and 4).

### ALS/FTD-Mediated Disease Without Loss of Normal FUS Function

To determine whether age-dependent loss of FUS nuclear function contributes to FUS-mediated disease in aged, humanized FUS mice, levels (Figure 4E) and splicing (Figure 4F) of the genes most affected by FUS depletion in mice (Lagier-Tourenne et al., 2012) were assessed. Expression levels of *Kcnip4*, *Park2*, *Smyd3*, *Mal*, *Nrxn3*, *Nlgn1*, and *Csmd1* were significantly decreased in the CNS of mice treated with reduced FUS following antisense oligonucleotide-mediated degradation of *Fus* mRNAs (Figure 4E; Lagier-Tourenne et al., 2012). However, a modest increase, rather than a reduction, was seen in the expression of these genes in spinal cords of humanized hg*FUS*^R521C^ and hg*FUS*^R521H^ mice compared to Non-Tg and hg*FUS*^WT^ mice depleted of endogenous FUS (Figure 4E).

Genome-wide expression analysis (RNA-seq; Figure S4C) of aged, humanized mutant FUS spinal cords did not significantly overlap with the changes identified upon FUS depletion in adult mouse spinal cords (Kapeli et al., 2016) (Figure S4E). Similarly, the most common splicing changes identified upon depletion of FUS (Lagier-Tourenne et al., 2012) were not observed in the humanized FUS mouse spinal cords (Figure 4F). Global splicing analysis using the RNA-mediated oligonucleotide annealing, selection, and ligation with next-generation sequencing (RASL-seq) method (which permitted quantitative profiling of 3,859 alternative splicing events that correspond to exon inclusion or skipping events conserved between mouse and human; Scekic-Zahirovic et al., 2016, Sun et al., 2015, Zhou et al., 2012) did not reveal significant splicing alterations in aged, humanized mutant FUS spinal cords (Figure S4D). Altogether, these results demonstrate that the age-dependent motor deficits associated with expression of ALS-linked FUS mutant in humanized FUS mice cannot be caused by a loss of function of FUS in regulating gene expression and/or splicing.

### A Mutant FUS-Dependent Gain of Toxicity RNA Signature Affecting Protein Synthesis

**Figure 5:**
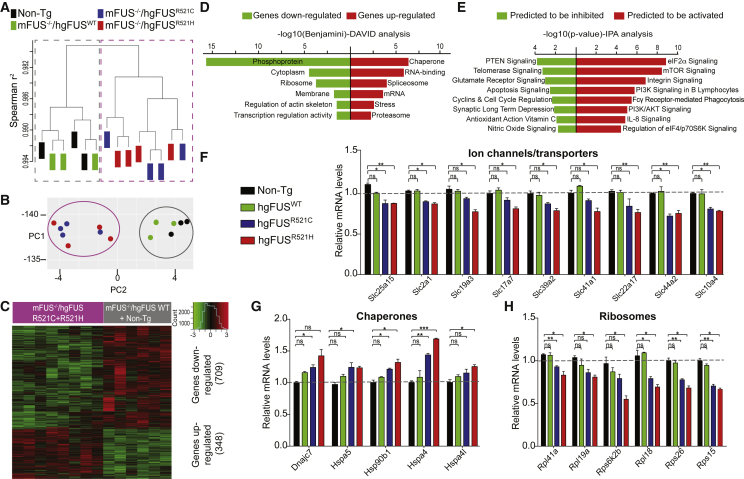
ALS-Linked Mutations in FUS Cause a Mutant-Dependent Signature of RNAs that Includes a Reduction in mRNAs for Ion Channels and Transporters and Ribosomal Proteins and an Increase in mRNAs Encoding Chaperones (A) Unsupervised hierarchical clustering of all expressed genes in spinal cords of 18-month-old Non-Tg, m*Fus*^−/−^/hg*FUS*^WT^, m*Fus*^−/−^/hg*FUS*^R521C^, and m*Fus*^−/−^/hg*FUS*^R521H^ mice identified by RNA-seq analysis. (B) Principal component analysis (PCA) of differentially expressed genes in 18-month-old Non-Tg, m*Fus*^−/−^/hg*FUS*^WT^, m*Fus*^−/−^/hg*FUS*^R521C^, and m*Fus*^−/−^/hg*FUS*^R521H^ spinal cords. (C) Heatmap with hierarchical clustering of 1,057 genes differentially expressed in 18-month-old hg*FUS*^R521C^ and hg*FUS*^R521H^ mice compared to age-matched Non-Tg and m*Fus*^−/−^/hg*FUS*^WT^ spinal cords. (D) Functional analysis using DAVID software of the differentially expressed genes revealing the most enriched gene group changes in m*Fus*^−/−^/hg*FUS*^R521C^ and m*Fus*^−/−^/hg*FUS*^R521H^ mice compared to age-matched Non-Tg and m*Fus*^−/−^/hg*FUS*^WT^ animals. (E) Functional analysis using the Ingenuity Pathway Analysis (IPA) software of the differentially expressed genes revealing the most enriched gene group changes in m*Fus*^−/−^/hg*FUS*^R521C^ and m*Fus*^−/−^/hg*FUS*^R521H^ mice compared to age-matched Non-Tg and m*Fus*^−/−^/hg*FUS*^WT^ animals. (F) Reduced expression of ion channels and transporters essential for synaptic function in 18-month-old m*Fus*^−/−^/hg*FUS*^R521C^ and m*Fus*^−/−^/hg*FUS*^R521H^ spinal cords compared to age-matched Non-Tg and m*Fus*^−/−^/hg*FUS*^WT^, validated by qRT-PCR. Data are represented as mean ± SEM (n ≥ 3 per group). ^∗^p < 0.05, ^∗∗^p < 0.01, two-sided unpaired Student’s t test. See also Figure S5A. (G) Increased expression of chaperones in 18-month-old m*Fus*^−/−^/hg*FUS*^R521C^ and m*Fus*^−/−^/hg*FUS*^R521H^ spinal cords compared to age-matched Non-Tg and m*Fus*^−/−^/hg*FUS*^WT^, validated by qRT-PCR. Data are represented as mean ± SEM (n ≥ 3 per group). ^∗^p < 0.05, ^∗∗^p < 0.01 and ^∗∗^p < 0.001, two-sided unpaired Student’s t test. See also Figure S5B. (H) Reduced expression of genes encoding ribosomes or other components of the translation machinery in 18-month-old m*Fus*^−/−^/hg*FUS*^R521C^ and m*Fus*^−/−^/hg*FUS*^R521H^ spinal cords compared to age-matched Non-Tg and m*Fus*^−/−^/hg*FUS*^WT^, validated by qRT-PCR. Data are represented as mean ± SEM (n ≥ 3 per group). ^∗^p < 0.05 and ^∗∗^p < 0.01, two-sided unpaired Student’s t test. See also Figure S5C.

RNA expression profiles from spinal cords of humanized mutant FUS mice were used to test whether ALS-linked mutations produced expression changes that reflected gain of aberrant function (Figures 5 and S4E). RNA profiles from normal (Non-Tg) and humanized hg*FUS*^WT^ mice were almost indistinguishable. However, both humanized mutant FUS lines had highly distinct RNA profiles determined with unsupervised hierarchical clustering (Figure 5A) and principal component analysis (PCA) (Figure 5B), with 1,057 significant expression changes (defined by p < 0.05 adjusted for multiple testing; Table S1) and with 709 down and 348 upregulated genes (Figure 5C) relative to age-matched Non-Tg or humanized hg*FUS*^WT^ littermates. These changes (Figures 5D and 5E) included altered signaling pathways for protein translation mediated by eIF2α, a crucial translation initiation factor which forms a complex with components including eIF2B, GTP, and the initiating methionine tRNA (Kapur et al., 2017). mTOR and glutamate receptor signaling pathways (Figure 5E) were also affected.

mRNAs encoding multiple ion channels and transporters essential for synaptic function were decreased in spinal cord extracts of both humanized mutant FUS lines (Figure S5A), consistent with mutant FUS-dependent synaptic dysfunction (Figures 3E and 3F). Further tests using RT-qPCR confirmed reductions in ion channel and transporter components tested (reductions in nine are shown in Figure 5F). These changes were accompanied by the upregulation of ten genes encoding chaperones (Figure S5B), five of which were further validated by qRT-PCR (Figure 5G). Genes encoding ribosomal proteins represented some of the most downregulated genes, including sixteen components of the translation machinery (Figures 5H and S5C).

### Stress-Mediated Inhibition of Intra-axonal Protein Synthesis from ALS/FTD-Linked Mutations in FUS

**Figure 6:**
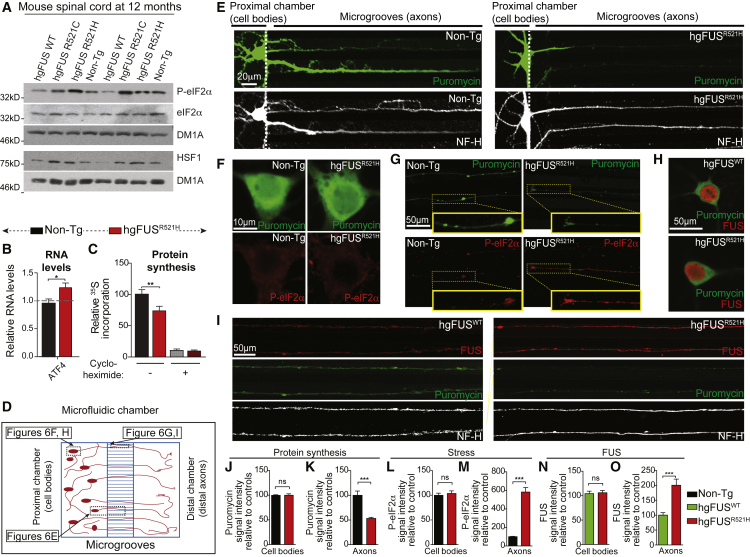
ALS-Linked Mutations in FUS Cause Stress-Mediated Reduction in Axonal Protein Synthesis (A) Immunoblot demonstrating increased stress mediated by mutant FUS at early symptom onset using the phosphorylated form of eIF2α antibody (P-eIF2α) and HSF1 antibody in 12-month-old hg*FUS*^R521C^ and hg*FUS*^R521H^ spinal cords compared to age-matched Non-Tg and hg*FUS*^WT^. eIF2α and tubulin antibodies were used as loading controls. See also quantification in Figures S6A and S6B. (B) Expression of *Atf4* in primary hippocampal neuronal cultures from hg*FUS*^R521H^ compared to Non-Tg mice. Data are represented as mean ± SEM (n ≥ 3 biological replicates per group). ^∗^p < 0.05, two-sided unpaired Student’s t test. (C) Quantification of the amount of ^35^S incorporated into newly translated proteins in hippocampal neurons cultured for 15 days. Cycloheximide (25 μg/mL) addition was used as a positive control for complete protein translation inhibition. Data are represented as mean ± SEM (n = 4 biological repeats). ^∗∗^p < 0.01, two-sided unpaired Student’s t test. (D) Schematic of the experimental design using microfluidic compartmented chambers to distinguish neuronal cell bodies from axons. (E–G) Hippocampal neurons isolated from hg*FUS*^R521H^ or Non-Tg mice (from four independent experiments with 3 or 4 mouse embryos per genotype per experiment) cultured in compartmented chambers and treated with a 10-min pulse of puromycin to label protein translation sites using puromycin antibody (green) with a neurofilament NF-H (white) antibody to stain axons (E) or with an antibody recognizing the phosphorylated form of eIF2α (P-eIF2α) (red) in (F) cell bodies and (G) neuronal processes. Scale bars, 10 μm and 50 μm, respectively. (H and I) Hippocampal neurons isolated from hg*FUS*^R521H^ and hg*FUS*^WT^ mice cultured in compartmented chambers immunostained for FUS (using FUS antibody in red) and protein translation (using puromycin antibody in green) in (H) cell bodies and (I) axonal processes (using NF-H antibody, white). Scale bar, 50 μm. (J–M) Quantification of the puromycin relative fluorescence intensity (protein translation) in (J) cell bodies (green signal in Figure 6F) and (K) axons (green signal in Figure 6G); quantification of P-eiF2α fluorescence intensity (stress) in (L) cell bodies (red signal in Figure 6F) and (M) axons (red signal in Figure 6G) of Non-Tg and hg*FUS*^R521H^ hippocampal neurons cultured in microfluidic chambers. The bar graph represents mean ± SEM (n ≥ 30 axons or cell bodies per group from four independent experiments). ^∗∗∗^p < 0.001, two-sided unpaired Student’s t test. (N and O) Quantification of FUS relative fluorescence intensity in (N) cell bodies (red signal in Figure 6H) and (O) axons (red signal in Figure 6I). The bar graph represents mean ± SEM (n ≥ 50 axons or cell bodies per group). ^∗∗∗^p < 0.001, two-sided unpaired Student’s t test.

FUS mutant-dependent effects on protein translation mediated through eIF2α were tested in the nervous system of the humanized FUS mice. Phosphorylation of eIF2α on serine 51 inhibits overall initiation by blocking the activity of its eIF2B guanine exchange factor (Donnelly et al., 2013). Consistent with inhibited translation initiation, elevated levels of phosphorylated eIF2α (P-eIF2α), together with increased levels of HSF1 (heat shock transcription factor 1), were identified in spinal cord protein extracts from FUS adult mutant mice at early symptom onset (Figure 6A, S6A, and S6B).

ATF4 is a key effector of the integrated stress response that drives phosphorylation of eIF2α (Pakos-Zebrucka et al., 2016). Consistent with P-eIF2α dependent protein synthesis inhibition, there was a mutant FUS-dependent increase of *Atf4* mRNA levels in primary mouse hippocampal neurons (Figure 6B). Correspondingly, mRNA translation was reduced (compared with neurons of wild-type mice) by 25% in metabolically labeled (with ^35^S-Met/Cys) cultured hippocampal neurons from mutant FUS mice (Figure 6C).

Recognizing that puromycin incorporation into nascent polypeptides enables direct visualization of actively translating ribosomes (using an anti-puromycin antibody; tom Dieck et al., 2015), we cultured hippocampal neurons in compartmented microfluidic chambers that allow unambiguously distinguishing cell bodies from processes, prior puromycin pulse-labeling (Figures 6D and 6E). Surprisingly, translation was found to be predominantly reduced in processes (Figures 6E, 6G, 6I, and 6K) relative to cell bodies (Figures 6F, 6H, 6J, S6C, and S6D). This reduction was accompanied by increased phosphorylation of eIF2α in axons (Figures 6G and 6M), with no differences observed in cell bodies (Figures 6F and 6L). No cell death was detected at any time point (Figures S6E and S6F).

While a majority of FUS localized intranuclearly (Figure 6H, red), both wild-type and mutant human FUS were present in axons of hippocampal neurons cultured in compartmented microfluidic chambers (Figure 6I). FUS was not elevated in the cytoplasm of mutant neuronal cell bodies (Figures 6H and 6N). However, relative to wild-type FUS, significantly more mutant FUS accumulated within axons (Figures 6I and 6O). Since measurement of the volume covered by the axonal network in our hippocampal neuronal cultures revealed that it was at least 6 times that of their cell bodies and dendrites, this strong reduction in intra-axonal protein translation is likely to be sufficient to account for the 25% decreased protein synthesis measured in the total protein extract (Figure 6C).

**Figure 7:**
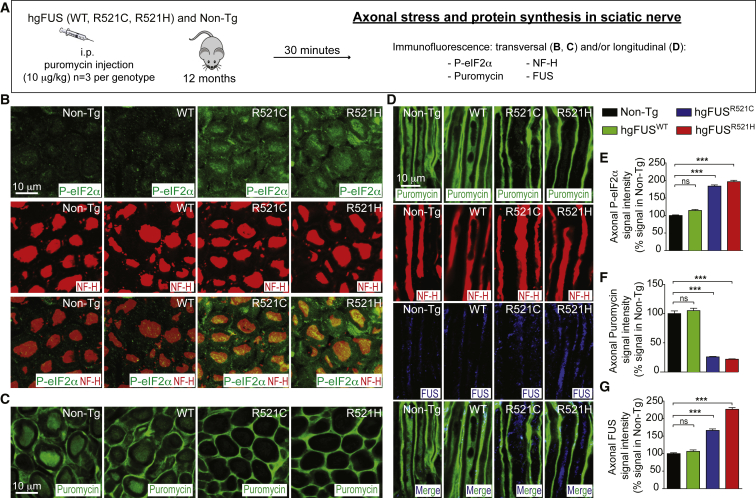
ALS-Linked Mutations in FUS Cause Stress-Mediated Reduction in Local Axonal Protein Synthesis within Sciatic Nerve Axons of Mutant FUS Mice (A) Experimental design to assess stress and local axonal translation in the sciatic nerve of 12-month-old Non-Tg, hg*FUS*^WT^, hg*FUS*^R521C^, and hg*FUS*^R521H^ mice. (B) Transversal sections of mouse sciatic nerves immunostained using P-eIF2α (green) and NF-H (red) antibodies and (C) puromycin (green) antibody. (D) Longitudinal sections of mouse sciatic nerves immunostained using puromycin (green), NF-H (red), and FUS (blue) antibodies. Scale bars, 10 μm. (E–G) Quantification of the relative fluorescence intensity of the (E) P-eIF2α, (F) puromycin, and (G) FUS immunostainings within sciatic nerve axons of 12-month-old Non-Tg, hg*FUS*^WT^, hg*FUS*^R521C^, and hg*FUS*^R521H^ mice. The bar graph represents mean ± SEM (n = 3 animals per group). ^∗∗∗^p < 0.001, two-sided unpaired Student’s t test.

To test mutant FUS effects on local translation in motor axons in mice, puromycin pulse labeling of sciatic nerve axons was achieved by its systemic administration into the mice, as previously reported (Goodman et al., 2011, Khoutorsky et al., 2015; Figure 7A). Increased phosphorylation of eIF2α (green signal by immunofluorescence) was observed in sciatic nerve axons (neurofilament positive; red) in both hg*FUS*^R521C^ and hg*FUS*^R521H^ mice at early symptom onset, but not in age-matched Non-Tg or hg*FUS*^WT^ animals (Figures 7B and 7E). Consistent with our findings in the hippocampal neurons (Figures 6E–6O and S6C–S6D), intra-axonal protein synthesis (revealed by decreased puromycin signal) was almost eliminated (Figures 7C–7D and 7F), along with increased accumulation of ALS-linked mutants of FUS (Figures 7D and 7G) within sciatic nerve axons of the two mutant FUS lines, but not their Non-Tg and hg*FUS*^WT^ littermates. Taken together, our *in vitro* and *in vivo* evidence demonstrates that ALS-linked mutations of FUS impair local protein synthesis within axons.

## Discussion

A key question regarding pathogenic mechanisms in FUS-mediated disease has been whether ALS-linked mutations in *FUS* cause neurodegeneration through a loss and/or gain of toxicity. By producing and analyzing humanized FUS mice in which wild-type or ALS-causing mutations in human *FUS* replace endogenous murine *Fus*, we demonstrate that both human wild-type and ALS/FTD-linked FUS mutants complement the essential functions of murine FUS. When expressed at a level and subcellular distribution that mimic endogenous FUS, mutant human FUS provokes progressive motor and cognitive deficits in mice that are accompanied by RNA alterations driven by a gain of toxicity rather than a loss of function of FUS, but without detectable cytoplasmic aggregation.

While high levels of wild-type FUS cause early lethality in mice (Mitchell et al., 2013, Sephton et al., 2014), our mice expressing wild-type FUS at levels close to the normal level of murine FUS also developed motor deficits during aging. When taken with a report that ALS patients with mutations in the 3′ UTR region of FUS accumulate those variants at increased levels in patient fibroblasts (Sabatelli et al., 2013), it is therefore likely that even a modest increase in FUS (human mutant or wild-type) is sufficient to trigger age-dependent motor deficits.

To an earlier report that FUS depletion from iPSC-derived motor neurons produces a different RNA signature than a disease-linked FUS mutation (Kapeli et al., 2016), our analyses have identified that the overwhelming majority of gene expression alterations associated with age-dependent motor and cognitive deficits in humanized mutant FUS mice do not overlap with those altered upon depletion of FUS (Kapeli et al., 2016, Lagier-Tourenne et al., 2012). Depletion of FUS in the adult mouse CNS leads to more than 300 splicing changes (Kapeli et al., 2016, Lagier-Tourenne et al., 2012) and disrupts non-canonical, U12-dependent splicing of RNAs (Reber et al., 2016) in a neuronal cell line. While cytoplasmic aggregates of an ALS-linked FUS mutant were reported to inhibit U12-mediated splicing of a subset of introns by trapping of snRNAs in aggregates (Reber et al., 2016), and ALS-linked mutant-dependent splicing changes have previously been reported in patient fibroblasts (Sun et al., 2015) as well as in motor neuron precursor cells derived from iPSCs (Ichiyanagi et al., 2016), no significant splicing changes in the nervous system of humanized mutant FUS mice were identified despite initiation of adult-onset disease. The major splicing alterations identified upon *in vivo* FUS depletion (Lagier-Tourenne et al., 2012) were not recapitulated in diseased, mutant humanized FUS mice. Thus, FUS-mediated neurodegeneration in mice cannot be caused primarily by reduced FUS activity, a conclusion fully consistent with the finding that deletion of FUS from mature motor neurons does not provoke disease (Sharma et al., 2016).

Several mechanisms underlying FUS-mediated toxicity have now been proposed, including aggregation and redistribution of mutant FUS into the cytoplasm (Dormann et al., 2012, Mackenzie et al., 2011, March et al., 2016). Morphology and distribution of FUS inclusions vary among the CNS of different neurodegenerative conditions, with FUS inclusions reportedly being nuclear in Huntington’s disease (Doi et al., 2008) but primarily cytoplasmic in ALS and FTLD (King et al., 2015, Mackenzie et al., 2011), with some nuclear retention in even the most affected neurons with FUS inclusions (Munoz et al., 2009, Neumann et al., 2009, Rademakers et al., 2010). To these preceding efforts, we report that age-dependent motor and cognitive deficits develop in humanized mutant FUS mice without detectable human FUS cytoplasmic aggregation. Thus, while we cannot rule out FUS aggregates in the cytoplasm in forms undetectable by our analyses, large aggregates similar to those seen at end-stage disease in patients are not required for disease initiation, consistent with several other neurodegenerative diseases in which large inclusions correlate poorly with either onset or severity of neurodegeneration (Arnold et al., 2013, Arrasate et al., 2004, Kirkitadze et al., 2002, Parone et al., 2013).

Increasing evidence suggests that RNA-binding proteins including TDP-43, FMRP, SMN, and ataxin-2 are involved in the regulation of local protein translation and that local translation is critical for synaptic function (Donlin-Asp et al., 2017). FUS is present not only in soma but also along neuronal dendrites in RNA granules (Belly et al., 2005, Yasuda et al., 2013), spines (Fujii et al., 2005) in close proximity to presynaptic vesicles (Schoen et al., 2016), and at the neuromuscular junctions (So et al., 2018). To this, we now demonstrate that FUS protein is present along axons, including at sites of intra-axonal protein synthesis, and that the accumulation of ALS-linked mutants of FUS is significantly increased along axons and dendrites of hippocampal and sciatic nerve neurons.

FUS has been proposed to play a role at the synapse in spine formation (Fujii et al., 2005) and maturation (Udagawa et al., 2015) of hippocampal neurons, possibly by regulating local mRNA translation of synaptic components such as GluA1 (Udagawa et al., 2015) or RNAs enriched in cell protrusions (Yasuda et al., 2017, Yasuda et al., 2013). A role as a RNA chaperone suppressing RNA folding and repeat-associated translation of UGGAA expanded repeats has also been described in a fly model for ataxia (Ishiguro et al., 2017). While expression of high levels of FUS mutants was reported to provoke a reduction in protein synthesis in neuronal cultures (Murakami et al., 2015) and alter dendritic branching and spines in mice (Sephton et al., 2014), it is not established whether—and, if so, how—mutations in FUS may alter such critical functions. In flies, synaptic transmission at the neuromuscular junctions of *Drosophila* larvae expressing FUS mutants was significantly decreased (Shahidullah et al., 2013). Our efforts provide *in vivo* evidence in the mammalian nervous system that expression of FUS mutants at levels approximating the normal level of endogenous FUS is sufficient to cause stress-mediated reduction in intra-axonal protein synthesis prior to synaptic dysfunction and loss and is therefore likely to contribute to synaptic dysfunction associated with age-dependent motor and cognitive deficits.

Indeed, we have demonstrated FUS mutant-dependent suppression of expression of a wide range of components of the protein translation machinery, including mRNAs from the large and small ribosomal subunits. Correspondingly, FUS may play a role in the translation of a wide range of genes. This contrasts with other RNA-binding proteins whose actions have recently been proposed to exert translational control on a specific subset of genes (examples include ataxin 2 on *RGS8* [Dansithong et al., 2015], FMRP on diacylglycerol kinase kappa [Tabet et al., 2016], or FMRP together with TDP-43 on *Rac1*, *Map1b*, and *GluR1* [Majumder et al., 2016]). Future efforts are now needed to identify the spectrum of axonal RNAs affected by FUS mutations. Added to this, mutations in FUS impair axonal transport, including transport of mitochondria and endoplasmic reticulum vesicles in patient-derived (Guo et al., 2017) and *Drosophila* (Baldwin et al., 2016) motor neurons. It is therefore possible that alterations in axonal transport of RNAs contribute to the reduction in local intra-axonal protein synthesis.

Finally, we find an early activation of the integrated stress response (ISR) pathway with increased phosphorylation of eIF2α (Pakos-Zebrucka et al., 2016, Zhao and Ackerman, 2006). Thus, we propose that ALS-linked mutations in FUS lead to increased accumulation of mutant FUS along axons, including at local intra-axonal translation sites; this in turn provokes local ISR that inhibits local protein synthesis, ultimately impairing neuronal synaptic function. Chemical ISR inhibitors have been reported to increase long-term memory (Sidrauski et al., 2013), reverse cognitive deficits associated with traumatic brain injury (Chou et al., 2017) in rodents and reduce TDP-43 mediated toxicity in flies and neuronal cultures (Kim et al., 2014a). It will now be of high interest to test whether modulating ISR pathways using similar compounds reduces the age-dependent motor and cognitive deficits associated with mutant FUS.

## STAR★Methods

### Key Resources Table

**Table:** 

REAGENT or RESOURCE	SOURCE	IDENTIFIER
**Antibodies**		

Rabbit anti-phosphorylated eIF2α (Ser51)	Cell Signaling Technology	Cat#9721; RRID: AB_330951
Rabbit anti- eIF2α	Cell Signaling Technology	Cat# 9722; RRID: AB_2230924
Mouse anti-FUS (4H11)	Santa Cruz Biotechnology	Cat# sc-47711; RRID: AB_2105208
Rabbit anti-FUS	Bethyl	Cat# A300-302A; RRID: AB_309445
Goat anti-FUS	Bethyl	Cat# A303-839A; RRID: AB_2620190
Rabbit anti-FUS	Proteintech Group	Cat#11570-1-AP; RRID: AB_2247082
Mouse anti-GAPDH (6C5)	Abcam	Cat# ab8245; RRID: AB_2107448
Mouse anti-Glial Fibrillary Acidic Protein (GFAP)	Millipore	Cat# MAB360; RRID: AB_11212597
Rabbit anti-HSF1	Cell Signaling Technology	Cat#4356S; RRID: AB_10695463
Rabbit anti-Hsp90 (C45G5)	Cell Signaling Technology	Cat#4877S; RRID: AB_2233307
Rabbit anti-Iba1	Wako	Cat#019-19741; RRID: AB_839504
Chicken anti-Map2	Novus	Cat# NB300-213; RRID: AB_2138178
Mouse anti-Neurofilament Clone RT97	Millipore	Cat# MAB5262; RRID: AB_95186
Rabbit anti-neurofilament H	Millipore	Cat# ab1989; RRID: AB_91202
Mouse anti-Neurofilament H non-phosphorylated SMI-32	Covance	Cat# SMI-32R-100; RRID: AB_509997
Mouse anti-Nucleophosmin	Zymed-Thermo Fisher Scientific	Cat#32-5200; RRID: AB_2533084
Chicken Neurofilament H	Millipore	Cat# AB5539; RRID: AB_11212161
Chicken anti-NeuN	Millipore	Cat# ABN91; RRID: AB_11205760
Anti-puromycin, clone 12D10	Millipore	Cat# MABE343; RRID: AB_2566826
Anti-puromycin, Alexa Fluor ® 488 Conjugated Antibody	Millipore	Cat# MABE343-AF488; RRID: AB_2736875
Rabbit anti-synapsin-1	Synaptic Systems	Cat#106103
Mouse anti-γH2A.X	Millipore	Cat# 05-636; RRID: AB_309864
Rabbit anti-53BP1	Novus	Cat# NB100-304; RRID: AB_10003037
Rabbit anti-calnexin	Enzo Life Sciences	Cat# SPA-860
Mouse anti-tubulin (clone DM1A)	This paper	N/A
Rabbit anti-human FUS #14080	This paper	N/A
Rabbit anti-mouse FUS #14082	This paper	N/A

**Chemicals, Peptides, and Recombinant Proteins**		

Sylgard 182 Elastomer	Fisher Scientific	Cat#NC9897184
Puromycin	Thermo Fisher Scientific	Cat# A1113803

**Critical Commercial Assays**		

LIVE/DEAD Cell viability assay	Thermo Fisher Scientific	Cat#L3224

**Deposited Data**		

RNaseq data	This paper	GEO: GSE120247

**Experimental Models: Organisms/Strains**		

Mouse: C57BL/6 hg*FUS*^WT^	This paper	N/A
Mouse: C57BL/6 hg*FUS*^R521C^	This paper	N/A
Mouse: C57BL/6 hg*FUS*^R521H^	This paper	N/A
Mouse: C57BL/6 m*Fus*^−/−^	Hicks et al., 2000	N/A

**Oligonucleotides**		

See Table S2 for primer sequences used in this paper	IDT	N/A

**Software and Algorithms**		

Fiji	NIH	https://fiji.sc/download; Fiji, RRID: SCR_002285
GraphPad Prism	GraphPad software	https://www.graphpad.com; GraphPad Prism, RRID: SCR_002798
AxIS (Biosystems Neural Metrics/Offline data analysis tool)	Axion Biosystems	https://www.axionbiosystems.com/products/axis-software; AxIS, RRID:SCR_016308
Bioquant Software	BIOQUANT Life Science,	https://lifescience.bioquant.com/; Bioquant Software RRID:SCR_016423

**Other**		

Compartmentalized microfluidic chambers	This paper	N/A

### Contact for Reagent and Resource Sharing

Further information and requests for resources and reagents should be directed to and will be fulfilled by the Lead Contact, Don W. Cleveland (dcleveland@uscd.edu).

### Experimental Model and Subject Details

#### Mice

##### Generation of hgFUS BAC transgenic mice

The human BAC construct expressing the full length *FUS* gene (18.2Kb) was obtained using DNA recombineering from the RP11-157F22 BAC clone. The sequences of the 5′ and 3′ homology arms used to retrieve the human *FUS* gene were: CCCCATAGCTGGGCAAATTTAGGCCAACACTC and CTAAGCGTGGTGGCGGGCGCCTGTAG. The ALS-linked mutations (R521C and R521H) were introduced using site-specific mutagenesis. Each construct was flanked by LoxP sites to further allow Cre-mediated excision of the human *FUS* gene in cell of interest. No other gene is on the BAC. The modified constructs (FUS wild-type or harboring either the R521H or R521C mutation) was injected into the pronuclei of fertilized C57BL/6 eggs and implanted into pseudo-pregnant female mice. All the mice used in this report were maintained on a pure C57BL/6 background. All experimental procedures were approved by the Institutional Animal Care and Use Committee of the University of California, San Diego.

#### Primary cultures

##### Primary hippocampal neuron cultures

Primary hippocampal neurons were prepared from embryonic day 16-17 (E16-E17). Hippocampal tissues were treated with 0.25% of Trysin+EDTA for 15 min at 37°C. Trypsin activity was inhibited using DMEM:F12 media supplemented with 10% of FBS and 1% of Penicillin/Streptomycin and tissues were then mechanically disaggregated using 1000 μL tips. A 4% BSA (in PBS1x) solution was further added to the cell suspension and centrifuged for 10 min at 1000rpm. The cell pellet was resuspended in Neurobasal media supplemented with 2% of B27, 200mM of L-glutamine and 100U/mL of penicillin-streptomycin. Cells were seeded in culture plates previously coated with poly-D-lysine at the concentration of 600,000 cells/mL.

### Method Details

#### Immunofluorescence

Mice were perfused intracardially and fixed with 4% paraformaldehyde in 0.1 M Sorenson’s phosphate buffer, pH 7.2, the entire spinal cord was dissected, post-fixed for 2 hours in the same fixative and transferred in a 30% sucrose phosphate buffer for at least 2 days. The lumbar spinal cord or brain was embedded in OCT compound (Sakura) and snap frozen in isopentane (2-methylbutane) cooled at −40°C on dry ice. Floating lumbar spinal cord or brain cryosections (30μm or 35μm, respectively) were incubated in a blocking solution containing PBS1x, 0.5% Tween-20, 1.5% BSA for 1.5 hours at room temperature and then in PBS1x, 0.3% Triton X-100 overnight at room temperature with the primary antibodies (listed in antibodies section). Primary antibodies were washed with PBS1x and then detected using donkey anti-rabbit or anti-mouse FITC or Cy3 (1:500) coupled secondary antibodies (Jackson ImmunoResearch). The sections were washed with PBS1x and mounted. Analysis was performed on a Nikon Eclipse laser scanning confocal microscope. Fluorescence intensity from unsaturated images captured with identical confocal settings (minimum of four spinal sections were imaged per animal) was quantified using NIS elements software (Nikon).

#### Morphometric Analysis of Axons

Mice were perfused intracardially and fixed with 4% paraformaldehyde in 0.1 M Sorenson’s phosphate buffer, pH 7.2, and the L5 lumbar roots were dissected and conserved in the same fixative at 4°C. L5 roots were embedded in Epon-Araldite as described in the electron microscopy section, thick sections (0.75μm) were prepared and stained for light microscopy with toluidine blue. Cross sections of L5 motor axons were analyzed at each age group. Axonal diameters were measured using the Bioquant Software and the number of large caliber axons with diameters over 4.5μm (α motor axons) was determined.

#### Motor neuron counting

ChAT positive ventral horn motor neurons were counted from 25-35 lumbar spinal cord cryosections (per animal) spaced 360μm apart and expressed as the average of total motor neurons counted divided by the number of sections.

#### Neuromuscular junction innervation

Gastrocnemius muscle was dissected from perfused mice and prepared as described in the immunofluorescence section. Floating 40 μm thick longitudinal sections of gastrocnemius were incubated in a blocking solution containing PBS1x, 0.5% Tween-20, 1.5% BSA for 4 hours at room temperature and then in PBS1x, 0.3% Triton X-100 overnight at room temperature with the polyclonal rabbit anti-synaptophysin antibody at 1:50 (Invitrogen). The sections were washed with PBS1x and then incubated first with donkey anti-rabbit Cy3 (Jackson ImmunoResearch) and α-bungarotoxin-Alexa488 (Invitrogen) at 1:500 for 1 hour at room temperature and then with Fluoromyelin red (Invitrogen) at 1:300 for 30 min. The sections were further washed with PBS1x and mounted. Analysis was performed on a Nikon Eclipse laser scanning confocal microscope. A total of approximately 1,000 neuromuscular junctions were counted from at least 10 sections of gastrocnemius. Individual NMJs were considered as innervated when synaptophysin staining covered at least 50% of the area of α-bungarotoxin staining.

#### *In vivo* protein synthesis labeling

Intraperitoneal injection of puromycin (10μg/kg) was performed in 12-month-old mice. Exactly 30 min post-injection (Goodman et al., 2011, Khoutorsky et al., 2015), mice were deeply anesthetized and perfused and fixed with 4% paraformaldehyde in 0.1 M Sorenson’s phosphate buffer, pH 7.2. Sciatic nerves were dissected and post-fixed for 3 hours at 4°C, transferred in a 30% sucrose phosphate buffer and crypreserved. 30 μm thick longitudinal and transversal sections of sciatic nerves were processed for immunofluorescence, as described above.

#### Immunoblotting

Spinal cords from Non-Tg or hg*FUS* mice were homogenized in cold PBS1x supplemented with protease inhibitors. The lysates were centrifuged for 10 min at 1,000 x g and the resulting supernatants (clarified tissue extract) were analyzed by immunoblotting. Equal protein amounts were separated on SDS-PAGE, transferred to nitrocellulose membranes and probed with the indicated antibodies followed by horseradish peroxidase-conjugated secondary antibodies (Jackson ImmunoResearch). Pico or Femto ECL (Pierce) was used to detect immunoreactive bands.

#### Antibodies

Rabbit anti-phosphorylated eIF2α (Ser51) (1:100; Cell Signaling; #9721S); Rabbit anti-eiF2a (1:1000; Cell Signaling; #9722S); Mouse anti-FUS (4H11) (1:1000; Santa Cruz, sc-47711); Rabbit anti-FUS (1:500; Bethyl, A300-302A); Goat anti-FUS (1:500; Bethyl, A303-839A); Rabbit anti-FUS (1:500; Proteintech 11570-1-AP); Mouse anti-GAPDH (6C5) (1:10,000; Abcam, ab8245); Mouse anti-GFAP (clone G-A-5) (1:500; Millipore, MAB360); Rabbit anti-HSF1 (1:100; Cell Signaling, 4356S); Rabbit anti-Hsp90 (C45G5) (1:1000; Cell Signaling, 4877S); Rabbit anti-Iba1 (1:500; Wako, 019-19741); Chicken anti-Map2 (1:500; Novus Biological; NB300-213); Mouse anti-Neurofilament Clone RT97 (1:500; Millipore, MAB5262); Rabbit anti-neurofilament H (1:500; Millipore, ab1989); Mouse anti-Neurofilament H non-phosphorylated SMI-32 (1:500; Covance, SMI32R); Mouse anti-Nucleophosmin (1:1000; Zymed, 32-5200); Chicken Neurofilament H (1:1000, Millipore, AB5539); Chicken anti-NeuN (1:1,000, Millipore, ABN91); Mouse anti-puromycin (1:100; Millipore, MABE343); Conjugated Alexa 488 anti-puromycin (1:100; Millipore, MABE343-AF488); Rabbit anti-synapsin-1 (1:500 IF; 1:1000 WB; SYSY synaptic systems, 106103); Mouse anti-γH2A.X (1:5000; Millipore 05-636); Rabbit anti-53BP1 (1:5000; Novus Biological NB100-304); Rabbit anti-calnexin (1:1,000; Enzo, SPA-860); Mouse anti-tubulin (DM1A clone) (1:10,000, home-made); Rabbit anti-human FUS #14080 (1:25000; home-made) and anti-mouse FUS #14082 (1:4000, home-made).

#### Nuclear-Cytosolic Fractionation

Spinal cords were dissected, weighed, and fresh tissue was gently lysed in 10x (vol/wt) hypotonic buffer A (10 mM HEPES-KOH pH 7.4, 10 mM KCl, 1.5 mM MgCl, 0.5 mM EDTA, 0.5 mM EGTA), 1x protease inhibitors (Roche) using a pre-chilled glass dounce homogenizer (tight fit). After 15 min on ice, 2.5 M sucrose [0.5x (vol/wt)] was added and samples were centrifuged at 800 x g for 5 min. The supernatant was collected as the cytosolic fraction, and the nuclear pellet was washed with buffer A. Following centrifugation, the nuclear pellet was resuspended in 5x (vol/wt) buffer B [10 mM HEPES-KOH pH 7.4, 0.42 M NaCl, 2.5% (vol/vol) glycerol, 1.5 mM MgCl, 0.5 mM EDTA, 0.5 mM EGTA, 1 mM DTT, 1x protease inhibitors], and incubated at 4°C while rotating at 60 rpm for 1 hour. Both the nuclear and cytosolic fractions were then centrifuged at 16,100 x g for 10 min at 4° C.

#### Sequential Biochemical Fractionation

Spinal cords from mice were dissected, weighed, and homogenized in 4 mL/g of high-salt buffer (HS buffer: 50 mM Tris pH 7.5, 750 mM NaCl, 5 mM EDTA, and protease inhibitor mixture), and then centrifuged at 45,000 x g for 30 min at 4° C. The pellets were extracted with HS containing 1% (wt/vol) Triton X-100 (TX fractions). Pellets were homogenized in 500 μL of HS buffer containing 1% (wt/vol) Triton X-100 and 1 M sucrose, and upon centrifugation, floating myelin was removed. Pellets were then extracted with 2 mL/g of urea buffer (7 M urea, 2 M thiourea, 4% (wt/vol) CHAPS, 30 mM Tris pH 8.5), followed by 2 mL/g of SDS loading buffer. Equivalent volumes of samples were separated on 10% Bis-Tris gels for immunoblotting with the indicated antibodies.

#### Total-body mouse γ-irradiation

To obtain a positive control for DNA damage accumulation in mouse spinal cords, Non-Tg mice (n = 3) were irradiated for 10 min at 10Gy of γ-rays. Animals were perfused within 2 hours after irradiation and processed for immunofluorescence staining using anti-γH2A.X and −53BP1 antibodies as previously described (Ahmed et al., 2017, Koch et al., 2016).

#### Cell viability assay

The viability of the neurons was tested using LIVE/DEAD Reduced Biohazard Viability/Cytotoxicity Kit (Molecular Probes). Neurons grown in 96 well plates were incubated with the kit solution mix for 15 min in complete darkness at room temperature. The cells were then rinsed with HBSS and fixed with freshly prepared 4% of glutaraldehyde for 15 min at room temperature before imaging by confocal microscopy.

#### Multi-electrode array (MEA)

Each well of a 12-well MEA plate from Axion Biosystems was coated with poly-D-lysine prior to cell seeding. Hippocampal neurons from Non-Tg and hg*FUS*^R521H^ mice embryos were plated at the same density (40,000 neurons per well), with duplicate wells for each embryo. Cells were fed every 3-4 days and measurements were taken before the medium was changed. Recordings were performed using a Maestro MEA system and AxIS software (Axion Biosystems), using a band-pass filter with 0.1 Hz and 5 kHz frequency cutoffs. Spike detection was performed using the neural Spikes analog setting (1200XGain, 200-5000Hz) and an adaptive threshold set to 5.5 times the standard deviation of the estimated noise on each electrode. Each plate first rested for 5 min in the Maestro, and then 5 min of data were recorded to calculate the spike rate per well. MEA analysis was performed using the Axion Biosystems Neural Metrics Tool, wherein electrodes that detected at least 5 spikes/minute were classified as active electrodes. Bursts were identified in the data recorded from each individual electrode using an adaptive threshold algorithm. Network bursts were identified for each well requiring a minimum of ten spikes with a maximum inter-spike of 100 ms. Only channels that exhibited bursting activity (more than 10 spikes in 5-min interval) were included in the analysis.

#### Compartmentalized microfluidic devices

The microfluidic devices were prepared as previously described (Niederst et al., 2015). Master molds were fabricated by photolithography, by the Bioengineer Department of the University of California, San Diego, Nano3 Cleanroom Facility. Devices were molded by soft lithography using Sylgard 182 (Ellsworth Adhesives, Germantown, WI) as previously described (Taylor et al., 2005). After cured, the cut devices where bath-sonicated in water, washed in 70% ethanol and dried under the hood before mounted onto glass coverslips. The devices were coated with 1X poly-D-lysine 2 hours at 37°C and washed sequentially in water and PBS1x before plating the cells.

#### Puromycin incorporation assay

Primary hippocampal neurons after 5 or 15 days of culture were incubated for 10 min with puromycin at 1μg/mL. After the incubation cells were washed with PBS1x and fixed with 4% of paraformaldehyde, permeabilized and stained with the anti-puromycin antibody (1:100) for 1 hour at room temperature. Fluorescence was visualized using an Olympus confocal microscope and images were acquired using a 60X objective.

#### [^35^S]-Methionine Labeling/Radioactivity assay

Primary hippocampal neurons were cultivated for 15 days. Medium in each well was exchanged to Met-/Cys- medium for 25 min before being supplemented with 1mCi/mL [35S]-Met media (for a final dosage of 0.1mCi/mL). [S35]-Met media containing 25μg/mL cycloheximide was used as negative control for radioactivity incorporation. After 2 hours of incubation with [35S]-Met at 0.1mCi/mL, incorporation was stopped with cycloheximide (25μg/mL) and cells collected immediately in ice-cold PBS1X. Cells were lysed with RIPA buffer (50mM Tris pH8.0, 50mM NaCl, 5mM EDTA, 1% NP-40, 0.5% sodium deoxycholate, 0.1% SDS) containing proteinase inhibitors and total cellular protein was co-precipitated with 100μg of BSA carrier protein using trichloroacetic acid. Samples were acetone washed, resuspended in 2% SDS-PBS1X, and transferred into scintillation fluid. Counts per minute (Cpms) averaged over 5-min windows were measured with a Beckman LS6000 SC scintillation counter (normalized to scintillation fluid only). Cpms were then normalized to total well protein concentration determined by densitometry from a silver-stained gel.

#### RNA extraction and qPCR

To isolate total RNA from cells or tissues, TRIzol (Invitrogen) and treatment with RQ1 DNase I (Promega) was used. Reverse transcription was performed using the SuperScript First Strand Kit (Invitrogen) according to manufacturer’s instructions. qPCR was performed on 40ng of cDNA using the iQSYBR Green Supermix (Bio-Rad) with the iCycler iQ detection system according to manufacturer’s instructions. Analysis was performed using the iQ5 optical system software (Bio-Rad; version 2.1). Expression values were expressed as a percentage of the average expression of control samples. All reactions were carried out in duplicate in three independent mice (per genotype) and *actin B* or *cyclophilin* and/or *rsp9* genes were also measured as standard genes across all experimental conditions. To enable determining human *FUS* RNA levels compared to mouse *Fus*, primer sequences common to both human and mouse were used to measure total *FUS* RNAs. In parallel, primer sequences specific to mouse *Fus* were designed to measure uniquely mouse *Fus* RNAs. Human *FUS* (compared to endogenous) is then obtained by subtracting mouse *Fus* from total *FUS* levels. Real-time quantitative RT-PCR (RT/qPCR) was performed to determine mRNA levels in spinal cord or hippocampal neurons from transgenic mice (see primer sequence in Table S2).

#### RNA-seq

Total RNA from spinal cords of 18-month-old m*Fus*^−/−^/hg*FUS* (WT, R521C or R521H) and their Non-Tg control littermates were extracted with TRIzol (Invitrogen). RNA quality was measured using the Agilent Bioanalyzer system according to the manufacturer’s recommendations and processed using the Illumina TruSeq Stranded mRNA Sample Preparation Kit according to manufacturer’s protocol. A total of 14 cDNA libraries were simultaneously generated and sequenced using an Illumina HiSeq 2000 sequence, as previously described (Scekic-Zahirovic et al., 2016), before fastq files were obtained from Illumina demultiplexing *“*bcl2fastq*”*. Then fastq files were aligned to a mouse reference genome (mm9, UCSC Genome Browser) using TopHat (Trapnell et al., 2009) and the transcript abundance for each annotated protein-coding gene as fragments per kilobase of transcript per million mapped reads (FPKM) were estimated by Cufflinks (Trapnell et al., 2010). Sequencing yielded, on average, 15 million non-redundant reads per sample. 13,468 genes, which expressed FPKM > = 1 were kept for downstream analyses. The spearman correlation among samples as well as Principle Component Analysis (PCA) was applied to those *12,922* genes (expressing consistently within the same category). The proportion of variance for dimension 1 (PC1) and dimension 2 (PC2) shown in Figure 5B was of 0.9 and 0.1, respectively. Cuffdiff (Trapnell et al., 2013), a part of the Cufflinks package, was used to detect the differentially expressed genes (DEGs). Unsupervised hierarchical clustering with complete method was applied on the heatmap showing DEGs between wild-type and mutant samples. Gene ontology of those DEGs was estimated by David and Ingenuity Pathway Analysis (IPA). All sequencing raw data were submitted to Gene Expression Omnibus (GEO) with accession number GSE120247.

#### RASL-seq

RASL-seq analysis of splicing switches was carried out as detailed (Scekic-Zahirovic et al., 2016, Sun et al., 2015, Zhou et al., 2012). A pool of oligonucleotides was designed to detect 5,859 alternative splicing events. One hundred fmol of RASL-seq oligos were annealed to 1 μg of total RNA isolated from mouse spinal cords. After ligation, 5 μl eluted ligated oligos was used for 16∼20 cycles of PCR amplification, and the bar-coded PCR products were sequenced on HiSeq2000 with 24-30 samples in one lane. Sequencing data were decoded allowing no mismatch with each barcode, and target sequences were mapped with RASL-seq oligo pool sequences. Ratios of the counts of shorter to longer isoforms were calculated.

#### Splicing gels

Semiquantitative qRT-PCR (25-30 cycles) was used to assess splicing changes most affected upon FUS depletion identified by (Lagier-Tourenne et al., 2012). Isoform products were separated on 10% polyacrylamide gels and stained with SYBR gold (Invitrogen) and quantified with ImageJ software to record the intensity of the bands corresponding to different splicing isoforms.

#### Mouse behavioral tests

##### Grip strength

Grip strength was measured using a Grip Strength Meter (Columbus Instruments, Columbus, OH) on cohorts (n ≥ 15) made up of approximately the same number of males and females. Mice were allowed to grip a triangular bar only with hind limbs, followed by pulling the mice until they released; five force measurements were recorded in each separate trial.

##### Y maze test

Spontaneous alternation behavior, a measure of spatial working memory, exploratory behavior, and responsiveness to novelty (Lalonde, 2002), was tested using a Y maze with 34 × 8 × 14-cm arms. Each mouse was tested in a single 5-min trial in which arm choices and total numbers of arm entries were recorded. Spontaneous alternation, expressed as a percent, refers to the ratio of sets of three unique arm choices (i.e., visiting arm 3 then 1 then 2 in sequence) to the total number of arm entries. Because mice have the opportunity to do repeated entries into a single arm, there is a chance performance level of 22% (2/9) for spontaneous alternations (Holcomb et al., 1999, Pennanen et al., 2006). In our hands, healthy young C57BL6/J mice typically make 50%–70% spontaneous alternations in this test. A cohort of n = 15 animals (per genotype) was assessed.

##### Social interaction test

This test was originally developed to model in mice aspects of autism spectrum disorders in humans (Moy et al., 2004, Moy et al., 2007) and has been used widely by behavioral neuroscientists (Silverman et al., 2010). Individuals on the autism spectrum show aberrant reciprocal social interaction, including low levels of social approach and unusual modes of interaction. The social interaction apparatus is a rectangular, three chambered Plexiglas box, with each chamber measuring 20 cm x 40.5 cm x 22 cm (L x W x H). Dividing walls are clear with small semicircular openings (3.5 cm radius) allowing access into each chamber. The middle chamber is empty, and the two outer chambers contain small, round wire cages (Galaxy Cup, Spectrum Diversified Designs, Inc., Streetsboro, OH) during testing. The mice were habituated to the entire apparatus with the round wire cages removed for 5 min. To assess sociability, mice were returned to the middle chamber, this time with a stranger mouse (B6 of the same sex being tested, habituated to the wire cage) in one of the wire cages in an outer compartment and another identical wire cage in the opposite compartment. Time spent in the chamber with the stranger mouse and time spent in the chamber with the novel object was recorded for 5 min. For the social novelty preference test, mice were returned to the middle chamber, this time with the original mouse (familiar mouse) in its chamber and a new unfamiliar mouse (novel mouse) in the previously empty wire cage. Again, time spent in each chamber was recorded for 5 min. Young male B6 mice spend more time with the novel mouse in the sociability test (Moy et al., 2004), however we have found less preference for the novel mouse as compared to the now familiar mouse in the social novelty preference test (Jackson et al., 2015). A cohort of n = 15 animals (per genotype) was assessed.

##### Novel object recognition test

This test assays recognition memory while leaving the spatial location of the objects intact and is believed to involve the hippocampus, perirhinal cortex, and raphe nuclei (Lieben et al., 2006, Mumby et al., 2005, Winters et al., 2004). The basic principal is that animals explore novel environments and that with repeated exposure decreased exploration ensues (i.e., habituation). A subsequent object substitution (replacing a familiar object with a novel object) results in dishabituation of the previously habituated exploratory behavior (Ennaceur and Delacour, 1988). The resulting dishabituation is expressed as a preferential exploration of the novel object relative to familiar features in the environment. This dishabituation has generally been interpreted as an expression of the animal’s recognition memory: the novel object is explored preferentially because it differs from what the animal remembers (Heyser and Chemero, 2012) and requires attention by the animals. Mice were individually habituated to a 51cm x 51cm x 39cm open field for 5 min and then tested with two identical objects placed in the field (either two 250 mL amber bottles or two clear plastic cylinders 6x6x16cm half filled with glass marbles). Each mouse was allowed to explore the objects for 5 min. After three such trials (each separated by 1 min in a holding cage), the mouse was tested in the object novelty recognition test in which a novel object replaced one of the familiar objects (for example, an amber bottle if the cylinders were initially used). All objects and the arena were thoroughly cleaned with 70% ethanol between trials to remove odors. Behavior was video recorded and then scored for contacts (touching with nose or nose pointing at object and within 0.5 cm of object). Habituation to the objects across the familiarization trials (decreased contacts) is an initial measure of learning and then renewed interest (increased contacts) in the new object indicated successful object memory. Recognition indexes were calculated using the following formula: # contacts during test/(# contacts in last familiarization trial + # contacts during test). Values greater than 0.5 indicate increased interest, whereas values less than 0.5 indicate decreased interest in the object during the test relative to the final familiarization trial. A cohort of n = 15 animals (per genotype) was assessed.

##### Cued and contextual fear conditioning

In this procedure, mice learn to associate a novel environment (context) and previously neutral stimuli (conditioned stimuli, a tone and light) with an aversive foot shock stimulus (Maren, 2001). It allows for the assessment of both hippocampus-dependent and amygdala-dependent learning processes in the same mouse (Kenney and Gould, 2008, Rudy et al., 2004). Testing then occurs in the absence of the aversive stimulus. Conditioned animals, when exposed to the conditioned stimuli, tend to refrain from all but respiratory movements by freezing. Freezing responses can be triggered by exposure to either the context in which the shock was received (context test) or the conditioned stimulus (CS+ test). Conditioning took place in Freeze Monitor chambers (Med Associates, Inc.) housed in sound proofed boxes. The conditioning chambers (26 × 26 × 17 cm) were made of Plexiglas with speakers and lights mounted on two opposite walls and shockable grid floors.

On day 1, mice were placed in the conditioning chamber for five minutes in order to habituate them to the apparatus. On day 2, the mice were exposed to the context and conditioned stimulus (30 s, 3000 Hz, 80 dB sound + white light) in association with foot shock (0.60 mA, 2 s, scrambled current). Specifically, the mice received 3 shock exposures in their 6 min test, each in the last 2 s of a 30 s tone/light exposure. On day 3, contextual conditioning (as determined by freezing behavior) was measured in a 5-min test in the chamber where the mice were trained (context test). On the following day, the mice were tested for cued conditioning (CS+ test). The mice were placed in a novel context for 3 min, after which they were exposed to the conditioned stimuli (light + tone) for 3 min. For this test, the chamber was disguised with new walls (white opaque plastic creating a circular compartment in contrast to a clear plastic square compartment) and a new floor (white opaque plastic in contrast to metal grid). Freezing behavior (i.e., the absence of all voluntary movements except breathing) was measured in all of the sessions by real-time digital video recordings calibrated to distinguish between subtle movements, such as whisker twitches, tail flicks, and freezing behavior. Freezing behavior in the context and cued tests (relative to the same context prior to shock and an altered context prior to tone, respectively) is indicative of the formation of an association between the particular stimulus (either the environment or the tone) and the shock; i.e., that learning had occurred. A cohort of n = 15 animals (per genotype) was assessed.

### Quantification and Statistical Analysis

The number of independent repeats (n), the statistical test used for comparison and the statistical significance (p values) are specified for each figure panel in the representative figure legend. Data are presented as mean ± SEM. Data were analyzed and graphs were generated using GraphPad Analysis software.

### Data and Software Availability

RNA sequencing data is submitted to Gene Expression Omnibus (GEO) with accession number GSE120247.

## Supplemental Information

Document S1. Figures S1–S6 and Table S2

Table S1. List of Genes with Altered Expression in Spinal Cords of Mutant FUS Mice; Related to Figure 5C

Document S2. Article plus Supplemental Information
