# ALS/FTD-Linked Mutation in FUS Suppresses Intra-axonal Protein Synthesis and Drives Disease Without Nuclear Loss-of-Function of FUS

**DOI:** 10.1016/j.neuron.2020.04.006

**Published:** 2020-04-22

**Authors:** Jone López-Erauskin, Takahiro Tadokoro, Michael W. Baughn, Brian Myers, Melissa McAlonis-Downes, Carlos Chillon-Marinas, Joshua N. Asiaban, Jonathan Artates, Anh T. Bui, Anne P. Vetto, Sandra K. Lee, Ai Vy Le, Ying Sun, Mélanie Jambeau, Jihane Boubaker, Deborah Swing, Jinsong Qiu, Geoffrey G. Hicks, Zhengyu Ouyang, Xiang-Dong Fu, Lino Tessarollo, Shuo-Chien Ling, Philippe A. Parone, Christopher E. Shaw, Martin Marsala, Clotilde Lagier-Tourenne, Don W. Cleveland, Sandrine Da Cruz

(Neuron *100*, 816–830.e1–e7; November 21, 2018)

In the original publication of this paper, the concentration of the puromycin was incorrectly reported. In both panel A of Figure 7 and the “In vivo protein synthesis labeling” section of the STAR Methods, the amount was listed as 10 μg/kg. The correct concentration should be 10 mg/kg. The authors apologize for this error.

