# Supplementary material for: ALS/FTD-Linked Mutation in FUS Suppresses Intra-axonal Protein Synthesis and Drives Disease Without Nuclear Loss-of-Function of FUS
Source: Neuron. 2018 Nov 21;100(4):816–830.e7. doi: 10.1016/j.neuron.2018.09.044 (PMC6277851; doi:10.1016/j.neuron.2018.09.044)
Supplement: Document S1. Figures S1–S6 and Table S2 [file mmc1.pdf]

Neuron, Volume *100*

Supplemental Information

ALS/FTD-Linked Mutation in FUS Suppresses Intra-axonal Protein Synthesis and Drives Disease Without Nuclear Loss-of-Function of FUS

Jone López-Erauskin, Takahiro Tadokoro, Michael W. Baughn, Brian Myers, Melissa McAlonis-Downes, Carlos Chillon-Marinas, Joshua N. Asiaban, Jonathan Artates, Anh T. Bui, Anne P. Vetto, Sandra K. Lee, Ai Vy Le, Ying Sun, Mélanie Jambeau, Jihane Boubaker, Deborah Swing, Jinsong Qiu, Geoffrey G. Hicks, Zhengyu Ouyang, Xiang-Dong Fu, Lino Tessarollo, Shuo-Chien Ling, Philippe A. Parone, Christopher E. Shaw, Martin Marsala, Clotilde Lagier-Tourenne, Don W. Cleveland, and Sandrine Da Cruz

A

Transgene	Strain	# founder lines	# established lines
hgTLS^{WT}	C57BL/6	19	4
hgTLS^{R521C}	C57BL/6	2	1
hgTLS^{R521H}	C57BL/6	5	3

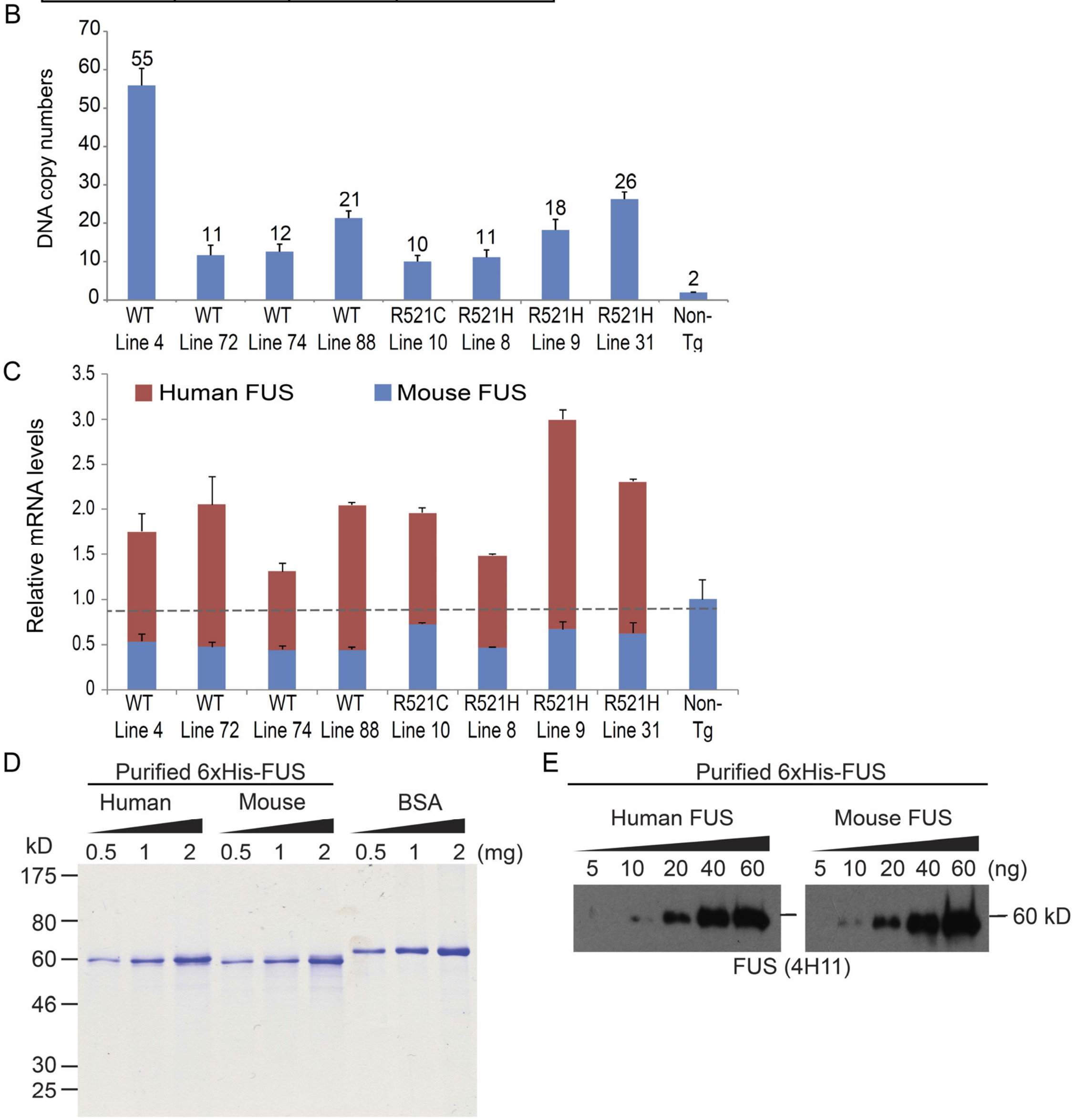

Figure S1: Expression levels of the human *FUS* transgene mimics that of endogenous protein with an auto-regulation mechanism. (Related to Figure 1) (A) Summary of the hg*FUS* transgenic lines per genotype that were generated. (B) DNA copy numbers found in tails of Non-Tg, hg*FUS*^{WT}, hg*FUS*^{R521C} and hg*FUS*^{R521H} mice by qPCR (n=3 mice per line). The bar graph represents mean ± SEM. (C) Expression levels of total (mouse and human) *FUS* RNAs in cortex from 2-month-old Non-Tg, hg*FUS*^{WT}, hg*FUS*^{R521C} and hg*FUS*^{R521H} mice measured by qRT-PCR, normalized to the level of endogenous *Fus* RNA (n=3 mice/line). The bar graph represents mean ± SEM. (D) Purified his-tagged full length human and mouse FUS recombinant proteins analyzed by Coomassie staining of a SDS-polyacrylamide gel. (E) Immunoblotting using FUS antibody (4H11) to determine its affinity for human and mouse FUS proteins. The right and left immunoblot panels with recombinant mouse and human FUS, respectively belong to the same original blot, which was digitally cut as non-relevant samples were loaded in between.

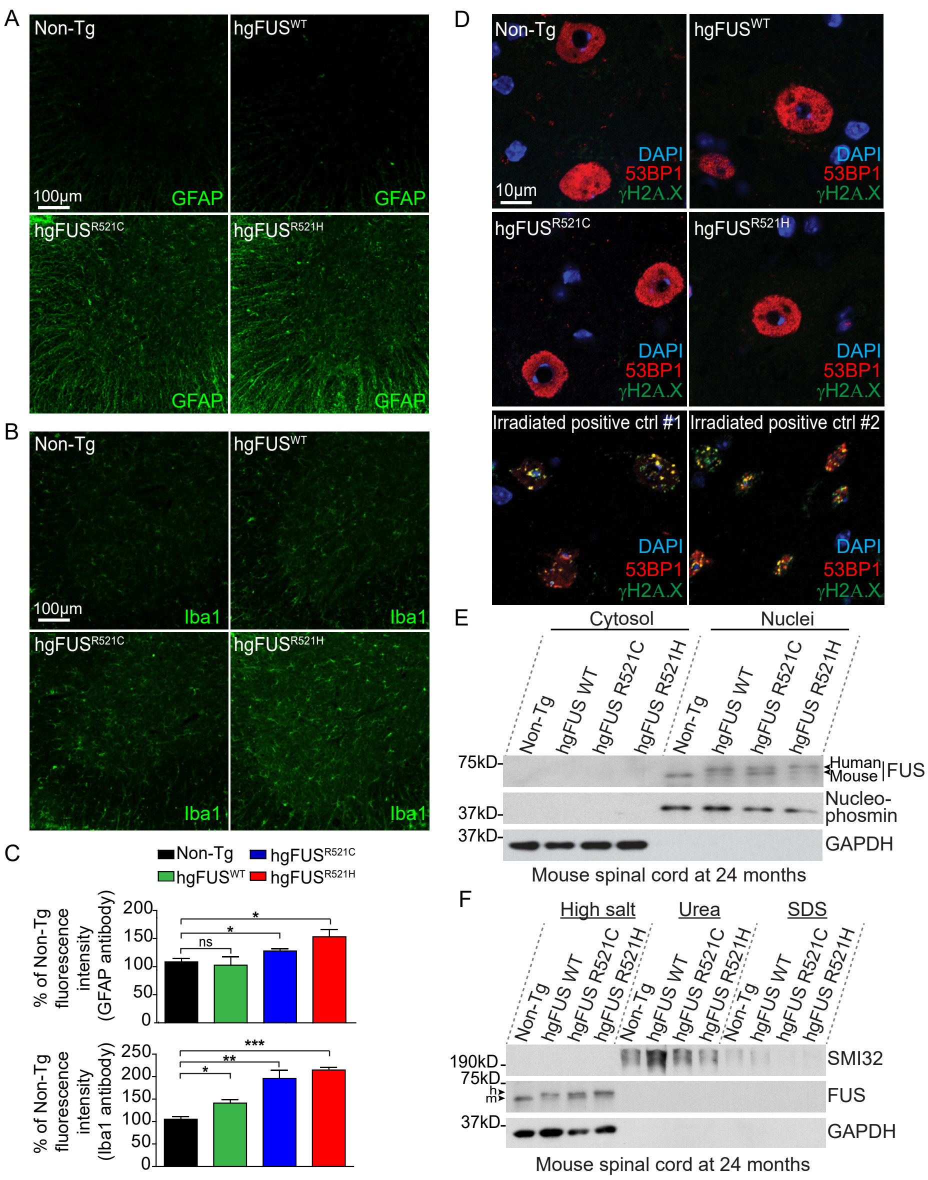

Figure S2: Age-dependent progressive lower motor neuron disease without DNA damage accumulation or cytoplasmic aggregation of FUS in mice expressing ALS-linked mutants of human FUS. (Related to Figure 2) (A-B) Lumbar spinal cord sections from 24-month-old Non-Tg, hg*FUS*^{WT}, hg*FUS*^{R521C} and hg*FUS*^{R521H} mice processed for immunofluorescence using an antibody detecting activated (A) astrocytes (GFAP) or (B) microglia (Iba1). Scale bar, 100 µm. (C) Quantification of the relative fluorescence intensity of the GFAP and Iba1 staining of lumbar spinal cord sections from 24-month-old Non-Tg, hg*FUS*^{WT}, hg*FUS*^{R521C} and hg*FUS*^{R521H} mice. The bar graph represents mean ± SEM from n=3 animals per genotype. * $p<0.05$, ** $p<0.01$, *** $p<0.001$, two-sided unpaired Student's t-test. (D) Lumbar spinal cord sections from 24-month-old Non-Tg, hg*FUS*^{WT}, hg*FUS*^{R521C} and hg*FUS*^{R521H} mice processed for immunofluorescence using 53BP1 (red) and γH2A.X (green) antibodies staining for DNA damage as revealed by co-localized aggregation in the positive control irradiated mice. DNA is stained with DAPI. Scale bar, 10 µm. (E-F) Immunoblot demonstrating (E) lack of cytoplasmic redistribution or (F) major aggregation of FUS using an antibody recognizing both species with similar affinity. (E) Enrichment for nuclear and cytosolic proteins was confirmed using Nucleophosmin and GAPDH antibodies, respectively. (F) SMI32 (neurofilament) and GAPDH antibodies were used as markers for proteins solubilized in the presence of an urea- or high-salt-containing buffer, respectively. Each lane represents an independent 24-month-old mouse spinal cord.

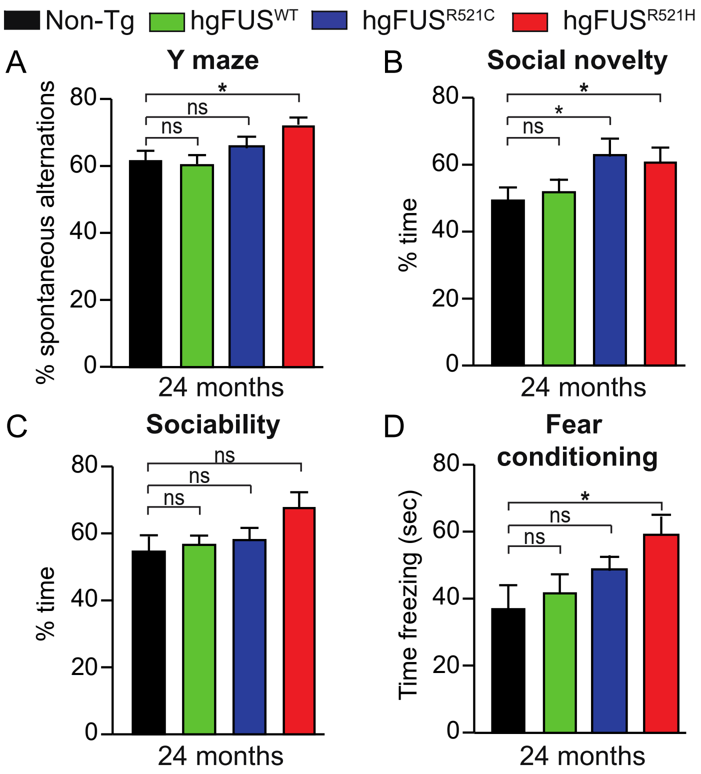

Figure S3: Age-dependent cognitive impairments in mice expressing ALS-linked mutants of human FUS (Related to Figure 3) (A) Spatial memory (Y maze), (B) social novelty, (C) sociability and (D) anxiety (fear conditioning) were measured in 24-month-old Non-Tg, hg*FUS*^{WT}, hg*FUS*^{R521C} and hg*FUS*^{R521H} mice. Data are represented as mean ± SEM from n=15 animals (both genders) per genotype. * $p<0.05$, ns: non-significant using one-way ANOVA.

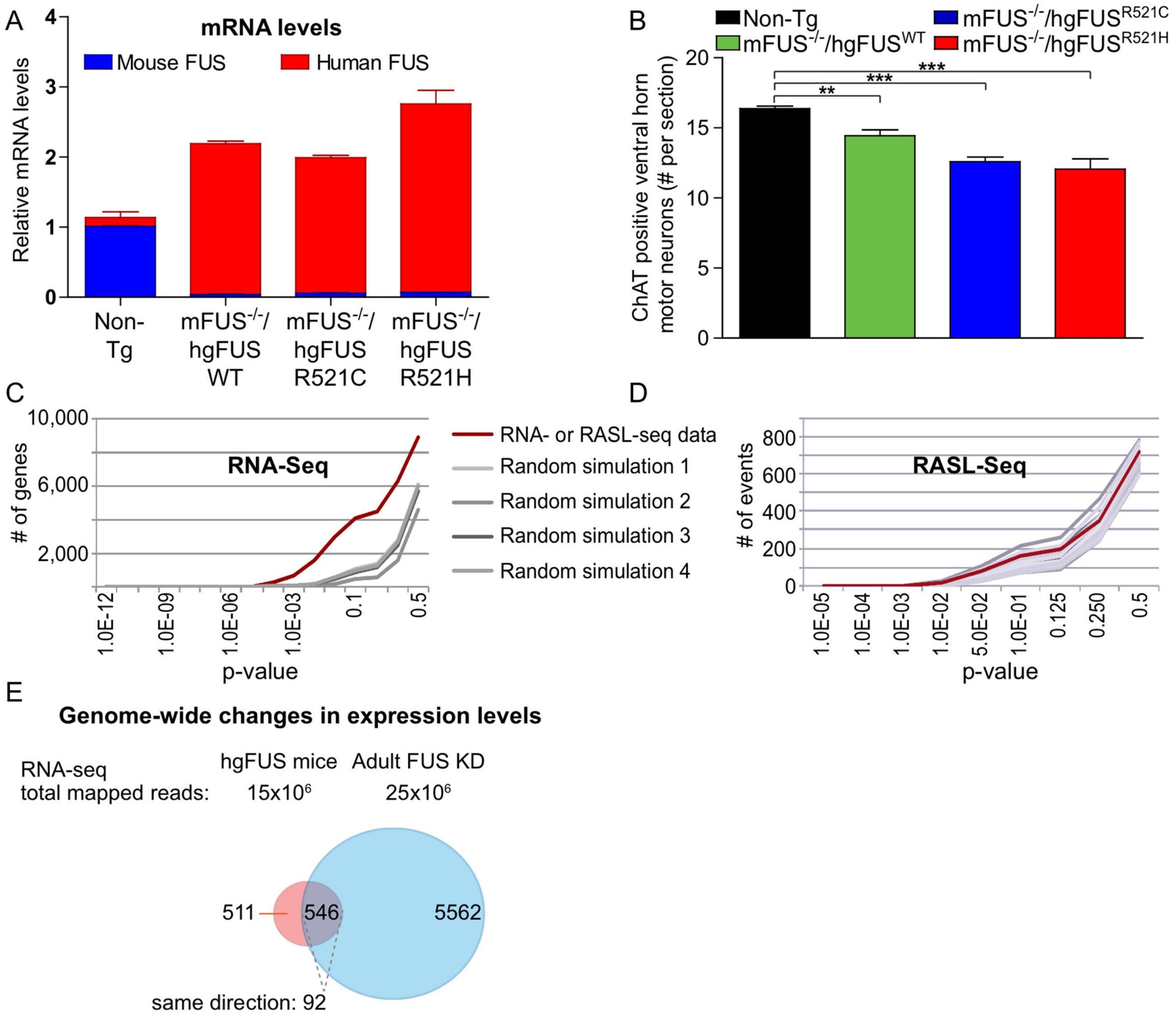

Figure S4: Mutant-dependent RNA signature associated with motor deficits by toxic gain-, but not loss-of-function of ALS-linked mutants of human FUS. (Related to Figures 4-5) (A) Expression levels of total (mouse and human) *FUS* RNAs in spinal cord of Non-Tg, m*Fus*$^{-/-}$/hg*FUS*^{WT}, m*Fus*$^{-/-}$/hg*FUS*^{R521C} and m*Fus*$^{-/-}$/hg*FUS*^{R521H} mice measured by qRT-PCR, normalized to the level of endogenous *FUS* RNA (n=3 mice per line). (B) Quantification of the total number of ChAT positive motor neurons in lumbar spinal cords of 18-month-old Non-Tg, m*Fus*$^{-/-}$/hg*FUS*^{WT}, m*Fus*$^{-/-}$/hg*FUS*^{R521C} and m*Fus*$^{-/-}$/hg*FUS*^{R521H} mice. The bar graph represents mean ± SEM from n≥3 animals per genotype. ** $p<0.01$ and *** $p<0.001$, two-sided unpaired Student's t-test. (C) RNA-seq analysis of all the genes that were differentially expressed in spinal cords from mutants m*Fus*$^{-/-}$/hg*FUS*^{R521C} and m*Fus*$^{-/-}$/hg*FUS*^{R521H} mice (red curve) compared with the dataset where the genotypes were randomly shuffled. (D) RASL-seq analysis of all the splicing events that were identified in spinal cords from mutants m*Fus*$^{-/-}$/hg*FUS*^{R521C} and m*Fus*$^{-/-}$/hg*FUS*^{R521H} mice (red curve) compared with the dataset where the genotypes were randomly shuffled. No significant splicing events were identified that are distinct in the mutant mice compared to randomized splicing events. (E) Venn diagrams showing the overlap of significantly differentially expressed genes in spinal cords from mutant m*Fus*$^{-/-}$/hg*FUS*^{R521C} and m*Fus*$^{-/-}$/hg*FUS*^{R521H} animals compared to adult mouse spinal cords depleted of FUS upon antisense oligonucleotide treatment identified by (Kapeli et al., 2016).

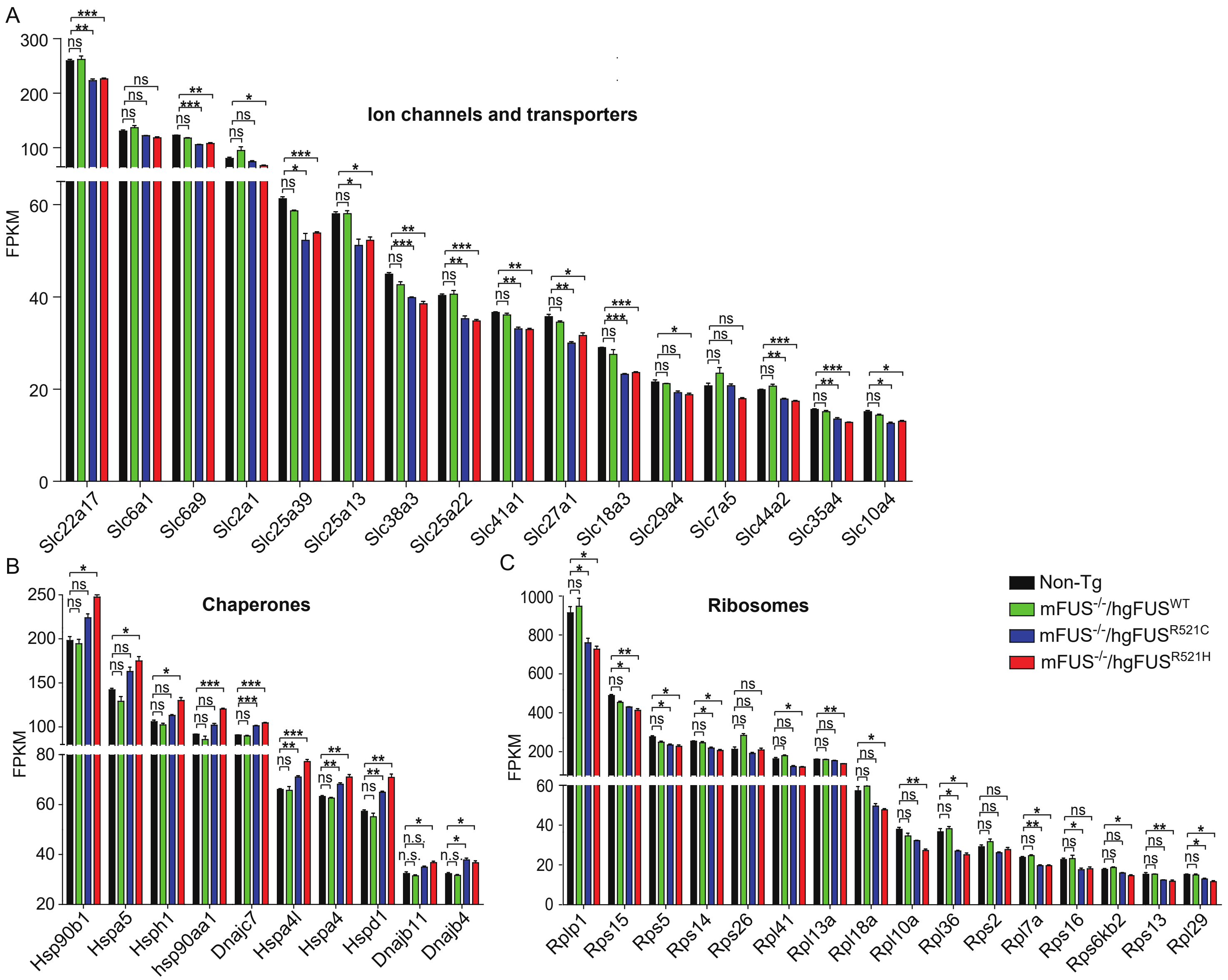

Figure S5: Mutant-dependent RNA signature associated with motor deficits caused by toxic gain-of-function of ALS-linked mutants of human FUS. (Related to Figures 5) (A) Example of expression of ion channels and transporters essential for synaptic function found to be down-regulated by RNA-seq analysis [FPKM (fragments per kilobase of transcript per million mapped reads)] of spinal cord of 18-month-old mutant m*Fus*$^{-/-}$/hg*FUS*^{R521C} and m*Fus*$^{-/-}$/hg*FUS*^{R521H} mice compared to age-matched Non-Tg. Data are represented as mean ± SEM from n≥3 animals per genotype. * $p<0.05$, ** $p<0.01$ and *** $p<0.001$, two-sided unpaired Student's t-test. (B) Example of the chaperone encoded genes found to be up-regulated by RNA-seq analysis [FPKM values] of spinal cord of 18-month-old mutant m*Fus*$^{-/-}$/hg*FUS*^{R521C} and m*Fus*$^{-/-}$/hg*FUS*^{R521H} mice compared to age-matched Non-Tg and m*Fus*$^{-/-}$/hg*FUS*^{WT} animals. Data are represented as mean ± SEM from n≥3 animals per genotype. * $p<0.05$ and ** $p<0.01$, two-sided unpaired Student's t-test. (C) Example of the ribosome genes found to be down-regulated by RNA-seq analysis [FPKM values] of spinal cord of 18-month-old mutant m*Fus*$^{-/-}$/hg*FUS*^{R521C} and m*Fus*$^{-/-}$/hg*FUS*^{R521H} mice compared to age-matched Non-Tg and m*Fus*$^{-/-}$/hg*FUS*^{WT} animals. Data are represented as mean ± SEM from n≥3 animals per genotype. * $p<0.05$ and ** $p<0.01$, two-sided unpaired Student's t-test.

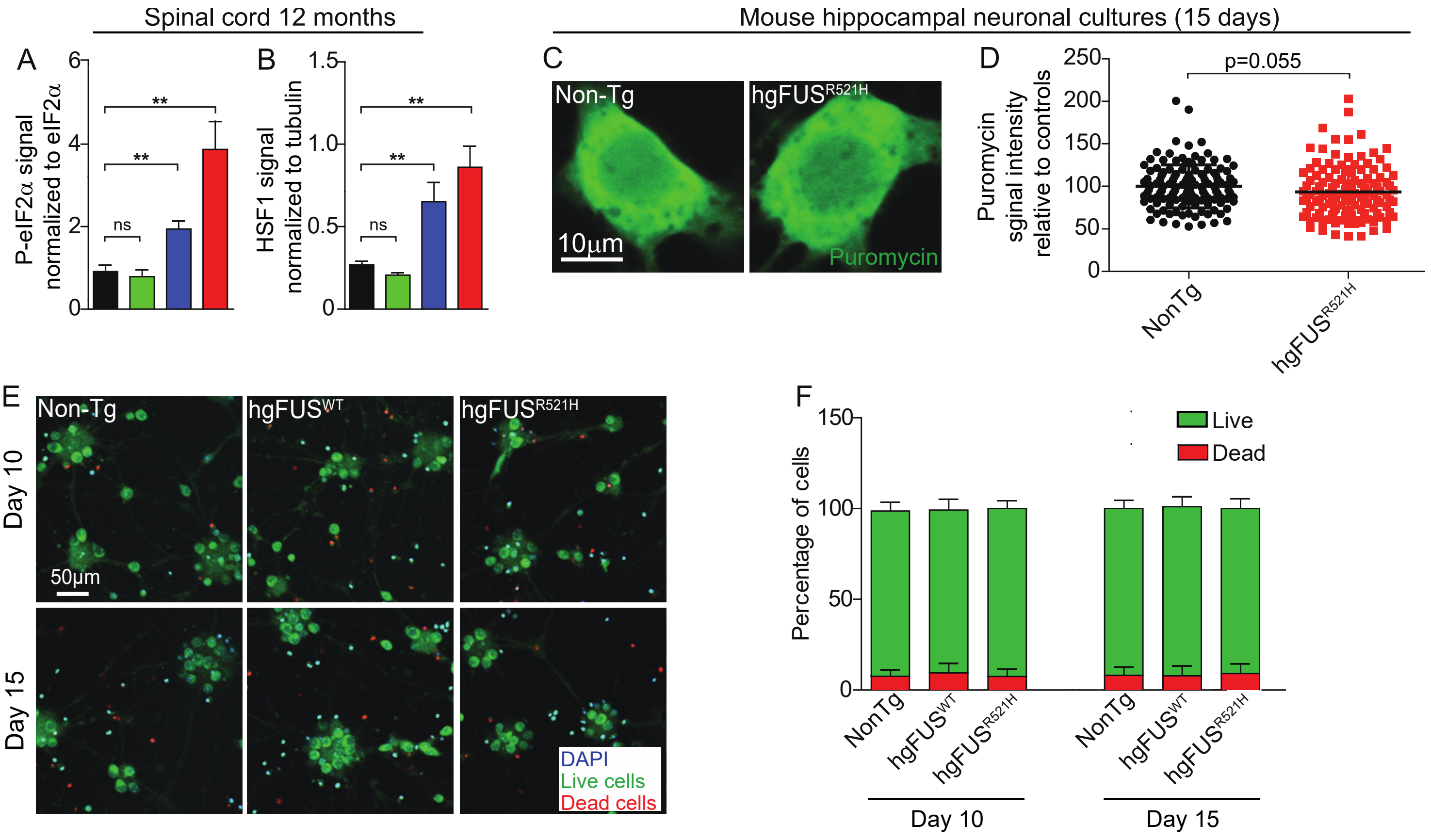

Figure S6: Mutation in FUS causes stress-mediated reduction in axonal protein synthesis (related to Figure 6) (A-B) Quantification of immunoblotting using P-eIF2α and HSF1 antibodies in mouse spinal cords (at 12 months of age), shown in Figure 6A. Data are represented as mean ± SEM from three independent experiments. (C-D) Additional representative images of primary hippocampal cell bodies (cultured for 15 days) stained with antibody against puromycin and quantification of the relative fluorescence intensity of puromycin per cell bodies. Each dot represents the fluorescence intensity per cell body in each genotype. N=130. (E-F) Representative micrograph and quantification of the viability of hippocampal neurons after 10 or 15 days in culture. Live cells are in green, dead cells in red and nuclei in blue.

Table S1: List of genes with altered expression in spinal cords of mutant FUS mice (related to Figure 5C). See individually uploaded Supplemental Table 1 file.

Table S2: Related to Figures 1C, 5F, 5G, 5H, 6B, S1C and S4A. qPCR primers sequences

Gene Name	Forward 5'-3'	Reverse 5'-3'
Mouse FUS	GGCCAGTCAGCTGACACTTCAG	TGCTGCCATAGCCTCCAGTGGAA
Human/Mouse FUS	TCCCAGCAGAGCAGTCAGCCCTA	CCTGGGGAGTTGACTGAGTTCC
Human/Mouse FUS (genomic DNA)	TGGGTTCATGACTTAGGTGTCATTG	CATGTCCCAGGGAACAGACCAG
Slc25a15	CAGGACTCGTCAGGACCTTC	TGCTTCCAATTGGTTCATCA
Slc2a1	GTCCTGCTGCTATTGCTGTG	GAGCACCGTGAAGATGATGA
Scl19a3	CAGGCCTTCAGAGGCATTCT	GGTAGTCGGTGAGGACAAACAC
Slc17a7	AGCATCTTGATGGGCATTTC	CGAAGCAAAGACCCCATAGA
Slc1a3	AGAACCCCTGGGTTTTCATT	GGACAAGTGCTCGACAATCC
Slc39a2	GTCATCACCACAGGGTCCTC	GAAGCAGCATCACGAGAAGA
Slc41a1	ACATGACTGCTGCACTGCTC	ATGGGATGGAGAAGTTGTCG
Slc22a17	CCCACCAGACTTCAACCACT	GCAAAGCCCAAGATGAAGAG
Slc44a2	CGGATCAGAATTGTGCAAGA	CATGCCATAGACGCTGAAGA
Slc10a4	GGCAGTCACCCTGACTCTCT	AGAACCACCAGGGTCACAAG
Dnajc7	GATCCTGGGAGTGGACAAGA	TCCTTCTGAACTTCGGCACT
Hspa5	ATAAACCCCGATGAGGCTGT	GGGGACAAACATCAAGCAGT
Hsp90b1	GGATGCAGGGACAGAAGAAG	CTCCACACGGGATTCATAGC
Hspa4	GCAGAACAAACAGAGCCTGA	TGTTTTGGTTCCTCCTTTGG
Rpl41	AGGATGGCTACCATCACCTG	TCTTGGCAGCATACTCCTGA
Rps6kb2	AAGAGGGCTTCTCCTTCCAG	AACCCCTCAAAGGGAGAGAA
Rpl18a	CGCGAAAGACAACACTTCCT	GGTACAGTGGTGGTGTGTGG
Rps26	GGATCCTCTTTCGAGTCTTGG	TTCTTGATGGCCTTATCCTTG
Rps15	GAGAAGCCTGAGGTGGTGAA	CAGGTAGTGGCCGATCATCT
ATF4	GAAACCTCATGGGTTCTCCA	TCTCCAACATCCAATCTGTCC
Actin B	TTGCTGACAGGATGCAGAAG	ACATCTGCTGGAAGGTGGAC
Rps9	GACCAGGAGCTAAAGTTGATTGGA	GCGTCAACAGCTCCCGGGC
Cyclophilin	CAGACGCCACTGTCGCTT T	TGTCTTTGGAACTTTGTCTGCAA
