# Supplementary material for: ALS/FTD-Linked Mutation in FUS Suppresses Intra-axonal Protein Synthesis and Drives Disease Without Nuclear Loss-of-Function of FUS
Source: Neuron. 2018 Nov 21;100(4):816–830.e7. doi: 10.1016/j.neuron.2018.09.044 (PMC6277851; doi:10.1016/j.neuron.2018.09.044)
Supplement: Document S2. Article plus Supplemental Information [file mmc3.pdf]

Article

Neuron

ALS/FTD-Linked Mutation in FUS Suppresses Intra-axonal Protein Synthesis and Drives Disease Without Nuclear Loss-of-Function of FUS

Graphical Abstract

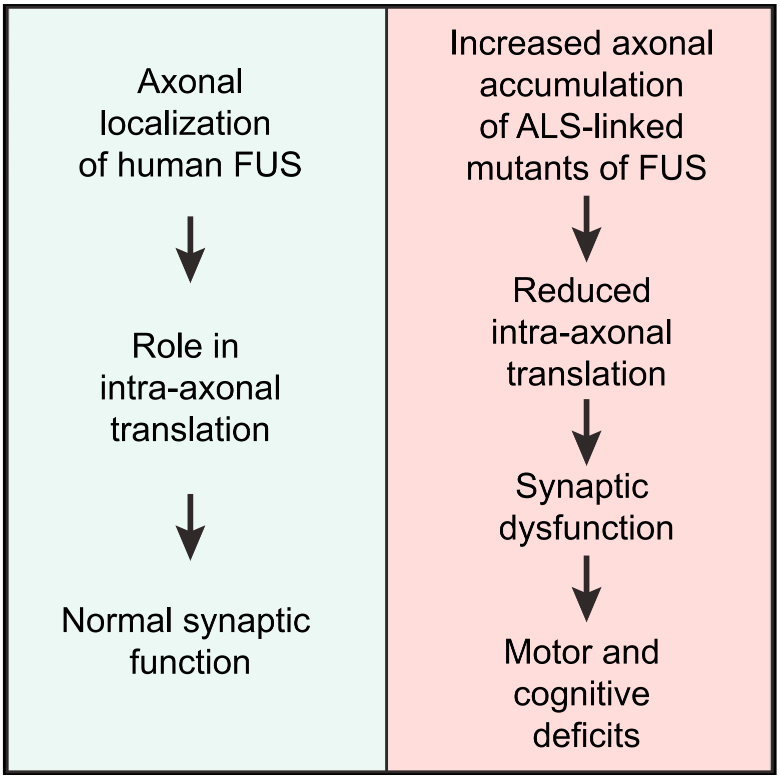

Highlights

- Toxicity, not loss of function, of ALS/FTD-linked mutant FUS drives disease
- Intra-axonal protein synthesis is inhibited by ALS/FTD-causing mutants in FUS
- Toxicity from ALS/FTD-linked mutants in FUS induces an integrated stress response
- ALS/FTD mutants in FUS reduce synaptic activity without loss of nuclear FUS

Authors

Jone López-Erauskin,
Takahiro Tadokoro,
Michael W. Baughn, ...,
Clotilde Lagier-Tourenne,
Don W. Cleveland, Sandrine Da Cruz

Correspondence

dcleveland@ucsd.edu (D.W.C.),
sdacruz@ucsd.edu (S.D.C.)

In Brief

Mutations in FUS are causative of ALS and frontotemporal dementia (FTD). López-Erauskin et al. show that disease-causing mutant FUS inhibits intra-axonal protein synthesis and provokes hippocampal synaptic loss and dysfunction without loss of nuclear FUS function or FUS aggregation.

López-Erauskin et al., 2018, Neuron *100*, 816–830
November 21, 2018 © 2018 The Author(s). Published by Elsevier Inc.
https://doi.org/10.1016/j.neuron.2018.09.044

CellPress

CellPress

Neuron
Article

ALS/FTD-Linked Mutation in FUS Suppresses Intra-axonal Protein Synthesis and Drives Disease Without Nuclear Loss-of-Function of FUS

Jone López-Erauskin,[1] Takahiro Tadokoro,[4] Michael W. Baughn,[1] Brian Myers,[1] Melissa McAlonis-Downes,[1] Carlos Chillon-Marinas,[1] Joshua N. Asiaban,[1] Jonathan Artates,[1] Anh T. Bui,[1] Anne P. Vetto,[1] Sandra K. Lee,[1] Ai Vy Le,[1] Ying Sun,[1] Mélanie Jambeau,[1,12] Jihane Boubaker,[1] Deborah Swing,[5] Jinsong Qiu,[2] Geoffrey G. Hicks,[6] Zhengyu Ouyang,[2] Xiang-Dong Fu,[2] Lino Tessarollo,[5] Shuo-Chien Ling,[1,9,10] Philippe A. Parone,[1,11] Christopher E. Shaw,[7,8] Martin Marsala,[4] Clotilde Lagier-Tourenne,[1,3,12] Don W. Cleveland,[1,2,3,13,*] and Sandrine Da Cruz[1,*]

[1]Ludwig Institute for Cancer Research, University of California at San Diego, La Jolla, CA 92093, USA
[2]Department of Cellular and Molecular Medicine, University of California at San Diego, La Jolla, CA 92093, USA
[3]Department of Neurosciences, University of California at San Diego, La Jolla, CA 92093, USA
[4]Department of Anesthesiology, University of California at San Diego, La Jolla, CA 92093, USA
[5]Mouse Cancer Genetics Program, National Cancer Institute, Frederick, MD, 21702, USA
[6]Regenerative Medicine Program and Department of Biochemistry & Medical Genetics, University of Manitoba, Winnipeg, R3T 2N2, Canada
[7]United Kingdom Dementia Research Institute Centre, Maurice Wohl Clinical Neuroscience Institute, Institute of Psychiatry, Psychology and Neuroscience, King's College London, SE5 9NU London, U.K
[8]Centre for Brain Research, University of Auckland, Auckland, New Zealand
[9]Present address: Department of Physiology, National University of Singapore, 117549, Singapore
[10]Present address: Program in Neuroscience and Behavior Disorders, Duke-NUS Graduate Medical School, Singapore
[11]Present address: Fate Therapeutics, 3535 General Atomics Court, San Diego, CA 9212, USA
[12]Present address: MassGeneral Institute for Neurodegenerative Disease, Department of Neurology, Massachusetts General Hospital, Harvard Medical School, Charlestown, MA 02129, USA
[13]Lead Contact
*Correspondence: dcleveland@ucsd.edu (D.W.C.), sdacruz@ucsd.edu (S.D.C.)
https://doi.org/10.1016/j.neuron.2018.09.044

SUMMARY

Through the generation of humanized FUS mice expressing full-length human FUS, we identify that when expressed at near endogenous murine FUS levels, both wild-type and ALS-causing and frontotemporal dementia (FTD)-causing mutations complement the essential function(s) of murine FUS. Replacement of murine FUS with mutant, but not wild-type, human FUS causes stress-mediated induction of chaperones, decreased expression of ion channels and transporters essential for synaptic function, and reduced synaptic activity without loss of nuclear FUS or its cytoplasmic aggregation. Most strikingly, accumulation of mutant human FUS is shown to activate an integrated stress response and to inhibit local, intra-axonal protein synthesis in hippocampal neurons and sciatic nerves. Collectively, our evidence demonstrates that human ALS/FTD-linked mutations in FUS induce a gain of toxicity that includes stress-mediated suppression in intra-axonal translation, synaptic dysfunction, and progressive age-dependent motor and cognitive disease without cytoplasmic aggregation, altered nuclear localization, or aberrant splicing of FUS-bound pre-mRNAs.

INTRODUCTION

Amyotrophic Lateral Sclerosis (ALS) is a neurodegenerative disorder leading to paralysis from death of motor neurons. Mutations in the gene encoding the RNA binding protein FUS (fused in sarcoma) are causative of cases of familial ALS (Kwiatkowski et al., 2009; Vance et al., 2009) as well as instances of frontotemporal dementia (FTD) (Broustal et al., 2010; Van Langenhove et al., 2010; Yan et al., 2010). FUS is present in the pathological inclusions of patients with FUS-mediated ALS and most of FTD instances without TDP-43 or Tau-containing aggregates, accounting for about 10% of the frontotemporal lobar degeneration (FTLD) cases, known as FTLD-FUS (Mackenzie et al., 2010).

FUS-containing aggregates have been reported in both the nucleus and the cytoplasm of neurons and glial cells in the central nervous system (CNS) of patients with *FUS* mutations or sporadic disease (Belzil et al., 2011; Chio et al., 2011; DeJesus-Hernandez et al., 2010; Kim et al., 2014b; Rademakers et al., 2010; Vance et al., 2009) as well as neurodegenerative conditions including Huntington's disease (Doi et al., 2008). The relocalization of FUS from the nucleus to the cytoplasm is incomplete, with at least some nuclear retention in neurons with FUS inclusions (Munoz et al., 2009; Neumann et al., 2009; Rademakers et al., 2010).

The 526-amino-acid FUS protein includes a glycine-rich, low-complexity, prion-like domain and a C-terminal, non-classical PY nuclear localization signal (PY-NLS) in which most of the ALS/FTLD-linked mutations are clustered (Da Cruz and Cleveland, 2011). Like TDP-43, in most cell types FUS is mainly nuclear

 © 2018 The Author(s). Published by Elsevier Inc.
This is an open access article under the CC BY license (http://creativecommons.org/licenses/by/4.0/).

(Andersson et al., 2008). Beyond nuclei, FUS has been reported in neurons to be enriched in dendrites (Belly et al., 2005; Yasuda et al., 2013), spines (Fujii et al., 2005), in close proximity of presynaptic vesicles (Schoen et al., 2016), and at the neuromuscular junctions (So et al., 2018). Reduction (Udagawa et al., 2015) or deletion (Hicks et al., 2000) of *Fus* in mouse hippocampal neurons causes abnormal spine morphology and density, consistent with a role for FUS in modulation of neuronal plasticity or synaptic activity—potentially through the alteration of mRNA stability (Udagawa et al., 2015) or transport and local translation in neurons (Fujii et al., 2005). While some studies have reported that a proportion of wild-type FUS is recruited to cytoplasmic stress granules, others have found that only FUS mutants localize to stress granules (Aulas and Vande Velde, 2015).

FUS has been proposed to participate in a range of cellular processes including transcription, splicing, RNA localization and degradation, and DNA damage (Lagier-Tourenne et al., 2010). Genome-wide analyses have reported that FUS binds between 5,000 and 8,000 mammalian RNA targets (reviewed in Nussbacher et al., 2015) and that deletion of the gene is associated with alterations in levels and splicing of ~1,000 mRNAs (Honda et al., 2013; Ichiyanagi et al., 2016; Kapeli et al., 2016; Lagier-Tourenne et al., 2012). Nevertheless, deletion of *Fus* from outbred mice (Kuroda et al., 2000) or from inbred mature mouse motor neurons does not cause their degeneration (Sharma et al., 2016). Efforts to model FUS-mediated disease in rodents have used heterologous promoters (that do not fully recapitulate the pattern of expression of endogenous FUS) to drive, in most cases, elevated levels of human wild-type (Mitchell et al., 2013; So et al., 2018) or mutant FUS (Huang et al., 2011; Qiu et al., 2014; Sephton et al., 2014; Sharma et al., 2016; Shelkovnikova et al., 2013; Shiihashi et al., 2016; Verbeeck et al., 2012). Others have used heterozygous or homozygous removal of the nuclear localization domain of mouse *Fus* (Devoy et al., 2017; Scekic-Zahirovic et al., 2017; Scekic-Zahirovic et al., 2016). Both sets of efforts have suggested that gain of toxicity is a component of pathogenesis. However, the identity(ies) of possible acquired toxicities remains unknown, and whether loss of function contributes to disease is not established.

We have now generated humanized FUS mice in which the full-length human *FUS* gene, encoding wild-type or ALS-linked mutations, replaces murine *Fus*. With expression levels close to the normal levels of endogenous FUS, wild-type or mutant FUS mimics the predominantly nuclear localization of endogenous FUS and complements its essential function(s). Mutant FUS is shown to cause progressive motor and cognitive deficits—without detectable cytoplasmic aggregation—accompanied by RNA alterations in expression that are driven by a gain-of-toxicity rather than a loss of FUS function. Most strikingly, mutant FUS is shown to inhibit local intra-axonal protein translation, drive synaptic loss, elevate stress-induced chaperones, and suppress RNAs encoding ion channels and transporters essential for synaptic function.

RESULTS

Mice with Human FUS Expression Mimicking Murine FUS

Transgenic mice were generated in a C57BL/6 congenic background to express wild-type (WT) FUS or either of two ALS-linked mutations (R521C and R521H), each transcribed from the endogenous human *FUS* promoter (Figure 1A). Twenty-six founder mice were obtained expressing human FUS WT (hg*FUS*^{WT}), R521C (hg*FUS*^{R521C}), or R521H (hg*FUS*^{R521H}) (Figure S1A). Eight lines were established (Figure S1B).

Accumulated human FUS levels were comparable in most lines using an antibody recognizing both human and mouse FUS proteins with equivalent affinity (Figures 1B, S1D, and S1E). Endogenous FUS was reduced by more than 50% at mRNA (Figures 1C and S1C) and protein (Figures 1B and 1D) levels, resulting in overall FUS protein levels similar to endogenous mouse FUS levels in non-transgenic (Non-Tg) animals (Figure 1B). Lines with matching levels (line 88 for WT, line 10 for R521C, and line 9 for R521H; Figures 1C and 1D) and expression pattern of human FUS (determined in spinal cord sections stained with an antibody specifically recognizing human, but not mouse, FUS) mimicking that of the endogenous mouse FUS (Figure 1E) were further characterized.

Age-Dependent Motor Deficits Without Nuclear FUS Mislocalization or Cytoplasmic Aggregation in ALS/FTD-Linked Mutant FUS Mice

Motor performance of all three FUS (hg*FUS*^{WT}, hg*FUS*^{R521C}, and hg*FUS*^{R521H}) mouse lines at 2 months of age was undistinguishable from that of Non-Tg littermates. By 8 months, both mutant FUS lines had developed significant reduction in hindlimb grip strength compared to age-matched Non-Tg and hg*FUS*^{WT} littermates (Figure 2A). Motor dysfunction in both mutant lines was further exacerbated during aging, reaching a loss of grip strength of over 50% by 18 months. Loss of motor performance was accompanied by age-dependent loss of muscle innervation (Figures 2B and 2C), α-motor axons (Figures 2D–2F), and spinal cord motor neurons (Figures 2G and 2H). There was also a modest decline in the number of innervated neuromuscular junctions (NMJs) (Figure 2C) and α-motor axons in age-matched hg*FUS*^{WT} mice relative to Non-Tg animals (Figures 2E and 2F).

Loss of motor neurons in mutant animals was accompanied by increased activation of astrocytes (Figures S2A and S2C) and microglia (Figures S2B and S2C) in spinal cord ventral horns of hg*FUS*^{R521C} and hg*FUS*^{R521H} mice (scored by GFAP and Iba1 immunoreactivity, respectively). FUS protein levels remained unchanged up to 24 months of age across all genotypes (Figures 2I and 2J). Similarly, although elevated expression of mutant FUS^{R521C} driven by a prion promoter has been proposed to cause accumulation of DNA damage in mouse spinal cords (Qiu et al., 2014), no such damage (measured by γH2A.X and 53BP1 co-localization) was observable in aged, diseased hg*FUS*^{R521C} and hg*FUS*^{R521H} mice (Figure S2D), although it was obvious in the spinal cords of γ-irradiated animals.

Age-dependent motor deficits in ALS-causing mutant FUS lines were not accompanied by cytoplasmic redistribution of either human (Figures 2K and S2E) or mouse (Figures 2L and S2E) FUS or FUS aggregates (Figures 2K–2L and S2F) in 24-month-old spinal cords. Taken together, neither FUS aggregates nor redistribution to the cytoplasm are necessary for age-dependent motor deficits in the hg*FUS*^{R521C} and hg*FUS*^{R521H} lines.

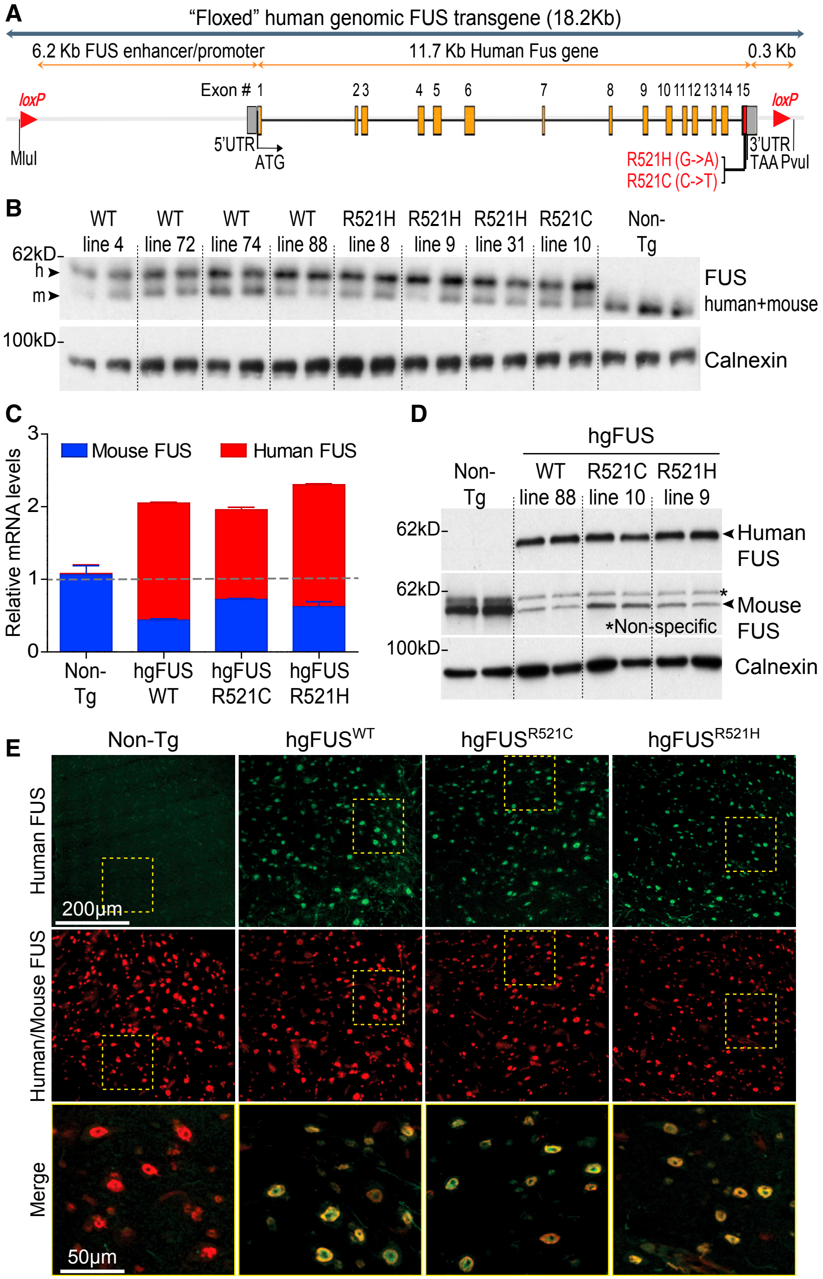

Figure 1. Expression Levels and Cellular Pattern of the Human FUS Transgene Mimics That of Endogenous Protein with an Auto-regulation Mechanism

(A) Schematic of the human BAC containing human *FUS* gene wild-type (WT) or either ALS-linked mutation R521C or R521H.

(B) Protein levels of the 54 and 53kDa human and mouse FUS using an antibody recognizing both species with equal affinity. Calnexin was used as a loading control. Each lane represents an independent mouse spinal cord.

(C) Expression levels of total (mouse, blue bars and human, red bars) *FUS* RNAs in Non-Tg, hgFUS^{WT}, hgFUS^{R521C}, and hgFUS^{R521H} spinal cords. Data are represented as mean ± SEM (n = 3 per group). See also Figure S1.

(D) Protein levels of the human FUS transgene using an antibody specific to the human protein and mouse FUS using an antibody specifically recognizing the mouse protein in 2-month-old animals (* non-specific band). Each lane represents an independent mouse spinal cord per transgenic line. Calnexin was used as a loading control.

(E) Lumbar spinal cord sections from 2-month-old Non-Tg, hgFUS^{WT}, hgFUS^{R521C}, and hgFUS^{R521H} mice immunostained for human FUS transgene (green) and total FUS (red). Scale bars, 200 and 50 µm.

ALS/FTD-Linked Mutations in FUS Drive Age-Dependent Cognitive Deficits and Synaptic Loss

Age-dependent deficits in cognition and memory were developed in hgFUS^{R521C} and hgFUS^{R521H} mice, including novel object recognition (Figure 3A), spatial memory (Figure S3A), sociability (Figures S3B and S3C) and anxiety (Figure S3D). These alterations were accompanied by modest increases in astrogliosis (Figures 3B and 3C) and microgliosis (Figures 3B and 3D), as well as a significant loss of synapses (marked by loss of synapsin; Figures 3E and 3F) in the hippocampus of aged, 24-month-old mice, without loss of NeuN positive neurons. Mutant-FUS-dependent loss of synapsin was recapitulated in cultured primary hippocampal neurons (Figure 3G). Neuronal activity measurements (using multi-electrode arrays [MEA]; Chailangkarn et al., 2016) revealed age- and mutant-dependent synaptic dysfunction, with a 50% decrease in spike rate developing by 28 days of culture (Figures 3H and 3I).

Progressive Neurodegeneration in Humanized Mutant FUS Mice

We next generated humanized FUS mice (m$Fus^{-/-}$/hg*FUS* mice) in which the sole source of FUS was human FUS. This was achieved by mating to produce mice that expressed our wild-type or mutant human FUS-encoding transgenes but in which both endogenous *Fus* alleles were inactivated (Figure 4A). While homozygous disruption of murine *Fus* is lethal in C57BL/6 mice, as previously reported (Hicks et al., 2000), lethality was completely rescued by expression of human wild-type or mutant FUS, and humanized FUS mice (either WT or mutants) were born with the expected Mendelian ratios (Figures 4B and 4C). Nevertheless, despite comparable human FUS mRNA (Figure S4A) and protein levels (Figure 4C), beginning at 6 months of age, hgFUS^{R521C} or hgFUS^{R521H} mice developed progressive motor deficits including age-dependent loss of hindlimb grip strength (Figure 4D) and loss of spinal cord ChAT-positive motor neurons (Figure S4B). Remarkably, reduction or elimination of endogenous *Fus* did not exacerbate mutant FUS-dependent motor deficits and neurotoxicity (Figures 2 and 4).

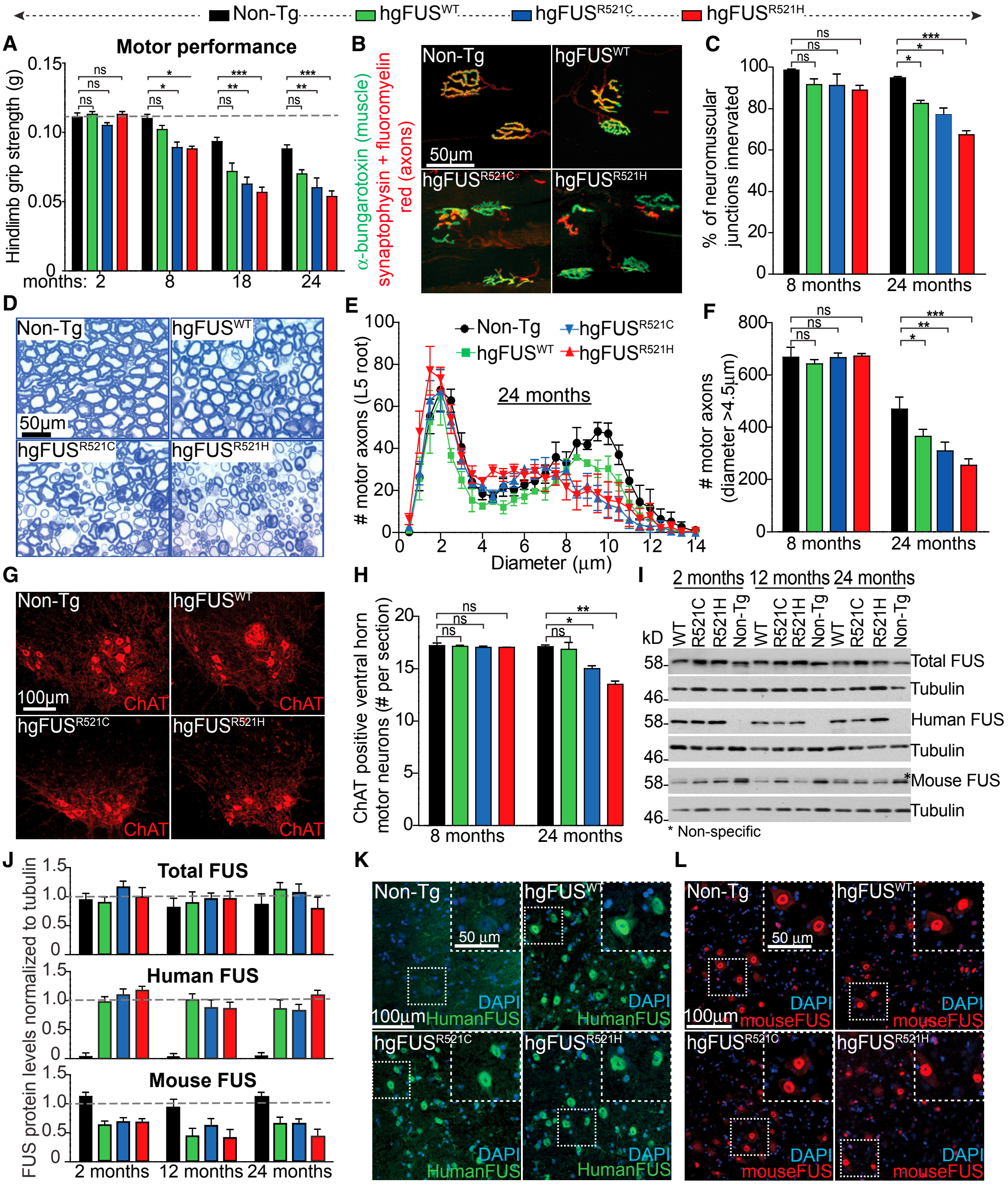

Figure 2. ALS-Linked FUS Mutants Show Age-Dependent Progressive Motor Impairments without Nuclear Mislocalization or Aggregation of FUS

(A) Hindlimb grip strength measured up to 24 months. Data are represented as mean ± SEM (n ≥ 12 per group). *p < 0.05, **p < 0.01, ***p < 0.001, ns: non-significant using one-way ANOVA.

(B) Gastrocnemius muscle sections from 24-month-old Non-Tg, hg*FUS*^{WT}, hg*FUS*^{R521C}, and hg*FUS*^{R521H} mice with axons immunostained using a synaptophysin antibody and FluoroMyelin™ red (red) and muscle endplates using α-bungarotoxin (green). Scale bar, 50 μm.

(C) NMJ innervation of the gastrocnemius from 8- and 24-month-old Non-Tg, hg*FUS*^{WT}, hg*FUS*^{R521C}, and hg*FUS*^{R521H} mice. Data are represented as mean ± SEM (n ≥ 3 mice per group). *p < 0.05, ***p < 0.001, two-sided unpaired Student's t test.

(D) Lumbar motor axons from 24-month-old Non-Tg, hg*FUS*^{WT}, hg*FUS*^{R521C}, and hg*FUS*^{R521H} mice. Scale bar, 50 μm.

(E) Size distribution per motor axon in the L5 motor root of 24-month-old Non-Tg, hg*FUS*^{WT}, hg*FUS*^{R521C}, and hg*FUS*^{R521H} mice. Data are represented as mean ± SEM (n ≥ 3 per group). *p < 0.05, **p < 0.01 and ***p < 0.001, two-sided unpaired Student's t test.

(legend continued on next page)

ALS/FTD-Mediated Disease Without Loss of Normal FUS Function

To determine whether age-dependent loss of FUS nuclear function contributes to FUS-mediated disease in aged, humanized FUS mice, levels (Figure 4E) and splicing (Figure 4F) of the genes most affected by FUS depletion in mice (Lagier-Tourenne et al., 2012) were assessed. Expression levels of *Kcnip4*, *Park2*, *Smyd3*, *Mal*, *Nrxn3*, *Nlgn1*, and *Csmd1* were significantly decreased in the CNS of mice treated with reduced FUS following antisense oligonucleotide-mediated degradation of *Fus* mRNAs (Figure 4E; Lagier-Tourenne et al., 2012). However, a modest increase, rather than a reduction, was seen in the expression of these genes in spinal cords of humanized hg*FUS*^{R521C} and hg*FUS*^{R521H} mice compared to Non-Tg and hg*FUS*^{WT} mice depleted of endogenous FUS (Figure 4E).

Genome-wide expression analysis (RNA-seq; Figure S4C) of aged, humanized mutant FUS spinal cords did not significantly overlap with the changes identified upon FUS depletion in adult mouse spinal cords (Kapeli et al., 2016) (Figure S4E). Similarly, the most common splicing changes identified upon depletion of FUS (Lagier-Tourenne et al., 2012) were not observed in the humanized FUS mouse spinal cords (Figure 4F). Global splicing analysis using the RNA-mediated oligonucleotide annealing, selection, and ligation with next-generation sequencing (RASL-seq) method (which permitted quantitative profiling of 3,859 alternative splicing events that correspond to exon inclusion or skipping events conserved between mouse and human; Scekic-Zahirovic et al., 2016; Sun et al., 2015; Zhou et al., 2012) did not reveal significant splicing alterations in aged, humanized mutant FUS spinal cords (Figure S4D). Altogether, these results demonstrate that the age-dependent motor deficits associated with expression of ALS-linked FUS mutant in humanized FUS mice cannot be caused by a loss of function of FUS in regulating gene expression and/or splicing.

A Mutant FUS-Dependent Gain of Toxicity RNA Signature Affecting Protein Synthesis

RNA expression profiles from spinal cords of humanized mutant FUS mice were used to test whether ALS-linked mutations produced expression changes that reflected gain of aberrant function (Figures 5 and S4E). RNA profiles from normal (Non-Tg) and humanized hg*FUS*^{WT} mice were almost indistinguishable. However, both humanized mutant FUS lines had highly distinct RNA profiles determined with unsupervised hierarchical clustering (Figure 5A) and principal component analysis (PCA) (Figure 5B), with 1,057 significant expression changes (defined by $p < 0.05$ adjusted for multiple testing; Table S1) and with 709 down and 348 upregulated genes (Figure 5C) relative to age-matched Non-Tg or humanized hg*FUS*^{WT} littermates. These changes (Figures 5D and 5E) included altered signaling pathways for protein translation mediated by eIF2α, a crucial translation initiation factor which forms a complex with components including eIF2B, GTP, and the initiating methionine tRNA (Kapur et al., 2017). mTOR and glutamate receptor signaling pathways (Figure 5E) were also affected.

mRNAs encoding multiple ion channels and transporters essential for synaptic function were decreased in spinal cord extracts of both humanized mutant FUS lines (Figure S5A), consistent with mutant FUS-dependent synaptic dysfunction (Figures 3E and 3F). Further tests using RT-qPCR confirmed reductions in ion channel and transporter components tested (reductions in nine are shown in Figure 5F). These changes were accompanied by the upregulation of ten genes encoding chaperones (Figure S5B), five of which were further validated by qRT-PCR (Figure 5G). Genes encoding ribosomal proteins represented some of the most downregulated genes, including sixteen components of the translation machinery (Figures 5H and S5C).

Stress-Mediated Inhibition of Intra-axonal Protein Synthesis from ALS/FTD-Linked Mutations in FUS

FUS mutant-dependent effects on protein translation mediated through eIF2α were tested in the nervous system of the humanized FUS mice. Phosphorylation of eIF2α on serine 51 inhibits overall initiation by blocking the activity of its eIF2B guanine exchange factor (Donnelly et al., 2013). Consistent with inhibited translation initiation, elevated levels of phosphorylated eIF2α (P-eIF2α), together with increased levels of HSF1 (heat shock transcription factor 1), were identified in spinal cord protein extracts from FUS adult mutant mice at early symptom onset (Figure 6A, S6A, and S6B).

ATF4 is a key effector of the integrated stress response that drives phosphorylation of eIF2α (Pakos-Zebrucka et al., 2016). Consistent with P-eIF2α dependent protein synthesis inhibition, there was a mutant FUS-dependent increase of *Atf4* mRNA levels in primary mouse hippocampal neurons (Figure 6B). Correspondingly, mRNA translation was reduced (compared with neurons of wild-type mice) by 25% in metabolically labeled (with ^{35}S-Met/Cys) cultured hippocampal neurons from mutant FUS mice (Figure 6C).

Recognizing that puromycin incorporation into nascent polypeptides enables direct visualization of actively translating ribosomes (using an anti-puromycin antibody; tom Dieck et al.,

(F) Total number of α-motor axons (caliber size > 4.5 μm) in the L5 motor root of 8- and 24-month-old Non-Tg, hg*FUS*^{WT}, hg*FUS*^{R521C}, and hg*FUS*^{R521H} mice. Data are represented as mean ± SEM (n $\geq$ 3 per group).

(G) Lumbar spinal cord sections from 24-month-old Non-Tg, hg*FUS*^{WT}, hg*FUS*^{R521C}, and hg*FUS*^{R521H} mice immunostained using a ChAT antibody to reveal cholinergic motor neurons. Scale bar, 100 μm.

(H) Total number of ChAT-positive motor neurons in lumbar spinal cords of 8- and 24-month-old Non-Tg, hg*FUS*^{WT}, hg*FUS*^{R521C}, and hg*FUS*^{R521H} mice. Data are represented as mean ± SEM (n $\geq$ 3 per group). *p < 0.05, **p < 0.01, two-sided unpaired Student's t test.

(I) Total human and/or mouse FUS protein levels in spinal cords of 2-, 12-, and 24-month-old hg*FUS*^{WT}, hg*FUS*^{R521C}, hg*FUS*^{R521H}, and Non-Tg mice. Tubulin was used as a loading control. Asterisk illustrates a non-specific band recognized by the antibody against mouse FUS.

(J) Quantification of immunoblots shown in (I). Data are represented as mean ± SEM from two independent experiments.

(K and L) Lumbar spinal cord sections from 24-month-old Non-Tg, hg*FUS*^{WT}, hg*FUS*^{R521C}, and hg*FUS*^{R521H} mice immunostained using an antibody recognizing specifically (K) human or (L) mouse FUS. DNA is stained with DAPI. Scale bars, 100 μm and 50 μm (inset). See also Figure S2.

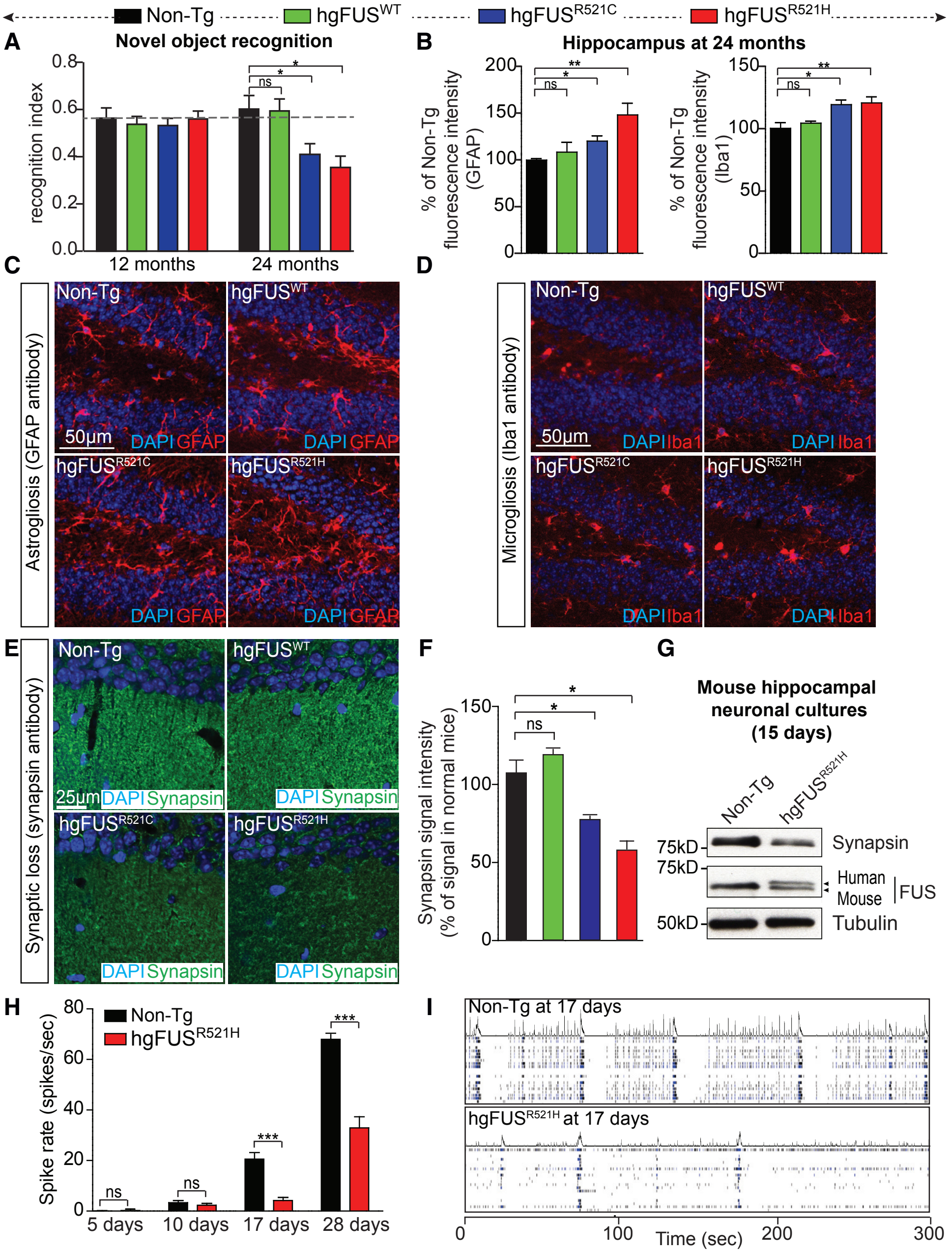

Figure 3. Age-Dependent Progressive Cognitive Impairments, Astrogliosis, and Microgliosis in Hippocampus of Mice Expressing ALS-Linked FUS Mutants

(A) Novel object recognition was measured in 12- and 24-month old Non-Tg, hg*FUS*^{WT}, hg*FUS*^{R521C}, and hg*FUS*^{R521H} mice. Data are represented as mean ± SEM (n = 15 per group). *p < 0.05 using one-way ANOVA. See also Figure S3.

(B) Quantification of the GFAP and Iba1 relative fluorescence intensities in hippocampal sections of 24-month-old Non-Tg, hg*FUS*^{WT}, hg*FUS*^{R521C}, and hg*FUS*^{R521H} mice. The bar graph represents mean ± SEM (n = 3 per group). *p < 0.05, **p < 0.01, two-sided unpaired Student's t test.

(C and D) Hippocampal sections from 24-month-old Non-Tg, hg*FUS*^{WT}, hg*FUS*^{R521C}, and hg*FUS*^{R521H} mice immunostained using an antibody detecting activated (C) astrocytes (GFAP) or (D) microglia (Iba1). DNA is stained with DAPI. Scale bar, 50 μm.

(E) Hippocampus from 24-month-old Non-Tg, hg*FUS*^{WT}, hg*FUS*^{R521C}, and hg*FUS*^{R521H} mice immunostained to reveal synapses (green) using a synapsin antibody and DAPI for nuclei. Scale bar, 25 μm.

(F) Quantification of the synapsin relative fluorescence intensity shown in (E). The bar graph represents mean ± SEM (n = 3 per group). *p < 0.05, two-sided unpaired Student's t test.

(G) Synapsin and FUS protein levels in hippocampal neuronal extracts from mutant hg*FUS*^{R521H} or Non-Tg mice (at 15 days of culture). Tubulin antibody was used as loading control.

(legend continued on next page)

2015), we cultured hippocampal neurons in compartmented microfluidic chambers that allow unambiguously distinguishing cell bodies from processes, prior puromycin pulse-labeling (Figures 6D and 6E). Surprisingly, translation was found to be predominantly reduced in processes (Figures 6E, 6G, 6I, and 6K) relative to cell bodies (Figures 6F, 6H, 6J, S6C, and S6D). This reduction was accompanied by increased phosphorylation of eIF2α in axons (Figures 6G and 6M), with no differences observed in cell bodies (Figures 6F and 6L). No cell death was detected at any time point (Figures S6E and S6F).

While a majority of FUS localized intranuclearly (Figure 6H, red), both wild-type and mutant human FUS were present in axons of hippocampal neurons cultured in compartmented microfluidic chambers (Figure 6I). FUS was not elevated in the cytoplasm of mutant neuronal cell bodies (Figures 6H and 6N). However, relative to wild-type FUS, significantly more mutant FUS accumulated within axons (Figures 6I and 6O). Since measurement of the volume covered by the axonal network in our hippocampal neuronal cultures revealed that it was at least 6 times that of their cell bodies and dendrites, this strong reduction in intra-axonal protein translation is likely to be sufficient to account for the 25% decreased protein synthesis measured in the total protein extract (Figure 6C).

To test mutant FUS effects on local translation in motor axons in mice, puromycin pulse labeling of sciatic nerve axons was achieved by its systemic administration into the mice, as previously reported (Goodman et al., 2011; Khoutorsky et al., 2015; Figure 7A). Increased phosphorylation of eIF2α (green signal by immunofluorescence) was observed in sciatic nerve axons (neurofilament positive; red) in both hg*FUS*^{R521C} and hg*FUS*^{R521H} mice at early symptom onset, but not in age-matched Non-Tg or hg*FUS*^{WT} animals (Figures 7B and 7E). Consistent with our findings in the hippocampal neurons (Figures 6E–6O and S6C–S6D), intra-axonal protein synthesis (revealed by decreased puromycin signal) was almost eliminated (Figures 7C–7D and 7F), along with increased accumulation of ALS-linked mutants of FUS (Figures 7D and 7G) within sciatic nerve axons of the two mutant FUS lines, but not their Non-Tg and hg*FUS*^{WT} littermates. Taken together, our *in vitro* and *in vivo* evidence demonstrates that ALS-linked mutations of FUS impair local protein synthesis within axons.

DISCUSSION

A key question regarding pathogenic mechanisms in FUS-mediated disease has been whether ALS-linked mutations in *FUS* cause neurodegeneration through a loss and/or gain of toxicity. By producing and analyzing humanized FUS mice in which wild-type or ALS-causing mutations in human *FUS* replace endogenous murine *Fus*, we demonstrate that both human wild-type and ALS/FTD-linked FUS mutants complement the essential functions of murine FUS. When expressed at a level and subcellular distribution that mimic endogenous FUS, mutant human FUS provokes progressive motor and cognitive deficits in mice that are accompanied by RNA alterations driven by a gain of toxicity rather than a loss of function of FUS, but without detectable cytoplasmic aggregation.

While high levels of wild-type FUS cause early lethality in mice (Mitchell et al., 2013; Sephton et al., 2014), our mice expressing wild-type FUS at levels close to the normal level of murine FUS also developed motor deficits during aging. When taken with a report that ALS patients with mutations in the 3′ UTR region of FUS accumulate those variants at increased levels in patient fibroblasts (Sabatelli et al., 2013), it is therefore likely that even a modest increase in FUS (human mutant or wild-type) is sufficient to trigger age-dependent motor deficits.

To an earlier report that FUS depletion from iPSC-derived motor neurons produces a different RNA signature than a disease-linked FUS mutation (Kapeli et al., 2016), our analyses have identified that the overwhelming majority of gene expression alterations associated with age-dependent motor and cognitive deficits in humanized mutant FUS mice do not overlap with those altered upon depletion of FUS (Kapeli et al., 2016; Lagier-Tourenne et al., 2012). Depletion of FUS in the adult mouse CNS leads to more than 300 splicing changes (Kapeli et al., 2016; Lagier-Tourenne et al., 2012) and disrupts non-canonical, U12-dependent splicing of RNAs (Reber et al., 2016) in a neuronal cell line. While cytoplasmic aggregates of an ALS-linked FUS mutant were reported to inhibit U12-mediated splicing of a subset of introns by trapping of snRNAs in aggregates (Reber et al., 2016), and ALS-linked mutant-dependent splicing changes have previously been reported in patient fibroblasts (Sun et al., 2015) as well as in motor neuron precursor cells derived from iPSCs (Ichiyanagi et al., 2016), no significant splicing changes in the nervous system of humanized mutant FUS mice were identified despite initiation of adult-onset disease. The major splicing alterations identified upon *in vivo* FUS depletion (Lagier-Tourenne et al., 2012) were not recapitulated in diseased, mutant humanized FUS mice. Thus, FUS-mediated neurodegeneration in mice cannot be caused primarily by reduced FUS activity, a conclusion fully consistent with the finding that deletion of FUS from mature motor neurons does not provoke disease (Sharma et al., 2016).

Several mechanisms underlying FUS-mediated toxicity have now been proposed, including aggregation and redistribution of mutant FUS into the cytoplasm (Dormann et al., 2012; Mackenzie et al., 2011; March et al., 2016). Morphology and distribution of FUS inclusions vary among the CNS of different neurodegenerative conditions, with FUS inclusions reportedly being nuclear in Huntington's disease (Doi et al., 2008) but primarily cytoplasmic in ALS and FTLD (King et al., 2015; Mackenzie et al., 2011), with some nuclear retention in even the most affected neurons with FUS inclusions (Munoz et al., 2009;

(H) Spontaneous neuronal spike rate (spikes per s) measured by multi-electrode arrays (MEA) in hippocampal neurons from mutant hg*FUS*^{R521H} compared to Non-Tg mice cultured under basal conditions. Data are represented as mean ± SEM (n = 4–8 numbers of wells per experiment from three independent experiments). ***$p < 0.001$, two-sided unpaired Student's t test.
(I) Raster plots from MEA recordings showing neuronal spikes and burst of spikes in wild-type and mutant hg*FUS*^{R521H} hippocampal neurons for 300 s. Each lane represents spikes per s recorded per electrode.

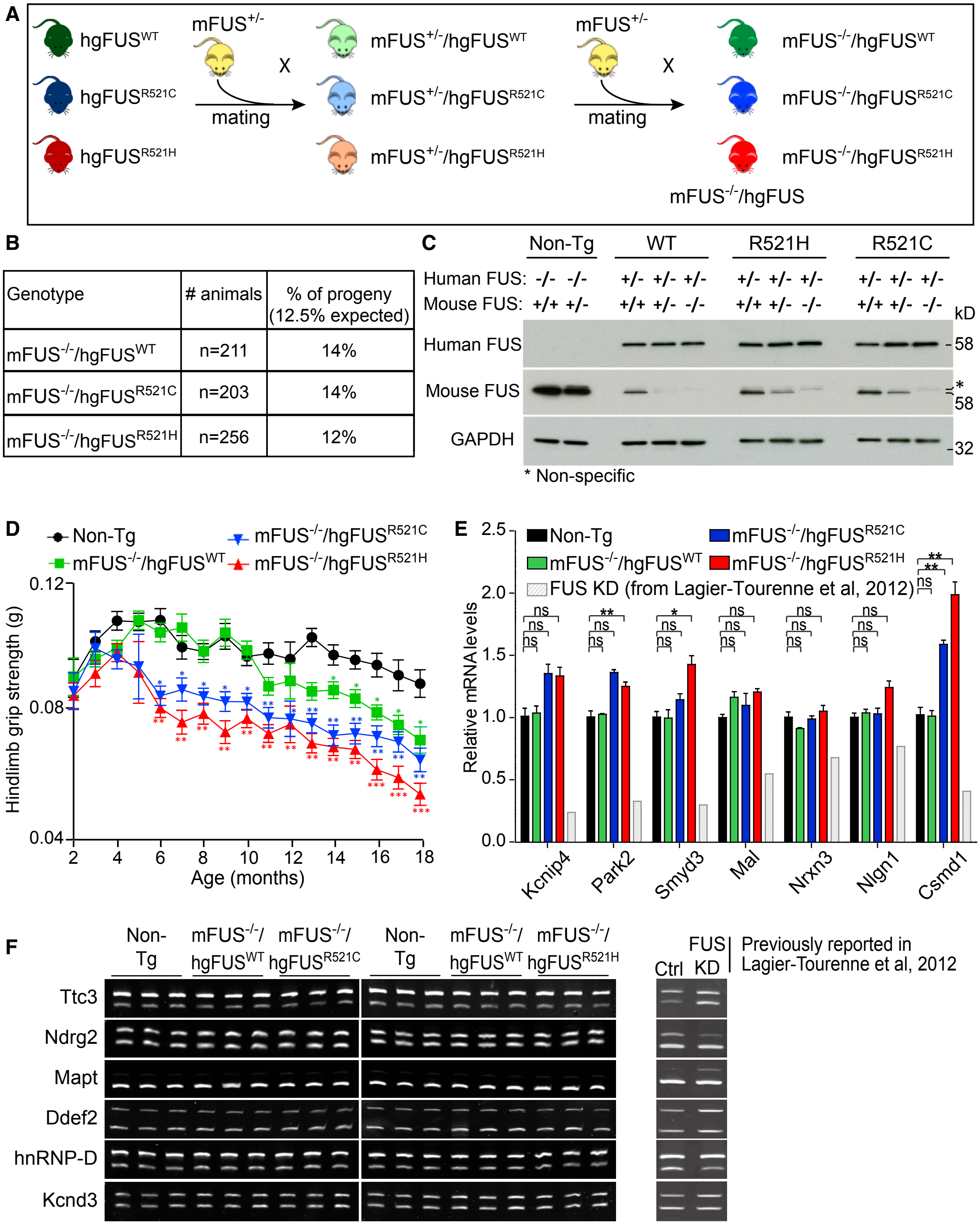

Figure 4. Progressive Motor Deficits Associated with ALS-Linked FUS Mutants Without Reduction in FUS Function

(A) Schematic of the two-round mating of hg*FUS* mice with FUS mice heterozygous for mouse FUS (m*Fus*$^{+/-}$) to produce animals in which all FUS is human wild-type or mutant (m*Fus*$^{-/-}$/hg*FUS*).

(B) Number and Mendelian ratio of animals obtained from the two-step mating.

(C) Human and mouse FUS protein accumulation in 2-month old mouse spinal cords using FUS antibodies specific for human and mouse proteins. GAPDH was used as a loading control. Asterisk illustrates a non-specific band detected with the mouse-specific FUS antibody.

(D) Hindlimb grip strength measured bi-weekly from 2 to 18 months of age. Data are represented as mean ± SEM (n ≥ 15 per group). *p < 0.05, **p < 0.01 and ***p < 0.001 using one-way ANOVA.

(legend continued on next page)

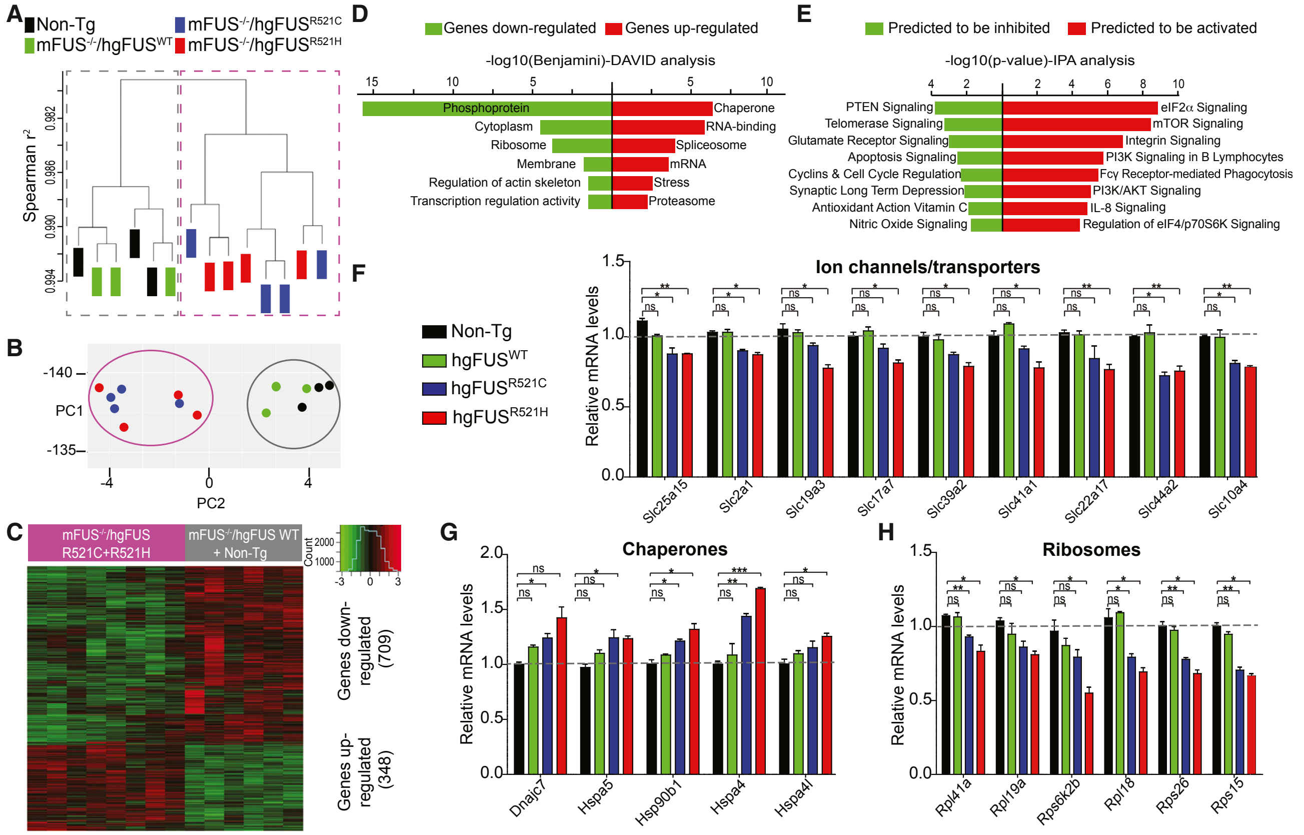

Figure 5. ALS-Linked Mutations in FUS Cause a Mutant-Dependent Signature of RNAs that Includes a Reduction in mRNAs for Ion Channels and Transporters and Ribosomal Proteins and an Increase in mRNAs Encoding Chaperones

(A) Unsupervised hierarchical clustering of all expressed genes in spinal cords of 18-month-old Non-Tg, m*Fus*$^{-/-}$/hg*FUS*^{WT}, m*Fus*$^{-/-}$/hg*FUS*^{R521C}, and m*Fus*$^{-/-}$/hg*FUS*^{R521H} mice identified by RNA-seq analysis.

(B) Principal component analysis (PCA) of differentially expressed genes in 18-month-old Non-Tg, m*Fus*$^{-/-}$/hg*FUS*^{WT}, m*Fus*$^{-/-}$/hg*FUS*^{R521C}, and m*Fus*$^{-/-}$/hg*FUS*^{R521H} spinal cords.

(C) Heatmap with hierarchical clustering of 1,057 genes differentially expressed in 18-month-old hg*FUS*^{R521C} and hg*FUS*^{R521H} mice compared to age-matched Non-Tg and m*Fus*$^{-/-}$/hg*FUS*^{WT} spinal cords.

(D) Functional analysis using DAVID software of the differentially expressed genes revealing the most enriched gene group changes in m*Fus*$^{-/-}$/hg*FUS*^{R521C} and m*Fus*$^{-/-}$/hg*FUS*^{R521H} mice compared to age-matched Non-Tg and m*Fus*$^{-/-}$/hg*FUS*^{WT} animals.

(E) Functional analysis using the Ingenuity Pathway Analysis (IPA) software of the differentially expressed genes revealing the most enriched gene group changes in m*Fus*$^{-/-}$/hg*FUS*^{R521C} and m*Fus*$^{-/-}$/hg*FUS*^{R521H} mice compared to age-matched Non-Tg and m*Fus*$^{-/-}$/hg*FUS*^{WT} animals.

(F) Reduced expression of ion channels and transporters essential for synaptic function in 18-month-old m*Fus*$^{-/-}$/hg*FUS*^{R521C} and m*Fus*$^{-/-}$/hg*FUS*^{R521H} spinal cords compared to age-matched Non-Tg and m*Fus*$^{-/-}$/hg*FUS*^{WT}, validated by qRT-PCR. Data are represented as mean ± SEM (n ≥ 3 per group). $^{*}p < 0.05$, $^{**}p < 0.01$, two-sided unpaired Student's t test. See also Figure S5A.

(G) Increased expression of chaperones in 18-month-old m*Fus*$^{-/-}$/hg*FUS*^{R521C} and m*Fus*$^{-/-}$/hg*FUS*^{R521H} spinal cords compared to age-matched Non-Tg and m*Fus*$^{-/-}$/hg*FUS*^{WT}, validated by qRT-PCR. Data are represented as mean ± SEM (n ≥ 3 per group). $^{*}p < 0.05$, $^{**}p < 0.01$ and $^{**}p < 0.001$, two-sided unpaired Student's t test. See also Figure S5B.

(H) Reduced expression of genes encoding ribosomes or other components of the translation machinery in 18-month-old m*Fus*$^{-/-}$/hg*FUS*^{R521C} and m*Fus*$^{-/-}$/hg*FUS*^{R521H} spinal cords compared to age-matched Non-Tg and m*Fus*$^{-/-}$/hg*FUS*^{WT}, validated by qRT-PCR. Data are represented as mean ± SEM (n ≥ 3 per group). $^{*}p < 0.05$ and $^{**}p < 0.01$, two-sided unpaired Student's t test. See also Figure S5C.

Neumann et al., 2009; Rademakers et al., 2010). To these preceding efforts, we report that age-dependent motor and cognitive deficits develop in humanized mutant FUS mice without detectable human FUS cytoplasmic aggregation. Thus, while we cannot rule out FUS aggregates in the cytoplasm in forms undetectable by our analyses, large aggregates similar to

(E) Expression levels of candidate genes (known to be reduced in the CNS of mice depleted of FUS [light gray], previously reported in Lagier-Tourenne et al., 2012; figure 3I) in 18-month-old Non-Tg, m*Fus*$^{-/-}$/hg*FUS*^{WT}, m*Fus*$^{-/-}$/hg*FUS*^{R521C}, and m*Fus*$^{-/-}$/hg*FUS*^{R521H} mouse spinal cords. Data are represented as mean ± SEM (n ≥ 3 per group). $^{*}p < 0.05$, $^{**}p < 0.01$, two-sided unpaired Student's t test.

(F) Splicing profile of candidate genes (known to be altered in the CNS of mice depleted of FUS, previously reported in Lagier-Tourenne et al., 2012; figure 4C) in 18-month-old Non-Tg, m*Fus*$^{-/-}$/hg*FUS*^{WT}, m*Fus*$^{-/-}$/hg*FUS*^{R521C}, and m*Fus*$^{-/-}$/hg*FUS*^{R521H} mouse spinal cords. See also Figure S4.

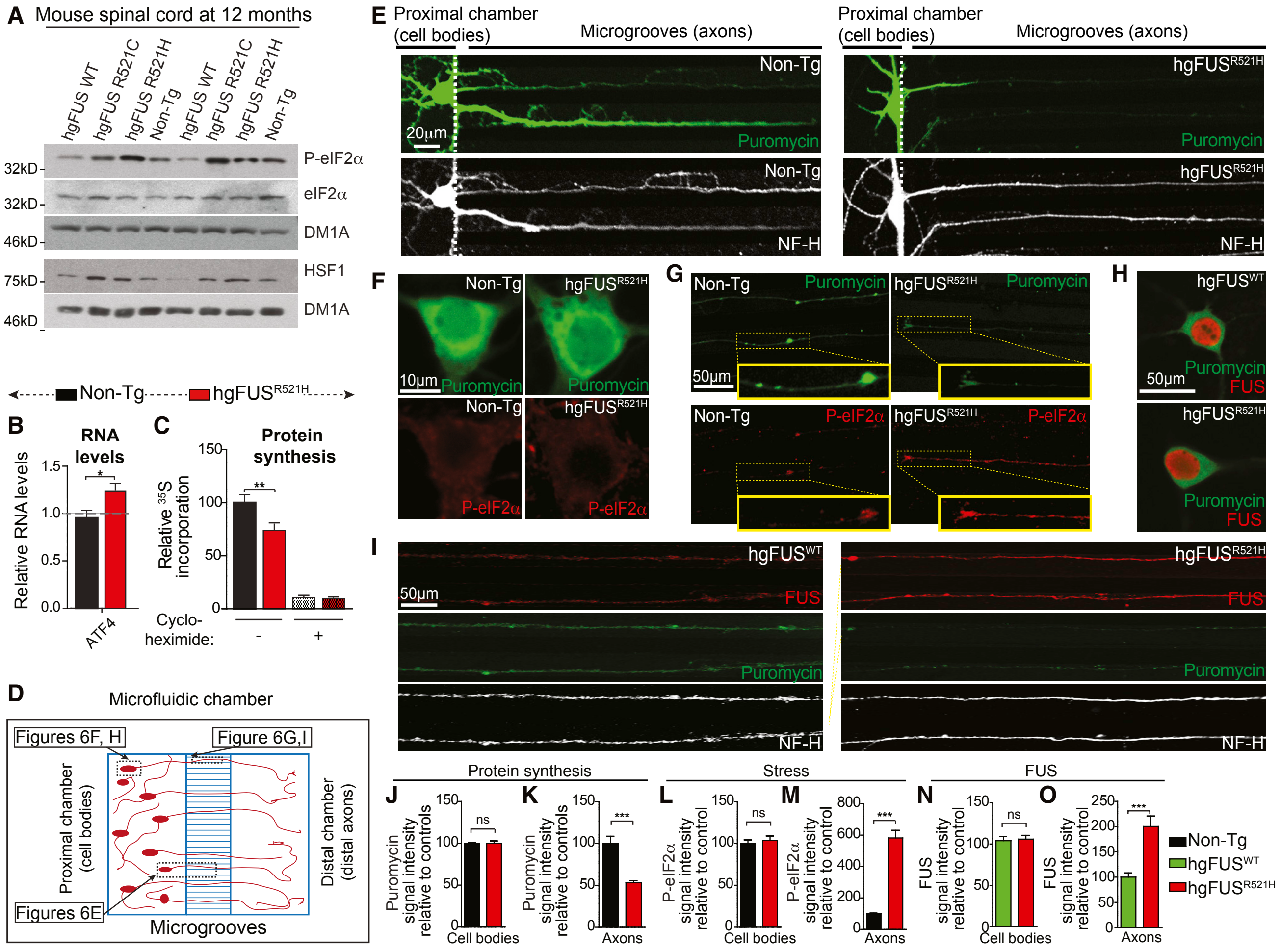

Figure 6. ALS-Linked Mutations in FUS Cause Stress-Mediated Reduction in Axonal Protein Synthesis

(A) Immunoblot demonstrating increased stress mediated by mutant FUS at early symptom onset using the phosphorylated form of eIF2α antibody (P-eIF2α) and HSF1 antibody in 12-month-old hg*FUS*^{R521C} and hg*FUS*^{R521H} spinal cords compared to age-matched Non-Tg and hg*FUS*^{WT}. eIF2α and tubulin antibodies were used as loading controls. See also quantification in Figures S6A and S6B.

(B) Expression of *Atf4* in primary hippocampal neuronal cultures from hg*FUS*^{R521H} compared to Non-Tg mice. Data are represented as mean ± SEM (n ≥ 3 biological replicates per group). *p < 0.05, two-sided unpaired Student's t test.

(C) Quantification of the amount of ^{35}S incorporated into newly translated proteins in hippocampal neurons cultured for 15 days. Cycloheximide (25 μg/mL) addition was used as a positive control for complete protein translation inhibition. Data are represented as mean ± SEM (n = 4 biological repeats). **p < 0.01, two-sided unpaired Student's t test.

(D) Schematic of the experimental design using microfluidic compartmented chambers to distinguish neuronal cell bodies from axons.

(E–G) Hippocampal neurons isolated from hg*FUS*^{R521H} or Non-Tg mice (from four independent experiments with 3 or 4 mouse embryos per genotype per experiment) cultured in compartmented chambers and treated with a 10-min pulse of puromycin to label protein translation sites using puromycin antibody (green) with a neurofilament NF-H (white) antibody to stain axons (E) or with an antibody recognizing the phosphorylated form of eIF2α (P-eIF2α) (red) in (F) cell bodies and (G) neuronal processes. Scale bars, 10 μm and 50 μm, respectively.

(H and I) Hippocampal neurons isolated from hg*FUS*^{R521H} and hg*FUS*^{WT} mice cultured in compartmented chambers immunostained for FUS (using FUS antibody in red) and protein translation (using puromycin antibody in green) in (H) cell bodies and (I) axonal processes (using NF-H antibody, white). Scale bar, 50 μm.

(J–M) Quantification of the puromycin relative fluorescence intensity (protein translation) in (J) cell bodies (green signal in Figure 6F) and (K) axons (green signal in Figure 6G); quantification of P-eiF2α fluorescence intensity (stress) in (L) cell bodies (red signal in Figure 6F) and (M) axons (red signal in Figure 6G) of Non-Tg and hg*FUS*^{R521H} hippocampal neurons cultured in microfluidic chambers. The bar graph represents mean ± SEM (n ≥ 30 axons or cell bodies per group from four independent experiments). ***p < 0.001, two-sided unpaired Student's t test.

(N and O) Quantification of FUS relative fluorescence intensity in (N) cell bodies (red signal in Figure 6H) and (O) axons (red signal in Figure 6I). The bar graph represents mean ± SEM (n ≥ 50 axons or cell bodies per group). ***p < 0.001, two-sided unpaired Student's t test.

those seen at end-stage disease in patients are not required for disease initiation, consistent with several other neurodegenerative diseases in which large inclusions correlate poorly with either onset or severity of neurodegeneration (Arnold et al., 2013; Arrasate et al., 2004; Kirkitadze et al., 2002; Parone et al., 2013).

Increasing evidence suggests that RNA-binding proteins including TDP-43, FMRP, SMN, and ataxin-2 are involved in

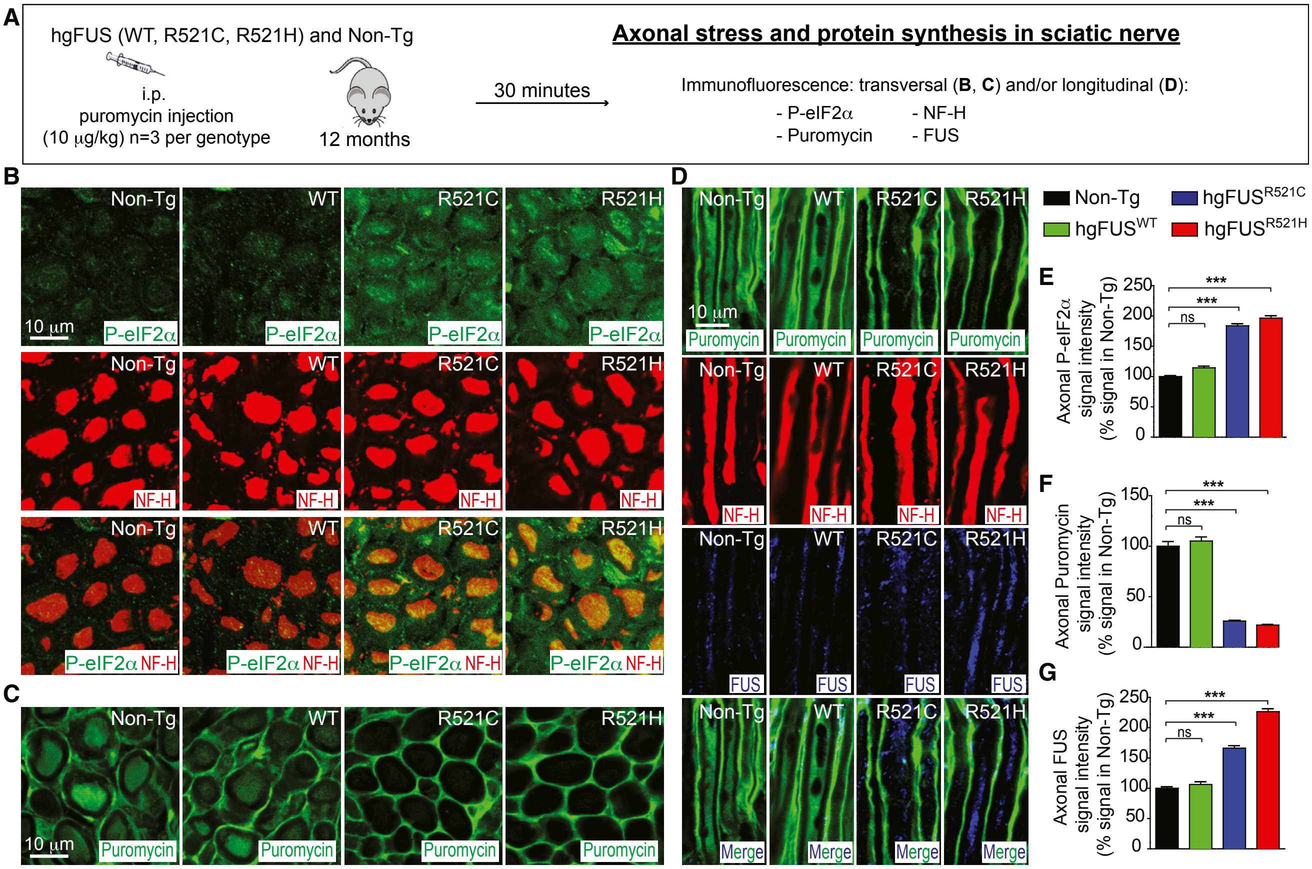

Figure 7. ALS-Linked Mutations in FUS Cause Stress-Mediated Reduction in Local Axonal Protein Synthesis within Sciatic Nerve Axons of Mutant FUS Mice

(A) Experimental design to assess stress and local axonal translation in the sciatic nerve of 12-month-old Non-Tg, hg*FUS*^{WT}, hg*FUS*^{R521C}, and hg*FUS*^{R521H} mice.

(B) Transversal sections of mouse sciatic nerves immunostained using P-eIF2α (green) and NF-H (red) antibodies and (C) puromycin (green) antibody.

(D) Longitudinal sections of mouse sciatic nerves immunostained using puromycin (green), NF-H (red), and FUS (blue) antibodies. Scale bars, 10 µm.

(E–G) Quantification of the relative fluorescence intensity of the (E) P-eIF2α, (F) puromycin, and (G) FUS immunostainings within sciatic nerve axons of 12-month-old Non-Tg, hg*FUS*^{WT}, hg*FUS*^{R521C}, and hg*FUS*^{R521H} mice. The bar graph represents mean ± SEM (n = 3 animals per group). ***$p < 0.001$, two-sided unpaired Student's t test.

the regulation of local protein translation and that local translation is critical for synaptic function (Donlin-Asp et al., 2017). FUS is present not only in soma but also along neuronal dendrites in RNA granules (Belly et al., 2005; Yasuda et al., 2013), spines (Fujii et al., 2005) in close proximity to presynaptic vesicles (Schoen et al., 2016), and at the neuromuscular junctions (So et al., 2018). To this, we now demonstrate that FUS protein is present along axons, including at sites of intra-axonal protein synthesis, and that the accumulation of ALS-linked mutants of FUS is significantly increased along axons and dendrites of hippocampal and sciatic nerve neurons.

FUS has been proposed to play a role at the synapse in spine formation (Fujii et al., 2005) and maturation (Udagawa et al., 2015) of hippocampal neurons, possibly by regulating local mRNA translation of synaptic components such as GluA1 (Udagawa et al., 2015) or RNAs enriched in cell protrusions (Yasuda et al., 2017; Yasuda et al., 2013). A role as a RNA chaperone suppressing RNA folding and repeat-associated translation of UGGAA expanded repeats has also been described in a fly model for ataxia (Ishiguro et al., 2017). While expression of high levels of FUS mutants was reported to provoke a reduction in protein synthesis in neuronal cultures (Murakami et al., 2015) and alter dendritic branching and spines in mice (Sephton et al., 2014), it is not established whether—and, if so, how—mutations in FUS may alter such critical functions. In flies, synaptic transmission at the neuromuscular junctions of *Drosophila* larvae expressing FUS mutants was significantly decreased (Shahidullah et al., 2013). Our efforts provide *in vivo* evidence in the mammalian nervous system that expression of FUS mutants at levels approximating the normal level of endogenous FUS is sufficient to cause stress-mediated reduction in intra-axonal protein synthesis prior to synaptic dysfunction and loss and is therefore likely to contribute to synaptic dysfunction associated with age-dependent motor and cognitive deficits.

Indeed, we have demonstrated FUS mutant-dependent suppression of expression of a wide range of components of the protein translation machinery, including mRNAs from the large

and small ribosomal subunits. Correspondingly, FUS may play a role in the translation of a wide range of genes. This contrasts with other RNA-binding proteins whose actions have recently been proposed to exert translational control on a specific subset of genes (examples include ataxin 2 on *RGS8* [Dansithong et al., 2015], FMRP on diacylglycerol kinase kappa [Tabet et al., 2016], or FMRP together with TDP-43 on *Rac1*, *Map1b*, and *GluR1* [Majumder et al., 2016]). Future efforts are now needed to identify the spectrum of axonal RNAs affected by FUS mutations. Added to this, mutations in FUS impair axonal transport, including transport of mitochondria and endoplasmic reticulum vesicles in patient-derived (Guo et al., 2017) and *Drosophila* (Baldwin et al., 2016) motor neurons. It is therefore possible that alterations in axonal transport of RNAs contribute to the reduction in local intra-axonal protein synthesis.

Finally, we find an early activation of the integrated stress response (ISR) pathway with increased phosphorylation of eIF2α (Pakos-Zebrucka et al., 2016; Zhao and Ackerman, 2006). Thus, we propose that ALS-linked mutations in FUS lead to increased accumulation of mutant FUS along axons, including at local intra-axonal translation sites; this in turn provokes local ISR that inhibits local protein synthesis, ultimately impairing neuronal synaptic function. Chemical ISR inhibitors have been reported to increase long-term memory (Sidrauski et al., 2013), reverse cognitive deficits associated with traumatic brain injury (Chou et al., 2017) in rodents and reduce TDP-43 mediated toxicity in flies and neuronal cultures (Kim et al., 2014a). It will now be of high interest to test whether modulating ISR pathways using similar compounds reduces the age-dependent motor and cognitive deficits associated with mutant FUS.

STAR★METHODS

Detailed methods are provided in the online version of this paper and include the following:

- KEY RESOURCES TABLE
- CONTACT FOR REAGENT AND RESOURCE SHARING
- EXPERIMENTAL MODEL AND SUBJECT DETAILS
  - Mice
  - Primary cultures
- METHOD DETAILS
  - Immunofluorescence
  - Morphometric Analysis of Axons
  - Motor neuron counting
  - Neuromuscular junction innervation
  - *In vivo* protein synthesis labeling
  - Immunoblotting
  - Antibodies
  - Nuclear-Cytosolic Fractionation
  - Sequential Biochemical Fractionation
  - Total-body mouse γ-irradiation
  - Cell viability assay
  - Multi-electrode array (MEA)
  - Compartmentalized microfluidic devices
  - Puromycin incorporation assay
  - [^{35}S]-Methionine Labeling/Radioactivity assay
  - RNA extraction and qPCR
  - RNA-seq
  - RASL-seq
  - Splicing gels
  - Mouse behavioral tests
- QUANTIFICATION AND STATISTICAL ANALYSIS
- DATA AND SOFTWARE AVAILABILITY

SUPPLEMENTAL INFORMATION

Supplemental Information includes six figures and two tables and can be found with this article online at https://doi.org/10.1016/j.neuron.2018.09.044.

A video abstract is available at https://doi.org/10.1016/j.neuron.2018.09.044#mmc4.

ACKNOWLEDGMENTS

We thank Amanda Roberts for her critical advice and expertise with the cognitive mouse behavioral assays at the Mouse Behavioral Assessment Core, The Scripps Research Institute, La Jolla. We thank Susan Ackerman (University of California at San Diego) for her critical expertise and suggestions. We are grateful to Ying Jones (University of California San Diego) for her technical assistance with plastic thin sectioning and staining of lumbar roots and to Jennifer Santini (University of California San Diego, Light Microscopy Core, NS047101) for resources. This work was supported by a Strategic Grant Award from The Wellcome Trust and Medical Research Council, Target ALS (grant to D.W.C.), and the Muscular Dystrophy Association (grant MDA352600 to D.W.C. and S.D.C.). J.L.-E. is the recipient of a Milton Safenowitz postdoctoral fellowship. M.W.B. receives salary support from the UCSD Genetics Training Program from the National Institute for General Medical Sciences, T32 GM008666. S.D.C. and D.W.C. receive salary support from the Ludwig Institute for Cancer Research. C.E.S. receives salary support from the Medical Research Council, Alzheimer Research UK, and Alzheimer Society through the United Kingdom Dementia Research Institute, as well as from both the National Institute for Health Research (NIHR) Dementia Biomedical Research Unit at South London and Maudsley NHS Foundation Trust and King's College London.

AUTHOR CONTRIBUTIONS

J.L.-E., T.T., P.A.P., M.M., C.L.-T., D.W.C., and S.D.C. designed the experiments and analyzed the data. J.L.-E., T.T., M.W.B., B.M., M.M.-D., C.C.-M., J.N.A., J.A., A.T.B., A.P.V., S.K.L., A.V.L., Y.S., M.J., J.B., D.S., J.Q., Z.O., S.-C.L., P.A.P., and S.D.C. performed the experiments or bioinformatical analyses. X.-D.F., L.T., S.-C.L., G.G.H., and C.E.S. contributed key reagents. J.L.-E., S.D.C., and D.W.C. wrote the manuscript.

DECLARATION OF INTERESTS

The authors declare no competing interests.

Received: January 15, 2018
Revised: May 11, 2018
Accepted: September 25, 2018
Published: October 18, 2018

REFERENCES

Ahmed, E.A., Vélaz, E., Rosemann, M., Gilbertz, K.P., and Scherthan, H. (2017). DNA repair kinetics in SCID mice Sertoli cells and DNA-PKcs-deficient mouse embryonic fibroblasts. Chromosoma *126*, 287–298.

Andersson, M.K., Ståhlberg, A., Arvidsson, Y., Olofsson, A., Semb, H., Stenman, G., Nilsson, O., and Aman, P. (2008). The multifunctional FUS, EWS and TAF15 proto-oncoproteins show cell type-specific expression patterns and involvement in cell spreading and stress response. BMC Cell Biol. *9*, 37.

Arnold, E.S., Ling, S.C., Huelga, S.C., Lagier-Tourenne, C., Polymenidou, M., Ditsworth, D., Kordasiewicz, H.B., McAlonis-Downes, M., Platoshyn, O., Parone, P.A., et al. (2013). ALS-linked TDP-43 mutations produce aberrant RNA splicing and adult-onset motor neuron disease without aggregation or loss of nuclear TDP-43. Proc. Natl. Acad. Sci. USA *110*, E736–E745.

Arrasate, M., Mitra, S., Schweitzer, E.S., Segal, M.R., and Finkbeiner, S. (2004). Inclusion body formation reduces levels of mutant huntingtin and the risk of neuronal death. Nature *431*, 805–810.

Aulas, A., and Vande Velde, C. (2015). Alterations in stress granule dynamics driven by TDP-43 and FUS: a link to pathological inclusions in ALS? Front. Cell. Neurosci. *9*, 423.

Baldwin, K.R., Godena, V.K., Hewitt, V.L., and Whitworth, A.J. (2016). Axonal transport defects are a common phenotype in Drosophila models of ALS. Hum. Mol. Genet. *25*, 2378–2392.

Belly, A., Moreau-Gachelin, F., Sadoul, R., and Goldberg, Y. (2005). Delocalization of the multifunctional RNA splicing factor TLS/FUS in hippocampal neurones: exclusion from the nucleus and accumulation in dendritic granules and spine heads. Neurosci. Lett. *379*, 152–157.

Belzil, V.V., Daoud, H., St-Onge, J., Desjarlais, A., Bouchard, J.P., Dupre, N., Lacomblez, L., Salachas, F., Pradat, P.F., Meininger, V., et al. (2011). Identification of novel FUS mutations in sporadic cases of amyotrophic lateral sclerosis. Amyotroph. Lateral Scler. *12*, 113–117.

Broustal, O., Camuzat, A., Guillot-Noël, L., Guy, N., Millecamps, S., Deffond, D., Lacomblez, L., Golfier, V., Hannequin, D., Salachas, F., et al.; French clinical and genetic research network on FTD/FTD-MND (2010). FUS mutations in frontotemporal lobar degeneration with amyotrophic lateral sclerosis. J. Alzheimers Dis. *22*, 765–769.

Chailangkarn, T., Trujillo, C.A., Freitas, B.C., Hrvoj-Mihic, B., Herai, R.H., Yu, D.X., Brown, T.T., Marchetto, M.C., Bardy, C., McHenry, L., et al. (2016). A human neurodevelopmental model for Williams syndrome. Nature *536*, 338–343.

Chio, A., Calvo, A., Moglia, C., Ossola, I., Brunetti, M., Sbaiz, L., Lai, S.L., Abramzon, Y., Traynor, B.J., and Restagno, G. (2011). A de novo missense mutation of the FUS gene in a "true" sporadic ALS case. Neurobiol. Aging *32*. 553.e23–26. Published online July 3, 2010.

Chou, A., Krukowski, K., Jopson, T., Zhu, P.J., Costa-Mattioli, M., Walter, P., and Rosi, S. (2017). Inhibition of the integrated stress response reverses cognitive deficits after traumatic brain injury. Proc. Natl. Acad. Sci. USA *114*, E6420–E6426.

Da Cruz, S., and Cleveland, D.W. (2011). Understanding the role of TDP-43 and FUS/TLS in ALS and beyond. Curr. Opin. Neurobiol. *21*, 904–919.

Dansithong, W., Paul, S., Figueroa, K.P., Rinehart, M.D., Wiest, S., Pflieger, L.T., Scoles, D.R., and Pulst, S.M. (2015). Ataxin-2 regulates RGS8 translation in a new BAC-SCA2 transgenic mouse model. PLoS Genet. *11*, e1005182.

DeJesus-Hernandez, M., Kocerha, J., Finch, N., Crook, R., Baker, M., Desaro, P., Johnston, A., Rutherford, N., Wojtas, A., Kennelly, K., et al. (2010). De novo truncating FUS gene mutation as a cause of sporadic amyotrophic lateral sclerosis. Hum. Mutat. *31*, E1377–E1389.

Devoy, A., Kalmar, B., Stewart, M., Park, H., Burke, B., Noy, S.J., Redhead, Y., Humphrey, J., Lo, K., Jaeger, J., et al. (2017). Humanized mutant FUS drives progressive motor neuron degeneration without aggregation in 'FUSDelta14' knockin mice. Brain *140*, 2797–2805.

Doi, H., Okamura, K., Bauer, P.O., Furukawa, Y., Shimizu, H., Kurosawa, M., Machida, Y., Miyazaki, H., Mitsui, K., Kuroiwa, Y., and Nukina, N. (2008). RNA-binding protein TLS is a major nuclear aggregate-interacting protein in huntingtin exon 1 with expanded polyglutamine-expressing cells. J. Biol. Chem. *283*, 6489–6500.

Donlin-Asp, P.G., Rossoll, W., and Bassell, G.J. (2017). Spatially and temporally regulating translation via mRNA-binding proteins in cellular and neuronal function. FEBS Lett. *591*, 1508–1525.

Donnelly, N., Gorman, A.M., Gupta, S., and Samali, A. (2013). The eIF2α kinases: their structures and functions. Cell. Mol. Life Sci. *70*, 3493–3511.

Dormann, D., Madl, T., Valori, C.F., Bentmann, E., Tahirovic, S., Abou-Ajram, C., Kremmer, E., Ansorge, O., Mackenzie, I.R., Neumann, M., and Haass, C. (2012). Arginine methylation next to the PY-NLS modulates Transportin binding and nuclear import of FUS. EMBO J. *31*, 4258–4275.

Ennaceur, A., and Delacour, J. (1988). A new one-trial test for neurobiological studies of memory in rats. 1: Behavioral data. Behav. Brain Res. *31*, 47–59.

Fujii, R., Okabe, S., Urushido, T., Inoue, K., Yoshimura, A., Tachibana, T., Nishikawa, T., Hicks, G.G., and Takumi, T. (2005). The RNA binding protein TLS is translocated to dendritic spines by mGluR5 activation and regulates spine morphology. Curr. Biol. *15*, 587–593.

Goodman, C.A., Mabrey, D.M., Frey, J.W., Miu, M.H., Schmidt, E.K., Pierre, P., and Hornberger, T.A. (2011). Novel insights into the regulation of skeletal muscle protein synthesis as revealed by a new nonradioactive in vivo technique. FASEB J. *25*, 1028–1039.

Guo, W., Naujock, M., Fumagalli, L., Vandoorne, T., Baatsen, P., Boon, R., Ordovás, L., Patel, A., Welters, M., Vanwelden, T., et al. (2017). HDAC6 inhibition reverses axonal transport defects in motor neurons derived from FUS-ALS patients. Nat. Commun. *8*, 861.

Heyser, C.J., and Chemero, A. (2012). Novel object exploration in mice: not all objects are created equal. Behav. Processes *89*, 232–238.

Hicks, G.G., Singh, N., Nashabi, A., Mai, S., Bozek, G., Klewes, L., Arapovic, D., White, E.K., Koury, M.J., Oltz, E.M., et al. (2000). Fus deficiency in mice results in defective B-lymphocyte development and activation, high levels of chromosomal instability and perinatal death. Nat. Genet. *24*, 175–179.

Holcomb, L.A., Gordon, M.N., Jantzen, P., Hsiao, K., Duff, K., and Morgan, D. (1999). Behavioral changes in transgenic mice expressing both amyloid precursor protein and presenilin-1 mutations: lack of association with amyloid deposits. Behav. Genet. *29*, 177–185.

Honda, D., Ishigaki, S., Iguchi, Y., Fujioka, Y., Udagawa, T., Masuda, A., Ohno, K., Katsuno, M., and Sobue, G. (2013). The ALS/FTLD-related RNA-binding proteins TDP-43 and FUS have common downstream RNA targets in cortical neurons. FEBS Open Bio *4*, 1–10.

Huang, C., Zhou, H., Tong, J., Chen, H., Liu, Y.J., Wang, D., Wei, X., and Xia, X.G. (2011). FUS transgenic rats develop the phenotypes of amyotrophic lateral sclerosis and frontotemporal lobar degeneration. PLoS Genet. *7*, e1002011.

Ichiyanagi, N., Fujimori, K., Yano, M., Ishihara-Fujisaki, C., Sone, T., Akiyama, T., Okada, Y., Akamatsu, W., Matsumoto, T., Ishikawa, M., et al. (2016). Establishment of In Vitro FUS-Associated Familial Amyotrophic Lateral Sclerosis Model Using Human Induced Pluripotent Stem Cells. Stem Cell Reports *6*, 496–510.

Ishiguro, T., Sato, N., Ueyama, M., Fujikake, N., Sellier, C., Kanegami, A., Tokuda, E., Zamiri, B., Gall-Duncan, T., Mirceta, M., et al. (2017). Regulatory Role of RNA Chaperone TDP-43 for RNA Misfolding and Repeat-Associated Translation in SCA31. Neuron *94*, 108–124.e107.

Jackson, E., Demarest, K., Eckert, W.J., Cates-Gatto, C., Nadav, T., Cates, L.N., Howard, H., and Roberts, A.J. (2015). Aspen shaving versus chip bedding: effects on breeding and behavior. Lab. Anim. *49*, 46–56.

Kapeli, K., Pratt, G.A., Vu, A.Q., Hutt, K.R., Martinez, F.J., Sundararaman, B., Batra, R., Freese, P., Lambert, N.J., Huelga, S.C., et al. (2016). Distinct and shared functions of ALS-associated proteins TDP-43, FUS and TAF15 revealed by multisystem analyses. Nat. Commun. *7*, 12143.

Kapur, M., Monaghan, C.E., and Ackerman, S.L. (2017). Regulation of mRNA Translation in Neurons-A Matter of Life and Death. Neuron *96*, 616–637.

Kenney, J.W., and Gould, T.J. (2008). Modulation of hippocampus-dependent learning and synaptic plasticity by nicotine. Mol. Neurobiol. *38*, 101–121.

Khoutorsky, A., Bonin, R.P., Sorge, R.E., Gkogkas, C.G., Pawlowski, S.A., Jafarnejad, S.M., Pitcher, M.H., Alain, T., Perez-Sanchez, J., Salter, E.W., et al. (2015). Translational control of nociception via 4E-binding protein 1. eLife *4*, e12002.

Kim, H.J., Raphael, A.R., LaDow, E.S., McGurk, L., Weber, R.A., Trojanowski, J.Q., Lee, V.M., Finkbeiner, S., Gitler, A.D., and Bonini, N.M. (2014a). Therapeutic modulation of eIF2α phosphorylation rescues TDP-43 toxicity in amyotrophic lateral sclerosis disease models. Nat. Genet. *46*, 152–160.

Kim, Y.E., Oh, K.W., Kwon, M.J., Choi, W.J., Oh, S.I., Ki, C.S., and Kim, S.H. (2014b). De novo FUS mutations in 2 Korean patients with sporadic amyotrophic lateral sclerosis. Neurobiol. Aging *36*, 1604.e17–e19.

King, A., Troakes, C., Smith, B., Nolan, M., Curran, O., Vance, C., Shaw, C.E., and Al-Sarraj, S. (2015). ALS-FUS pathology revisited: singleton FUS mutations and an unusual case with both a FUS and TARDBP mutation. Acta Neuropathol. Commun. *3*, 62.

Kirkitadze, M.D., Bitan, G., and Teplow, D.B. (2002). Paradigm shifts in Alzheimer's disease and other neurodegenerative disorders: the emerging role of oligomeric assemblies. J. Neurosci. Res. *69*, 567–577.

Koch, A., Gulani, J., King, G., Hieber, K., Chappell, M., and Ossetrova, N. (2016). Establishment of Early Endpoints in Mouse Total-Body Irradiation Model. PLoS ONE *11*, e0161079.

Kuroda, M., Sok, J., Webb, L., Baechtold, H., Urano, F., Yin, Y., Chung, P., de Rooij, D.G., Akhmedov, A., Ashley, T., and Ron, D. (2000). Male sterility and enhanced radiation sensitivity in TLS(-/-) mice. EMBO J. *19*, 453–462.

Kwiatkowski, T.J., Jr., Bosco, D.A., Leclerc, A.L., Tamrazian, E., Vanderburg, C.R., Russ, C., Davis, A., Gilchrist, J., Kasarskis, E.J., Munsat, T., et al. (2009). Mutations in the FUS/TLS gene on chromosome 16 cause familial amyotrophic lateral sclerosis. Science *323*, 1205–1208.

Lagier-Tourenne, C., Polymenidou, M., and Cleveland, D.W. (2010). TDP-43 and FUS/TLS: emerging roles in RNA processing and neurodegeneration. Hum. Mol. Genet. *19* (R1), R46–R64.

Lagier-Tourenne, C., Polymenidou, M., Hutt, K.R., Vu, A.Q., Baughn, M., Huelga, S.C., Clutario, K.M., Ling, S.C., Liang, T.Y., Mazur, C., et al. (2012). Divergent roles of ALS-linked proteins FUS/TLS and TDP-43 intersect in processing long pre-mRNAs. Nat. Neurosci. *15*, 1488–1497.

Lalonde, R. (2002). The neurobiological basis of spontaneous alternation. Neurosci. Biobehav. Rev. *26*, 91–104.

Lieben, C.K., Steinbusch, H.W., and Blokland, A. (2006). 5,7-DHT lesion of the dorsal raphe nuclei impairs object recognition but not affective behavior and corticosterone response to stressor in the rat. Behav. Brain Res. *168*, 197–207.

Mackenzie, I.R., Neumann, M., Bigio, E.H., Cairns, N.J., Alafuzoff, I., Kril, J., Kovacs, G.G., Ghetti, B., Halliday, G., Holm, I.E., et al. (2010). Nomenclature and nosology for neuropathologic subtypes of frontotemporal lobar degeneration: an update. Acta Neuropathol. *119*, 1–4.

Mackenzie, I.R., Ansorge, O., Strong, M., Bilbao, J., Zinman, L., Ang, L.C., Baker, M., Stewart, H., Eisen, A., Rademakers, R., and Neumann, M. (2011). Pathological heterogeneity in amyotrophic lateral sclerosis with FUS mutations: two distinct patterns correlating with disease severity and mutation. Acta Neuropathol. *122*, 87–98.

Majumder, P., Chu, J.F., Chatterjee, B., Swamy, K.B., and Shen, C.J. (2016). Co-regulation of mRNA translation by TDP-43 and Fragile X Syndrome protein FMRP. Acta Neuropathol. *132*, 721–738.

March, Z.M., King, O.D., and Shorter, J. (2016). Prion-like domains as epigenetic regulators, scaffolds for subcellular organization, and drivers of neurodegenerative disease. Brain Res. *1647*, 9–18.

Maren, S. (2001). Neurobiology of Pavlovian fear conditioning. Annu. Rev. Neurosci. *24*, 897–931.

Mitchell, J.C., McGoldrick, P., Vance, C., Hortobagyi, T., Sreedharan, J., Rogelj, B., Tudor, E.L., Smith, B.N., Klasen, C., Miller, C.C., et al. (2013). Overexpression of human wild-type FUS causes progressive motor neuron degeneration in an age- and dose-dependent fashion. Acta Neuropathol. *125*, 273–288.

Moy, S.S., Nadler, J.J., Perez, A., Barbaro, R.P., Johns, J.M., Magnuson, T.R., Piven, J., and Crawley, J.N. (2004). Sociability and preference for social novelty in five inbred strains: an approach to assess autistic-like behavior in mice. Genes Brain Behav. *3*, 287–302.

Moy, S.S., Nadler, J.J., Young, N.B., Perez, A., Holloway, L.P., Barbaro, R.P., Barbaro, J.R., Wilson, L.M., Threadgill, D.W., Lauder, J.M., et al. (2007). Mouse behavioral tasks relevant to autism: phenotypes of 10 inbred strains. Behav. Brain Res. *176*, 4–20.

Mumby, D.G., Tremblay, A., Lecluse, V., and Lehmann, H. (2005). Hippocampal damage and anterograde object-recognition in rats after long retention intervals. Hippocampus *15*, 1050–1056.

Munoz, D.G., Neumann, M., Kusaka, H., Yokota, O., Ishihara, K., Terada, S., Kuroda, S., and Mackenzie, I.R. (2009). FUS pathology in basophilic inclusion body disease. Acta Neuropathol. *118*, 617–627.

Murakami, T., Qamar, S., Lin, J.Q., Schierle, G.S., Rees, E., Miyashita, A., Costa, A.R., Dodd, R.B., Chan, F.T., Michel, C.H., et al. (2015). ALS/FTD Mutation-Induced Phase Transition of FUS Liquid Droplets and Reversible Hydrogels into Irreversible Hydrogels Impairs RNP Granule Function. Neuron *88*, 678–690.

Neumann, M., Rademakers, R., Roeber, S., Baker, M., Kretzschmar, H.A., and Mackenzie, I.R. (2009). A new subtype of frontotemporal lobar degeneration with FUS pathology. Brain *132*, 2922–2931.

Niederst, E.D., Reyna, S.M., and Goldstein, L.S. (2015). Axonal amyloid precursor protein and its fragments undergo somatodendritic endocytosis and processing. Mol. Biol. Cell *26*, 205–217.

Nussbacher, J.K., Batra, R., Lagier-Tourenne, C., and Yeo, G.W. (2015). RNA-binding proteins in neurodegeneration: Seq and you shall receive. Trends Neurosci. *38*, 226–236.

Pakos-Zebrucka, K., Koryga, I., Mnich, K., Ljujic, M., Samali, A., and Gorman, A.M. (2016). The integrated stress response. EMBO Rep. *17*, 1374–1395.

Parone, P.A., Da Cruz, S., Han, J.S., McAlonis-Downes, M., Vetto, A.P., Lee, S.K., Tseng, E., and Cleveland, D.W. (2013). Enhancing mitochondrial calcium buffering capacity reduces aggregation of misfolded SOD1 and motor neuron cell death without extending survival in mouse models of inherited amyotrophic lateral sclerosis. J. Neurosci. *33*, 4657–4671.

Pennanen, L., Wolfer, D.P., Nitsch, R.M., and Götz, J. (2006). Impaired spatial reference memory and increased exploratory behavior in P301L tau transgenic mice. Genes Brain Behav. *5*, 369–379.

Qiu, H., Lee, S., Shang, Y., Wang, W.Y., Au, K.F., Kamiya, S., Barmada, S.J., Finkbeiner, S., Lui, H., Carlton, C.E., et al. (2014). ALS-associated mutation FUS-R521C causes DNA damage and RNA splicing defects. J. Clin. Invest. *124*, 981–999.

Rademakers, R., Stewart, H., Dejesus-Hernandez, M., Krieger, C., Graff-Radford, N., Fabros, M., Briemberg, H., Cashman, N., Eisen, A., and Mackenzie, I.R. (2010). Fus gene mutations in familial and sporadic amyotrophic lateral sclerosis. Muscle Nerve *42*, 170–176.

Reber, S., Stettler, J., Filosa, G., Colombo, M., Jutzi, D., Lenzken, S.C., Schweingruber, C., Bruggmann, R., Bachi, A., Barabino, S.M., et al. (2016). Minor intron splicing is regulated by FUS and affected by ALS-associated FUS mutants. EMBO J. *35*, 1504–1521.

Rudy, J.W., Huff, N.C., and Matus-Amat, P. (2004). Understanding contextual fear conditioning: insights from a two-process model. Neurosci. Biobehav. Rev. *28*, 675–685.

Sabatelli, M., Moncada, A., Conte, A., Lattante, S., Marangi, G., Luigetti, M., Lucchini, M., Mirabella, M., Romano, A., Del Grande, A., et al. (2013). Mutations in the 3′ untranslated region of FUS causing FUS overexpression are associated with amyotrophic lateral sclerosis. Hum. Mol. Genet. *22*, 4748–4755.

Scekic-Zahirovic, J., Sendscheid, O., El Oussini, H., Jambeau, M., Sun, Y., Mersmann, S., Wagner, M., Dieterlé, S., Sinniger, J., Dirrig-Grosch, S., et al. (2016). Toxic gain of function from mutant FUS protein is crucial to trigger cell autonomous motor neuron loss. EMBO J. *35*, 1077–1097.

Scekic-Zahirovic, J., Oussini, H.E., Mersmann, S., Drenner, K., Wagner, M., Sun, Y., Allmeroth, K., Dieterlé, S., Sinniger, J., Dirrig-Grosch, S., et al. (2017). Motor neuron intrinsic and extrinsic mechanisms contribute to the pathogenesis of FUS-associated amyotrophic lateral sclerosis. Acta Neuropathol. *133*, 887–906.

Schoen, M., Reichel, J.M., Demestre, M., Putz, S., Deshpande, D., Proepper, C., Liebau, S., Schmeisser, M.J., Ludolph, A.C., Michaelis, J., and Boeckers, T.M. (2016). Super-Resolution Microscopy Reveals Presynaptic Localization

of the ALS/FTD Related Protein FUS in Hippocampal Neurons. Front. Cell. Neurosci. *9*, 496.

Sephton, C.F., Tang, A.A., Kulkarni, A., West, J., Brooks, M., Stubblefield, J.J., Liu, Y., Zhang, M.Q., Green, C.B., Huber, K.M., et al. (2014). Activity-dependent FUS dysregulation disrupts synaptic homeostasis. Proc. Natl. Acad. Sci. USA *111*, E4769–E4778.

Shahidullah, M., Le Marchand, S.J., Fei, H., Zhang, J., Pandey, U.B., Dalva, M.B., Pasinelli, P., and Levitan, I.B. (2013). Defects in synapse structure and function precede motor neuron degeneration in Drosophila models of FUS-related ALS. J. Neurosci. *33*, 19590–19598.

Sharma, A., Lyashchenko, A.K., Lu, L., Nasrabady, S.E., Elmaleh, M., Mendelsohn, M., Nemes, A., Tapia, J.C., Mentis, G.Z., and Shneider, N.A. (2016). ALS-associated mutant FUS induces selective motor neuron degeneration through toxic gain of function. Nat. Commun. *7*, 10465.

Shelkovnikova, T.A., Peters, O.M., Deykin, A.V., Connor-Robson, N., Robinson, H., Ustyugov, A.A., Bachurin, S.O., Ermolkevich, T.G., Goldman, I.L., Sadchikova, E.R., et al. (2013). Fused in sarcoma (FUS) protein lacking nuclear localization signal (NLS) and major RNA binding motifs triggers proteinopathy and severe motor phenotype in transgenic mice. J. Biol. Chem. *288*, 25266–25274.

Shiihashi, G., Ito, D., Yagi, T., Nihei, Y., Ebine, T., and Suzuki, N. (2016). Mislocated FUS is sufficient for gain-of-toxic-function amyotrophic lateral sclerosis phenotypes in mice. Brain *139*, 2380–2394.

Sidrauski, C., Acosta-Alvear, D., Khoutorsky, A., Vedantham, P., Hearn, B.R., Li, H., Gamache, K., Gallagher, C.M., Ang, K.K., Wilson, C., et al. (2013). Pharmacological brake-release of mRNA translation enhances cognitive memory. eLife *2*, e00498.

Silverman, J.L., Yang, M., Lord, C., and Crawley, J.N. (2010). Behavioural phenotyping assays for mouse models of autism. Nat. Rev. Neurosci. *11*, 490–502.

So, E., Mitchell, J.C., Memmi, C., Chennell, G., Vizcay-Barrena, G., Allison, L., Shaw, C.E., and Vance, C. (2018). Mitochondrial abnormalities and disruption of the neuromuscular junction precede the clinical phenotype and motor neuron loss in hFUSWT transgenic mice. Hum. Mol. Genet. *27*, 463–474.

Sun, S., Ling, S.C., Qiu, J., Albuquerque, C.P., Zhou, Y., Tokunaga, S., Li, H., Qiu, H., Bui, A., Yeo, G.W., et al. (2015). ALS-causative mutations in FUS/TLS confer gain and loss of function by altered association with SMN and U1-snRNP. Nat. Commun. *6*, 6171.

Tabet, R., Moutin, E., Becker, J.A., Heintz, D., Fouillen, L., Flatter, E., Krężel, W., Alunni, V., Koebel, P., Dembélé, D., et al. (2016). Fragile X Mental Retardation Protein (FMRP) controls diacylglycerol kinase activity in neurons. Proc. Natl. Acad. Sci. USA *113*, E3619–E3628.

Taylor, A.M., Blurton-Jones, M., Rhee, S.W., Cribbs, D.H., Cotman, C.W., and Jeon, N.L. (2005). A microfluidic culture platform for CNS axonal injury, regeneration and transport. Nat. Methods *2*, 599–605.

tom Dieck, S., Kochen, L., Hanus, C., Heumüller, M., Bartnik, I., Nassim-Assir, B., Merk, K., Mosler, T., Garg, S., Bunse, S., et al. (2015). Direct visualization of newly synthesized target proteins in situ. Nat. Methods *12*, 411–414.

Trapnell, C., Pachter, L., and Salzberg, S.L. (2009). TopHat: discovering splice junctions with RNA-Seq. Bioinformatics *25*, 1105–1111.

Trapnell, C., Williams, B.A., Pertea, G., Mortazavi, A., Kwan, G., van Baren, M.J., Salzberg, S.L., Wold, B.J., and Pachter, L. (2010). Transcript assembly and quantification by RNA-Seq reveals unannotated transcripts and isoform switching during cell differentiation. Nat. Biotechnol. *28*, 511–515.

Trapnell, C., Hendrickson, D.G., Sauvageau, M., Goff, L., Rinn, J.L., and Pachter, L. (2013). Differential analysis of gene regulation at transcript resolution with RNA-seq. Nat. Biotechnol. *31*, 46–53.

Udagawa, T., Fujioka, Y., Tanaka, M., Honda, D., Yokoi, S., Riku, Y., Ibi, D., Nagai, T., Yamada, K., Watanabe, H., et al. (2015). FUS regulates AMPA receptor function and FTLD/ALS-associated behaviour via GluA1 mRNA stabilization. Nat. Commun. *6*, 7098.

Van Langenhove, T., van der Zee, J., Sleegers, K., Engelborghs, S., Vandenberghe, R., Gijselinck, I., Van den Broeck, M., Mattheijssens, M., Peeters, K., De Deyn, P.P., et al. (2010). Genetic contribution of FUS to frontotemporal lobar degeneration. Neurology *74*, 366–371.

Vance, C., Rogelj, B., Hortobágyi, T., De Vos, K.J., Nishimura, A.L., Sreedharan, J., Hu, X., Smith, B., Ruddy, D., Wright, P., et al. (2009). Mutations in FUS, an RNA processing protein, cause familial amyotrophic lateral sclerosis type 6. Science *323*, 1208–1211.

Verbeeck, C., Deng, Q., Dejesus-Hernandez, M., Taylor, G., Ceballos-Diaz, C., Kocerha, J., Golde, T., Das, P., Rademakers, R., Dickson, D.W., and Kukar, T. (2012). Expression of Fused in sarcoma mutations in mice recapitulates the neuropathology of FUS proteinopathies and provides insight into disease pathogenesis. Mol. Neurodegener. *7*, 53.

Winters, B.D., Forwood, S.E., Cowell, R.A., Saksida, L.M., and Bussey, T.J. (2004). Double dissociation between the effects of peri-postrhinal cortex and hippocampal lesions on tests of object recognition and spatial memory: heterogeneity of function within the temporal lobe. J. Neurosci. *24*, 5901–5908.

Yan, J., Deng, H.X., Siddique, N., Fecto, F., Chen, W., Yang, Y., Liu, E., Donkervoort, S., Zheng, J.G., Shi, Y., et al. (2010). Frameshift and novel mutations in FUS in familial amyotrophic lateral sclerosis and ALS/dementia. Neurology *75*, 807–814.

Yasuda, K., Zhang, H., Loiselle, D., Haystead, T., Macara, I.G., and Mili, S. (2013). The RNA-binding protein Fus directs translation of localized mRNAs in APC-RNP granules. J. Cell Biol. *203*, 737–746.

Yasuda, K., Clatterbuck-Soper, S.F., Jackrel, M.E., Shorter, J., and Mili, S. (2017). FUS inclusions disrupt RNA localization by sequestering kinesin-1 and inhibiting microtubule detyrosination. J. Cell Biol. *216*, 1015–1034.

Zhao, L., and Ackerman, S.L. (2006). Endoplasmic reticulum stress in health and disease. Curr. Opin. Cell Biol. *18*, 444–452.

Zhou, Z., Qiu, J., Liu, W., Zhou, Y., Plocinik, R.M., Li, H., Hu, Q., Ghosh, G., Adams, J.A., Rosenfeld, M.G., and Fu, X.D. (2012). The Akt-SRPK-SR axis constitutes a major pathway in transducing EGF signaling to regulate alternative splicing in the nucleus. Mol. Cell *47*, 422–433.

STAR★METHODS

KEY RESOURCES TABLE

REAGENT OR RESOURCE	SOURCE	IDENTIFIER
Antibodies		
Rabbit anti-phosphorylated eIF2α (Ser51)	Cell Signaling Technology	Cat#9721; RRID: AB_330951
Rabbit anti- eIF2α	Cell Signaling Technology	Cat# 9722; RRID: AB_2230924
Mouse anti-FUS (4H11)	Santa Cruz Biotechnology	Cat# sc-47711; RRID: AB_2105208
Rabbit anti-FUS	Bethyl	Cat# A300-302A; RRID: AB_309445
Goat anti-FUS	Bethyl	Cat# A303-839A; RRID: AB_2620190
Rabbit anti-FUS	Proteintech Group	Cat#11570-1-AP; RRID: AB_2247082
Mouse anti-GAPDH (6C5)	Abcam	Cat# ab8245; RRID: AB_2107448
Mouse anti-Glial Fibrillary Acidic Protein (GFAP)	Millipore	Cat# MAB360; RRID: AB_11212597
Rabbit anti-HSF1	Cell Signaling Technology	Cat#4356S; RRID: AB_10695463
Rabbit anti-Hsp90 (C45G5)	Cell Signaling Technology	Cat#4877S; RRID: AB_2233307
Rabbit anti-Iba1	Wako	Cat#019-19741; RRID: AB_839504
Chicken anti-Map2	Novus	Cat# NB300-213; RRID: AB_2138178
Mouse anti-Neurofilament Clone RT97	Millipore	Cat# MAB5262; RRID: AB_95186
Rabbit anti-neurofilament H	Millipore	Cat# ab1989; RRID: AB_91202
Mouse anti-Neurofilament H non-phosphorylated SMI-32	Covance	Cat# SMI-32R-100; RRID: AB_509997
Mouse anti-Nucleophosmin	Zymed-Thermo Fisher Scientific	Cat#32-5200; RRID: AB_2533084
Chicken Neurofilament H	Millipore	Cat# AB5539; RRID: AB_11212161
Chicken anti-NeuN	Millipore	Cat# ABN91; RRID: AB_11205760
Anti-puromycin, clone 12D10	Millipore	Cat# MABE343; RRID: AB_2566826
Anti-puromycin, Alexa Fluor ® 488 Conjugated Antibody	Millipore	Cat# MABE343-AF488; RRID: AB_2736875
Rabbit anti-synapsin-1	Synaptic Systems	Cat#106103
Mouse anti-γH2A.X	Millipore	Cat# 05-636; RRID: AB_309864
Rabbit anti-53BP1	Novus	Cat# NB100-304; RRID: AB_10003037
Rabbit anti-calnexin	Enzo Life Sciences	Cat# SPA-860
Mouse anti-tubulin (clone DM1A)	This paper	N/A
Rabbit anti-human FUS #14080	This paper	N/A
Rabbit anti-mouse FUS #14082	This paper	N/A
Chemicals, Peptides, and Recombinant Proteins		
Sylgard 182 Elastomer	Fisher Scientific	Cat#NC9897184
Puromycin	Thermo Fisher Scientific	Cat# A1113803
Critical Commercial Assays		
LIVE/DEAD Cell viability assay	Thermo Fisher Scientific	Cat#L3224

(*Continued on next page*)

Continued

REAGENT OR RESOURCE	SOURCE	IDENTIFIER
Deposited Data		
RNaseq data	This paper	GEO: GSE120247
Experimental Models: Organisms/Strains		
Mouse: C57BL/6 hg*FUS*^{WT}	This paper	N/A
Mouse: C57BL/6 hg*FUS*^{R521C}	This paper	N/A
Mouse: C57BL/6 hg*FUS*^{R521H}	This paper	N/A
Mouse: C57BL/6 m*Fus*$^{-/-}$	Hicks et al., 2000	N/A
Oligonucleotides		
See Table S2 for primer sequences used in this paper	IDT	N/A
Software and Algorithms		
Fiji	NIH	https://fiji.sc/download; Fiji, RRID: SCR_002285
GraphPad Prism	GraphPad software	https://www.graphpad.com; GraphPad Prism, RRID: SCR_002798
AxIS (Biosystems Neural Metrics/Offline data analysis tool)	Axion Biosystems	https://www.axionbiosystems.com/products/axis-software; AxIS, RRID:SCR_016308
Bioquant Software	BIOQUANT Life Science,	https://lifescience.bioquant.com/; Bioquant Software RRID:SCR_016423
Other		
Compartmentalized microfluidic chambers	This paper	N/A

CONTACT FOR REAGENT AND RESOURCE SHARING

Further information and requests for resources and reagents should be directed to and will be fulfilled by the Lead Contact, Don W. Cleveland (dcleveland@uscd.edu).

EXPERIMENTAL MODEL AND SUBJECT DETAILS

Mice

Generation of hgFUS BAC transgenic mice

The human BAC construct expressing the full length *FUS* gene (18.2Kb) was obtained using DNA recombineering from the RP11-157F22 BAC clone. The sequences of the 5′ and 3′ homology arms used to retrieve the human *FUS* gene were: CCCCATAGCTGGG CAAATTTAGGCCAACACTC and CTAAGCGTGGTGGCGGGCGCCTGTAG. The ALS-linked mutations (R521C and R521H) were introduced using site-specific mutagenesis. Each construct was flanked by LoxP sites to further allow Cre-mediated excision of the human *FUS* gene in cell of interest. No other gene is on the BAC. The modified constructs (FUS wild-type or harboring either the R521H or R521C mutation) was injected into the pronuclei of fertilized C57BL/6 eggs and implanted into pseudo-pregnant female mice. All the mice used in this report were maintained on a pure C57BL/6 background. All experimental procedures were approved by the Institutional Animal Care and Use Committee of the University of California, San Diego.

Primary cultures

Primary hippocampal neuron cultures

Primary hippocampal neurons were prepared from embryonic day 16-17 (E16-E17). Hippocampal tissues were treated with 0.25% of Trysin+EDTA for 15 min at 37°C. Trypsin activity was inhibited using DMEM:F12 media supplemented with 10% of FBS and 1% of Penicillin/Streptomycin and tissues were then mechanically disaggregated using 1000 μL tips. A 4% BSA (in PBS1x) solution was further added to the cell suspension and centrifuged for 10 min at 1000rpm. The cell pellet was resuspended in Neurobasal media supplemented with 2% of B27, 200mM of L-glutamine and 100U/mL of penicillin-streptomycin. Cells were seeded in culture plates previously coated with poly-D-lysine at the concentration of 600,000 cells/mL.

METHOD DETAILS

Immunofluorescence

Mice were perfused intracardially and fixed with 4% paraformaldehyde in 0.1 M Sorenson's phosphate buffer, pH 7.2, the entire spinal cord was dissected, post-fixed for 2 hours in the same fixative and transferred in a 30% sucrose phosphate buffer for at least 2 days. The lumbar spinal cord or brain was embedded in OCT compound (Sakura) and snap frozen in isopentane (2-methylbutane) cooled at -40°C on dry ice. Floating lumbar spinal cord or brain cryosections (30μm or 35μm, respectively) were incubated in a blocking solution containing PBS1x, 0.5% Tween-20, 1.5% BSA for 1.5 hours at room temperature and then in PBS1x, 0.3% Triton X-100 overnight at room temperature with the primary antibodies (listed in antibodies section). Primary antibodies were washed with PBS1x and then detected using donkey anti-rabbit or anti-mouse FITC or Cy3 (1:500) coupled secondary antibodies (Jackson ImmunoResearch). The sections were washed with PBS1x and mounted. Analysis was performed on a Nikon Eclipse laser scanning confocal microscope. Fluorescence intensity from unsaturated images captured with identical confocal settings (minimum of four spinal sections were imaged per animal) was quantified using NIS elements software (Nikon).

Morphometric Analysis of Axons

Mice were perfused intracardially and fixed with 4% paraformaldehyde in 0.1 M Sorenson's phosphate buffer, pH 7.2, and the L5 lumbar roots were dissected and conserved in the same fixative at 4°C. L5 roots were embedded in Epon-Araldite as described in the electron microscopy section, thick sections (0.75μm) were prepared and stained for light microscopy with toluidine blue. Cross sections of L5 motor axons were analyzed at each age group. Axonal diameters were measured using the Bioquant Software and the number of large caliber axons with diameters over 4.5μm (α motor axons) was determined.

Motor neuron counting

ChAT positive ventral horn motor neurons were counted from 25-35 lumbar spinal cord cryosections (per animal) spaced 360μm apart and expressed as the average of total motor neurons counted divided by the number of sections.

Neuromuscular junction innervation

Gastrocnemius muscle was dissected from perfused mice and prepared as described in the immunofluorescence section. Floating 40 μm thick longitudinal sections of gastrocnemius were incubated in a blocking solution containing PBS1x, 0.5% Tween-20, 1.5% BSA for 4 hours at room temperature and then in PBS1x, 0.3% Triton X-100 overnight at room temperature with the polyclonal rabbit anti-synaptophysin antibody at 1:50 (Invitrogen). The sections were washed with PBS1x and then incubated first with donkey anti-rabbit Cy3 (Jackson ImmunoResearch) and α-bungarotoxin-Alexa488 (Invitrogen) at 1:500 for 1 hour at room temperature and then with Fluoromyelin red (Invitrogen) at 1:300 for 30 min. The sections were further washed with PBS1x and mounted. Analysis was performed on a Nikon Eclipse laser scanning confocal microscope. A total of approximately 1,000 neuromuscular junctions were counted from at least 10 sections of gastrocnemius. Individual NMJs were considered as innervated when synaptophysin staining covered at least 50% of the area of α-bungarotoxin staining.

In vivo protein synthesis labeling

Intraperitoneal injection of puromycin (10μg/kg) was performed in 12-month-old mice. Exactly 30 min post-injection (Goodman et al., 2011; Khoutorsky et al., 2015), mice were deeply anesthetized and perfused and fixed with 4% paraformaldehyde in 0.1 M Sorenson's phosphate buffer, pH 7.2. Sciatic nerves were dissected and post-fixed for 3 hours at 4°C, transferred in a 30% sucrose phosphate buffer and crypreserved. 30 μm thick longitudinal and transversal sections of sciatic nerves were processed for immunofluorescence, as described above.

Immunoblotting

Spinal cords from Non-Tg or hg*FUS* mice were homogenized in cold PBS1x supplemented with protease inhibitors. The lysates were centrifuged for 10 min at 1,000 x g and the resulting supernatants (clarified tissue extract) were analyzed by immunoblotting. Equal protein amounts were separated on SDS-PAGE, transferred to nitrocellulose membranes and probed with the indicated antibodies followed by horseradish peroxidase-conjugated secondary antibodies (Jackson ImmunoResearch). Pico or Femto ECL (Pierce) was used to detect immunoreactive bands.

Antibodies

Rabbit anti-phosphorylated eIF2α (Ser51) (1:100; Cell Signaling; #9721S); Rabbit anti-eiF2a (1:1000; Cell Signaling; #9722S); Mouse anti-FUS (4H11) (1:1000; Santa Cruz, sc-47711); Rabbit anti-FUS (1:500; Bethyl, A300-302A); Goat anti-FUS (1:500; Bethyl, A303-839A); Rabbit anti-FUS (1:500; Proteintech 11570-1-AP); Mouse anti-GAPDH (6C5) (1:10,000; Abcam, ab8245); Mouse anti-GFAP (clone G-A-5) (1:500; Millipore, MAB360); Rabbit anti-HSF1 (1:100; Cell Signaling, 4356S); Rabbit anti-Hsp90 (C45G5) (1:1000; Cell Signaling, 4877S); Rabbit anti-Iba1 (1:500; Wako, 019-19741); Chicken anti-Map2 (1:500; Novus Biological; NB300-213); Mouse anti-Neurofilament Clone RT97 (1:500; Millipore, MAB5262); Rabbit anti-neurofilament H (1:500; Millipore, ab1989); Mouse anti-Neurofilament H non-phosphorylated SMI-32 (1:500; Covance, SMI32R); Mouse anti-Nucleophosmin (1:1000; Zymed, 32-5200);

Chicken Neurofilament H (1:1000, Millipore, AB5539); Chicken anti-NeuN (1:1,000, Millipore, ABN91); Mouse anti-puromycin (1:100; Millipore, MABE343); Conjugated Alexa 488 anti-puromycin (1:100; Millipore, MABE343-AF488); Rabbit anti-synapsin-1 (1:500 IF; 1:1000 WB; SYSY synaptic systems, 106103); Mouse anti-γH2A.X (1:5000; Millipore 05-636); Rabbit anti-53BP1 (1:5000; Novus Biological NB100-304); Rabbit anti-calnexin (1:1,000; Enzo, SPA-860); Mouse anti-tubulin (DM1A clone) (1:10,000, home-made); Rabbit anti-human FUS #14080 (1:25000; home-made) and anti-mouse FUS #14082 (1:4000, home-made).

Nuclear-Cytosolic Fractionation

Spinal cords were dissected, weighed, and fresh tissue was gently lysed in 10x (vol/wt) hypotonic buffer A (10 mM HEPES-KOH pH 7.4, 10 mM KCl, 1.5 mM MgCl, 0.5 mM EDTA, 0.5 mM EGTA), 1x protease inhibitors (Roche) using a pre-chilled glass dounce homogenizer (tight fit). After 15 min on ice, 2.5 M sucrose [0.5x (vol/wt)] was added and samples were centrifuged at 800 x g for 5 min. The supernatant was collected as the cytosolic fraction, and the nuclear pellet was washed with buffer A. Following centrifugation, the nuclear pellet was resuspended in 5x (vol/wt) buffer B [10 mM HEPES-KOH pH 7.4, 0.42 M NaCl, 2.5% (vol/vol) glycerol, 1.5 mM MgCl, 0.5 mM EDTA, 0.5 mM EGTA, 1 mM DTT, 1x protease inhibitors], and incubated at 4°C while rotating at 60 rpm for 1 hour. Both the nuclear and cytosolic fractions were then centrifuged at 16,100 x g for 10 min at 4° C.

Sequential Biochemical Fractionation

Spinal cords from mice were dissected, weighed, and homogenized in 4 mL/g of high-salt buffer (HS buffer: 50 mM Tris pH 7.5, 750 mM NaCl, 5 mM EDTA, and protease inhibitor mixture), and then centrifuged at 45,000 x g for 30 min at 4° C. The pellets were extracted with HS containing 1% (wt/vol) Triton X-100 (TX fractions). Pellets were homogenized in 500 μL of HS buffer containing 1% (wt/vol) Triton X-100 and 1 M sucrose, and upon centrifugation, floating myelin was removed. Pellets were then extracted with 2 mL/g of urea buffer (7 M urea, 2 M thiourea, 4% (wt/vol) CHAPS, 30 mM Tris pH 8.5), followed by 2 mL/g of SDS loading buffer. Equivalent volumes of samples were separated on 10% Bis-Tris gels for immunoblotting with the indicated antibodies.

Total-body mouse γ-irradiation

To obtain a positive control for DNA damage accumulation in mouse spinal cords, Non-Tg mice (n = 3) were irradiated for 10 min at 10Gy of γ-rays. Animals were perfused within 2 hours after irradiation and processed for immunofluorescence staining using anti-γH2A.X and −53BP1 antibodies as previously described (Ahmed et al., 2017; Koch et al., 2016).

Cell viability assay

The viability of the neurons was tested using LIVE/DEAD Reduced Biohazard Viability/Cytotoxicity Kit (Molecular Probes). Neurons grown in 96 well plates were incubated with the kit solution mix for 15 min in complete darkness at room temperature. The cells were then rinsed with HBSS and fixed with freshly prepared 4% of glutaraldehyde for 15 min at room temperature before imaging by confocal microscopy.

Multi-electrode array (MEA)

Each well of a 12-well MEA plate from Axion Biosystems was coated with poly-D-lysine prior to cell seeding. Hippocampal neurons from Non-Tg and hg*FUS*^{R521H} mice embryos were plated at the same density (40,000 neurons per well), with duplicate wells for each embryo. Cells were fed every 3-4 days and measurements were taken before the medium was changed. Recordings were performed using a Maestro MEA system and AxIS software (Axion Biosystems), using a band-pass filter with 0.1 Hz and 5 kHz frequency cutoffs. Spike detection was performed using the neural Spikes analog setting (1200XGain, 200-5000Hz) and an adaptive threshold set to 5.5 times the standard deviation of the estimated noise on each electrode. Each plate first rested for 5 min in the Maestro, and then 5 min of data were recorded to calculate the spike rate per well. MEA analysis was performed using the Axion Biosystems Neural Metrics Tool, wherein electrodes that detected at least 5 spikes/minute were classified as active electrodes. Bursts were identified in the data recorded from each individual electrode using an adaptive threshold algorithm. Network bursts were identified for each well requiring a minimum of ten spikes with a maximum inter-spike of 100 ms. Only channels that exhibited bursting activity (more than 10 spikes in 5-min interval) were included in the analysis.

Compartmentalized microfluidic devices

The microfluidic devices were prepared as previously described (Niederst et al., 2015). Master molds were fabricated by photolithography, by the Bioengineer Department of the University of California, San Diego, Nano3 Cleanroom Facility. Devices were molded by soft lithography using Sylgard 182 (Ellsworth Adhesives, Germantown, WI) as previously described (Taylor et al., 2005). After cured, the cut devices where bath-sonicated in water, washed in 70% ethanol and dried under the hood before mounted onto glass coverslips. The devices were coated with 1X poly-D-lysine 2 hours at 37°C and washed sequentially in water and PBS1x before plating the cells.

Puromycin incorporation assay

Primary hippocampal neurons after 5 or 15 days of culture were incubated for 10 min with puromycin at 1μg/mL. After the incubation cells were washed with PBS1x and fixed with 4% of paraformaldehyde, permeabilized and stained with the anti-puromycin antibody

(1:100) for 1 hour at room temperature. Fluorescence was visualized using an Olympus confocal microscope and images were acquired using a 60X objective.

[^{35}S]-Methionine Labeling/Radioactivity assay

Primary hippocampal neurons were cultivated for 15 days. Medium in each well was exchanged to Met-/Cys- medium for 25 min before being supplemented with 1mCi/mL [35S]-Met media (for a final dosage of 0.1mCi/mL). [S35]-Met media containing 25μg/mL cycloheximide was used as negative control for radioactivity incorporation. After 2 hours of incubation with [35S]-Met at 0.1mCi/mL, incorporation was stopped with cycloheximide (25μg/mL) and cells collected immediately in ice-cold PBS1X. Cells were lysed with RIPA buffer (50mM Tris pH8.0, 50mM NaCl, 5mM EDTA, 1% NP-40, 0.5% sodium deoxycholate, 0.1% SDS) containing proteinase inhibitors and total cellular protein was co-precipitated with 100μg of BSA carrier protein using trichloroacetic acid. Samples were acetone washed, resuspended in 2% SDS-PBS1X, and transferred into scintillation fluid. Counts per minute (Cpms) averaged over 5-min windows were measured with a Beckman LS6000 SC scintillation counter (normalized to scintillation fluid only). Cpms were then normalized to total well protein concentration determined by densitometry from a silver-stained gel.

RNA extraction and qPCR

To isolate total RNA from cells or tissues, TRIzol (Invitrogen) and treatment with RQ1 DNase I (Promega) was used. Reverse transcription was performed using the SuperScript First Strand Kit (Invitrogen) according to manufacturer's instructions. qPCR was performed on 40ng of cDNA using the iQSYBR Green Supermix (Bio-Rad) with the iCycler iQ detection system according to manufacturer's instructions. Analysis was performed using the iQ5 optical system software (Bio-Rad; version 2.1). Expression values were expressed as a percentage of the average expression of control samples. All reactions were carried out in duplicate in three independent mice (per genotype) and *actin B* or *cyclophilin* and/or *rsp9* genes were also measured as standard genes across all experimental conditions. To enable determining human *FUS* RNA levels compared to mouse *Fus*, primer sequences common to both human and mouse were used to measure total *FUS* RNAs. In parallel, primer sequences specific to mouse *Fus* were designed to measure uniquely mouse *Fus* RNAs. Human *FUS* (compared to endogenous) is then obtained by subtracting mouse *Fus* from total *FUS* levels. Real-time quantitative RT-PCR (RT/qPCR) was performed to determine mRNA levels in spinal cord or hippocampal neurons from transgenic mice (see primer sequence in Table S2).

RNA-seq

Total RNA from spinal cords of 18-month-old m*Fus*$^{-/-}$/hg*FUS* (WT, R521C or R521H) and their Non-Tg control littermates were extracted with TRIzol (Invitrogen). RNA quality was measured using the Agilent Bioanalyzer system according to the manufacturer's recommendations and processed using the Illumina TruSeq Stranded mRNA Sample Preparation Kit according to manufacturer's protocol. A total of 14 cDNA libraries were simultaneously generated and sequenced using an Illumina HiSeq 2000 sequence, as previously described (Scekic-Zahirovic et al., 2016), before fastq files were obtained from Illumina demultiplexing "bcl2fastq". Then fastq files were aligned to a mouse reference genome (mm9, UCSC Genome Browser) using TopHat (Trapnell et al., 2009) and the transcript abundance for each annotated protein-coding gene as fragments per kilobase of transcript per million mapped reads (FPKM) were estimated by Cufflinks (Trapnell et al., 2010). Sequencing yielded, on average, 15 million non-redundant reads per sample. 13,468 genes, which expressed FPKM > = 1 were kept for downstream analyses. The spearman correlation among samples as well as Principle Component Analysis (PCA) was applied to those *12,922* genes (expressing consistently within the same category). The proportion of variance for dimension 1 (PC1) and dimension 2 (PC2) shown in Figure 5B was of 0.9 and 0.1, respectively. Cuffdiff (Trapnell et al., 2013), a part of the Cufflinks package, was used to detect the differentially expressed genes (DEGs). Unsupervised hierarchical clustering with complete method was applied on the heatmap showing DEGs between wild-type and mutant samples. Gene ontology of those DEGs was estimated by David and Ingenuity Pathway Analysis (IPA). All sequencing raw data were submitted to Gene Expression Omnibus (GEO) with accession number GSE120247.

RASL-seq

RASL-seq analysis of splicing switches was carried out as detailed (Scekic-Zahirovic et al., 2016; Sun et al., 2015; Zhou et al., 2012). A pool of oligonucleotides was designed to detect 5,859 alternative splicing events. One hundred fmol of RASL-seq oligos were annealed to 1 μg of total RNA isolated from mouse spinal cords. After ligation, 5 μl eluted ligated oligos was used for 16~20 cycles of PCR amplification, and the bar-coded PCR products were sequenced on HiSeq2000 with 24-30 samples in one lane. Sequencing data were decoded allowing no mismatch with each barcode, and target sequences were mapped with RASL-seq oligo pool sequences. Ratios of the counts of shorter to longer isoforms were calculated.

Splicing gels

Semiquantitative qRT-PCR (25-30 cycles) was used to assess splicing changes most affected upon FUS depletion identified by (Lagier-Tourenne et al., 2012). Isoform products were separated on 10% polyacrylamide gels and stained with SYBR gold (Invitrogen) and quantified with ImageJ software to record the intensity of the bands corresponding to different splicing isoforms.

Mouse behavioral tests

Grip strength

Grip strength was measured using a Grip Strength Meter (Columbus Instruments, Columbus, OH) on cohorts (n $\geq$ 15) made up of approximately the same number of males and females. Mice were allowed to grip a triangular bar only with hind limbs, followed by pulling the mice until they released; five force measurements were recorded in each separate trial.

Y maze test

Spontaneous alternation behavior, a measure of spatial working memory, exploratory behavior, and responsiveness to novelty (Lalonde, 2002), was tested using a Y maze with 34 × 8 × 14-cm arms. Each mouse was tested in a single 5-min trial in which arm choices and total numbers of arm entries were recorded. Spontaneous alternation, expressed as a percent, refers to the ratio of sets of three unique arm choices (i.e., visiting arm 3 then 1 then 2 in sequence) to the total number of arm entries. Because mice have the opportunity to do repeated entries into a single arm, there is a chance performance level of 22% (2/9) for spontaneous alternations (Holcomb et al., 1999; Pennanen et al., 2006). In our hands, healthy young C57BL6/J mice typically make 50%–70% spontaneous alternations in this test. A cohort of n = 15 animals (per genotype) was assessed.

Social interaction test

This test was originally developed to model in mice aspects of autism spectrum disorders in humans (Moy et al., 2004; Moy et al., 2007) and has been used widely by behavioral neuroscientists (Silverman et al., 2010). Individuals on the autism spectrum show aberrant reciprocal social interaction, including low levels of social approach and unusual modes of interaction. The social interaction apparatus is a rectangular, three chambered Plexiglas box, with each chamber measuring 20 cm x 40.5 cm x 22 cm (L x W x H). Dividing walls are clear with small semicircular openings (3.5 cm radius) allowing access into each chamber. The middle chamber is empty, and the two outer chambers contain small, round wire cages (Galaxy Cup, Spectrum Diversified Designs, Inc., Streetsboro, OH) during testing. The mice were habituated to the entire apparatus with the round wire cages removed for 5 min. To assess sociability, mice were returned to the middle chamber, this time with a stranger mouse (B6 of the same sex being tested, habituated to the wire cage) in one of the wire cages in an outer compartment and another identical wire cage in the opposite compartment. Time spent in the chamber with the stranger mouse and time spent in the chamber with the novel object was recorded for 5 min. For the social novelty preference test, mice were returned to the middle chamber, this time with the original mouse (familiar mouse) in its chamber and a new unfamiliar mouse (novel mouse) in the previously empty wire cage. Again, time spent in each chamber was recorded for 5 min. Young male B6 mice spend more time with the novel mouse in the sociability test (Moy et al., 2004), however we have found less preference for the novel mouse as compared to the now familiar mouse in the social novelty preference test (Jackson et al., 2015). A cohort of n = 15 animals (per genotype) was assessed.

Novel object recognition test

This test assays recognition memory while leaving the spatial location of the objects intact and is believed to involve the hippocampus, perirhinal cortex, and raphe nuclei (Lieben et al., 2006; Mumby et al., 2005; Winters et al., 2004). The basic principal is that animals explore novel environments and that with repeated exposure decreased exploration ensues (i.e., habituation). A subsequent object substitution (replacing a familiar object with a novel object) results in dishabituation of the previously habituated exploratory behavior (Ennaceur and Delacour, 1988). The resulting dishabituation is expressed as a preferential exploration of the novel object relative to familiar features in the environment. This dishabituation has generally been interpreted as an expression of the animal's recognition memory: the novel object is explored preferentially because it differs from what the animal remembers (Heyser and Chemero, 2012) and requires attention by the animals. Mice were individually habituated to a 51cm x 51cm x 39cm open field for 5 min and then tested with two identical objects placed in the field (either two 250 mL amber bottles or two clear plastic cylinders 6x6x16cm half filled with glass marbles). Each mouse was allowed to explore the objects for 5 min. After three such trials (each separated by 1 min in a holding cage), the mouse was tested in the object novelty recognition test in which a novel object replaced one of the familiar objects (for example, an amber bottle if the cylinders were initially used). All objects and the arena were thoroughly cleaned with 70% ethanol between trials to remove odors. Behavior was video recorded and then scored for contacts (touching with nose or nose pointing at object and within 0.5 cm of object). Habituation to the objects across the familiarization trials (decreased contacts) is an initial measure of learning and then renewed interest (increased contacts) in the new object indicated successful object memory. Recognition indexes were calculated using the following formula: # contacts during test/(# contacts in last familiarization trial + # contacts during test). Values greater than 0.5 indicate increased interest, whereas values less than 0.5 indicate decreased interest in the object during the test relative to the final familiarization trial. A cohort of n = 15 animals (per genotype) was assessed.

Cued and contextual fear conditioning

In this procedure, mice learn to associate a novel environment (context) and previously neutral stimuli (conditioned stimuli, a tone and light) with an aversive foot shock stimulus (Maren, 2001). It allows for the assessment of both hippocampus-dependent and amygdala-dependent learning processes in the same mouse (Kenney and Gould, 2008; Rudy et al., 2004). Testing then occurs in the absence of the aversive stimulus. Conditioned animals, when exposed to the conditioned stimuli, tend to refrain from all but respiratory movements by freezing. Freezing responses can be triggered by exposure to either the context in which the shock was received (context test) or the conditioned stimulus (CS+ test). Conditioning took place in Freeze Monitor chambers (Med Associates, Inc.) housed in sound proofed boxes. The conditioning chambers (26 × 26 × 17 cm) were made of Plexiglas with speakers and lights mounted on two opposite walls and shockable grid floors.

On day 1, mice were placed in the conditioning chamber for five minutes in order to habituate them to the apparatus. On day 2, the mice were exposed to the context and conditioned stimulus (30 s, 3000 Hz, 80 dB sound + white light) in association with foot shock (0.60 mA, 2 s, scrambled current). Specifically, the mice received 3 shock exposures in their 6 min test, each in the last 2 s of a 30 s tone/light exposure. On day 3, contextual conditioning (as determined by freezing behavior) was measured in a 5-min test in the chamber where the mice were trained (context test). On the following day, the mice were tested for cued conditioning (CS+ test). The mice were placed in a novel context for 3 min, after which they were exposed to the conditioned stimuli (light + tone) for 3 min. For this test, the chamber was disguised with new walls (white opaque plastic creating a circular compartment in contrast to a clear plastic square compartment) and a new floor (white opaque plastic in contrast to metal grid). Freezing behavior (i.e., the absence of all voluntary movements except breathing) was measured in all of the sessions by real-time digital video recordings calibrated to distinguish between subtle movements, such as whisker twitches, tail flicks, and freezing behavior. Freezing behavior in the context and cued tests (relative to the same context prior to shock and an altered context prior to tone, respectively) is indicative of the formation of an association between the particular stimulus (either the environment or the tone) and the shock; i.e., that learning had occurred. A cohort of n = 15 animals (per genotype) was assessed.

QUANTIFICATION AND STATISTICAL ANALYSIS

The number of independent repeats (n), the statistical test used for comparison and the statistical significance (p values) are specified for each figure panel in the representative figure legend. Data are presented as mean ± SEM. Data were analyzed and graphs were generated using GraphPad Analysis software.

DATA AND SOFTWARE AVAILABILITY

RNA sequencing data is submitted to Gene Expression Omnibus (GEO) with accession number GSE120247.

Neuron, Volume *100*

Supplemental Information

ALS/FTD-Linked Mutation in FUS Suppresses Intra-axonal Protein Synthesis and Drives Disease Without Nuclear Loss-of-Function of FUS

Jone López-Erauskin, Takahiro Tadokoro, Michael W. Baughn, Brian Myers, Melissa McAlonis-Downes, Carlos Chillon-Marinas, Joshua N. Asiaban, Jonathan Artates, Anh T. Bui, Anne P. Vetto, Sandra K. Lee, Ai Vy Le, Ying Sun, Mélanie Jambeau, Jihane Boubaker, Deborah Swing, Jinsong Qiu, Geoffrey G. Hicks, Zhengyu Ouyang, Xiang-Dong Fu, Lino Tessarollo, Shuo-Chien Ling, Philippe A. Parone, Christopher E. Shaw, Martin Marsala, Clotilde Lagier-Tourenne, Don W. Cleveland, and Sandrine Da Cruz

A

Transgene	Strain	# founder lines	# established lines
hgTLS^{WT}	C57BL/6	19	4
hgTLS^{R521C}	C57BL/6	2	1
hgTLS^{R521H}	C57BL/6	5	3

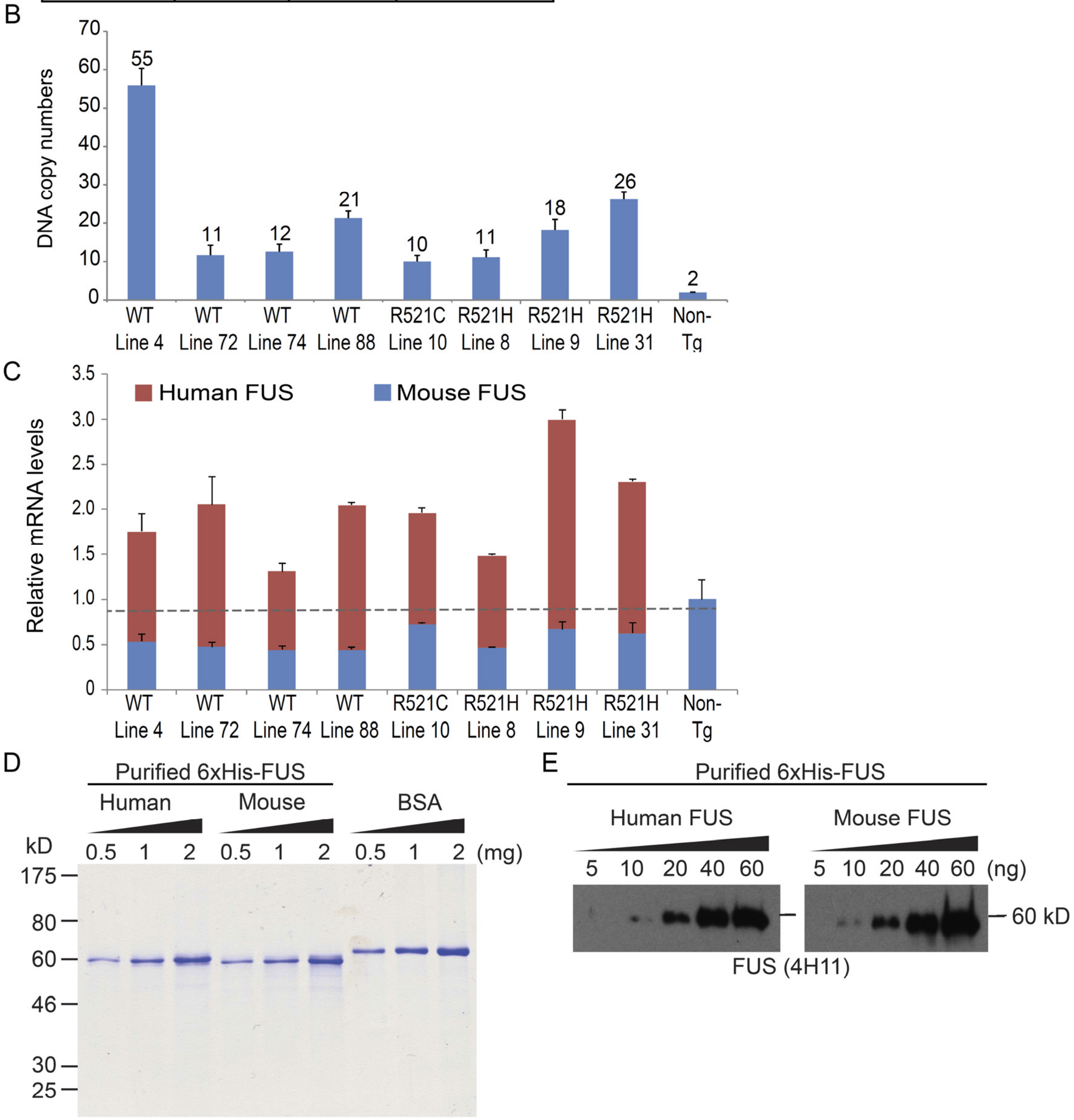

Figure S1: Expression levels of the human *FUS* transgene mimics that of endogenous protein with an auto-regulation mechanism. (Related to Figure 1) (A) Summary of the hg*FUS* transgenic lines per genotype that were generated. (B) DNA copy numbers found in tails of Non-Tg, hg*FUS*^{WT}, hg*FUS*^{R521C} and hg*FUS*^{R521H} mice by qPCR (n=3 mice per line). The bar graph represents mean ± SEM. (C) Expression levels of total (mouse and human) *FUS* RNAs in cortex from 2-month-old Non-Tg, hg*FUS*^{WT}, hg*FUS*^{R521C} and hg*FUS*^{R521H} mice measured by qRT-PCR, normalized to the level of endogenous *Fus* RNA (n=3 mice/line). The bar graph represents mean ± SEM. (D) Purified his-tagged full length human and mouse FUS recombinant proteins analyzed by Coomassie staining of a SDS-polyacrylamide gel. (E) Immunoblotting using FUS antibody (4H11) to determine its affinity for human and mouse FUS proteins. The right and left immunoblot panels with recombinant mouse and human FUS, respectively belong to the same original blot, which was digitally cut as non-relevant samples were loaded in between.

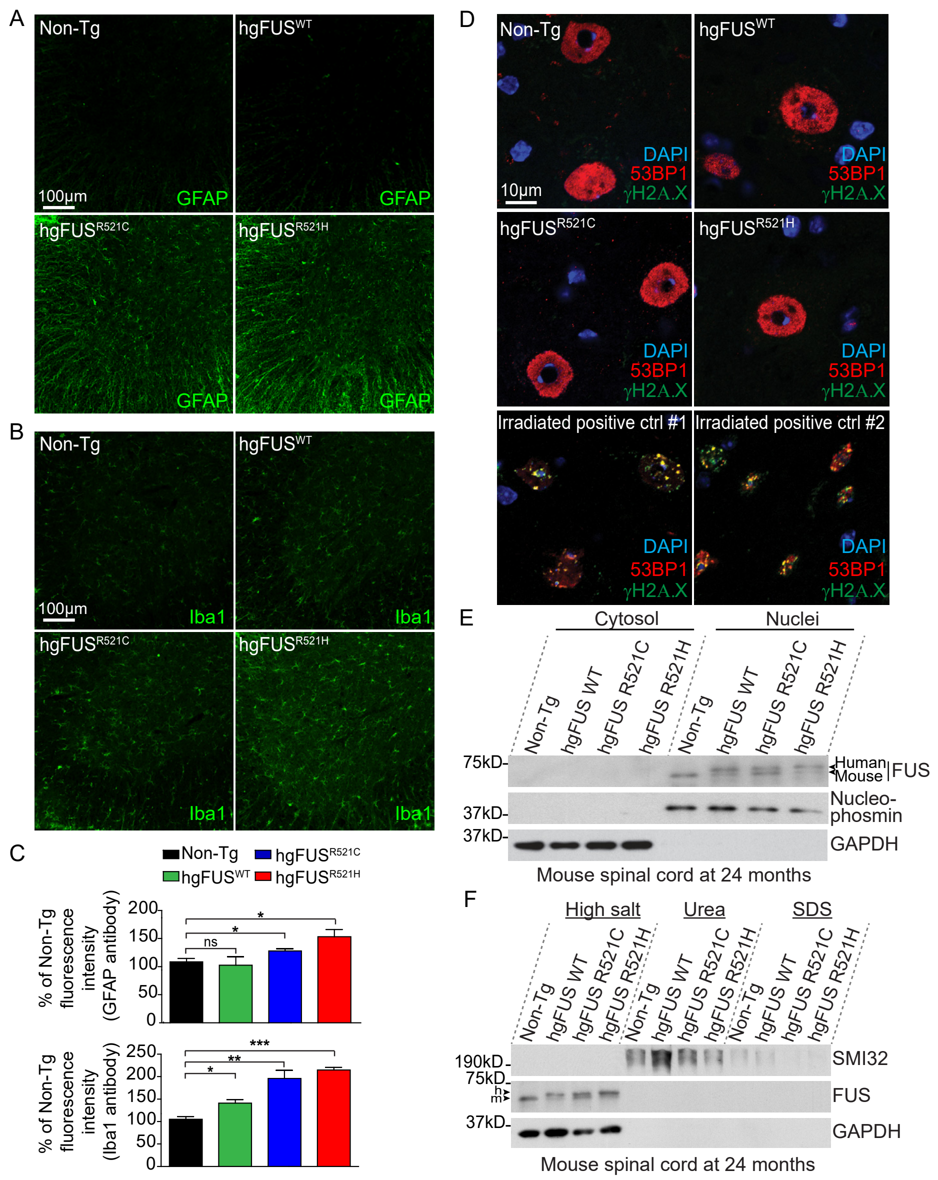

Figure S2: Age-dependent progressive lower motor neuron disease without DNA damage accumulation or cytoplasmic aggregation of FUS in mice expressing ALS-linked mutants of human FUS. (Related to Figure 2) (A-B) Lumbar spinal cord sections from 24-month-old Non-Tg, hg*FUS*^{WT}, hg*FUS*^{R521C} and hg*FUS*^{R521H} mice processed for immunofluorescence using an antibody detecting activated (A) astrocytes (GFAP) or (B) microglia (Iba1). Scale bar, 100 μm. (C) Quantification of the relative fluorescence intensity of the GFAP and Iba1 staining of lumbar spinal cord sections from 24-month-old Non-Tg, hg*FUS*^{WT}, hg*FUS*^{R521C} and hg*FUS*^{R521H} mice. The bar graph represents mean ± SEM from n=3 animals per genotype. * $p<0.05$, ** $p<0.01$, *** $p<0.001$, two-sided unpaired Student's t-test. (D) Lumbar spinal cord sections from 24-month-old Non-Tg, hg*FUS*^{WT}, hg*FUS*^{R521C} and hg*FUS*^{R521H} mice processed for immunofluorescence using 53BP1 (red) and γH2A.X (green) antibodies staining for DNA damage as revealed by co-localized aggregation in the positive control irradiated mice. DNA is stained with DAPI. Scale bar, 10 μm. (E-F) Immunoblot demonstrating (E) lack of cytoplasmic redistribution or (F) major aggregation of FUS using an antibody recognizing both species with similar affinity. (E) Enrichment for nuclear and cytosolic proteins was confirmed using Nucleophosmin and GAPDH antibodies, respectively. (F) SMI32 (neurofilament) and GAPDH antibodies were used as markers for proteins solubilized in the presence of an urea- or high-salt-containing buffer, respectively. Each lane represents an independent 24-month-old mouse spinal cord.

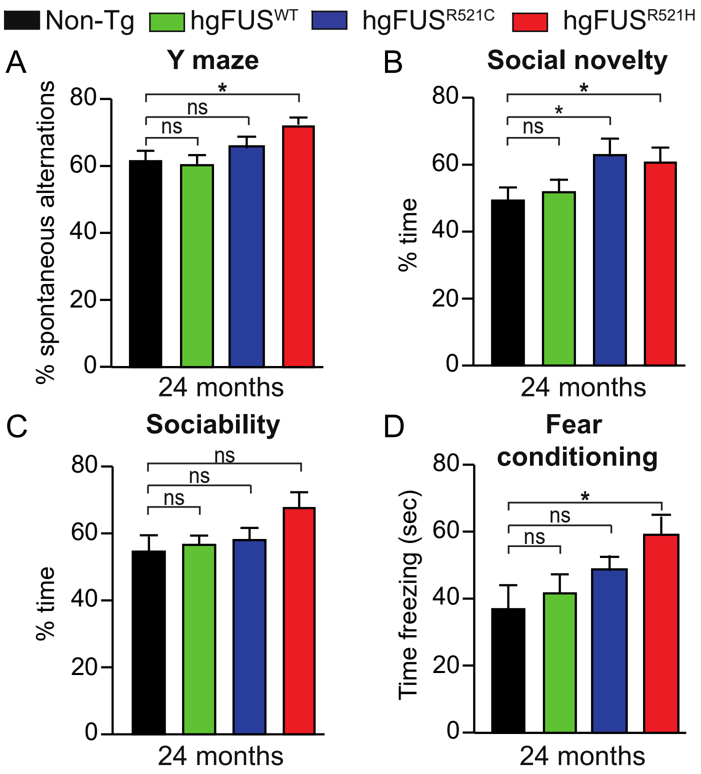

Figure S3: Age-dependent cognitive impairments in mice expressing ALS-linked mutants of human FUS (Related to Figure 3) (A) Spatial memory (Y maze), (B) social novelty, (C) sociability and (D) anxiety (fear conditioning) were measured in 24-month-old Non-Tg, hgFUS^{WT}, hgFUS^{R521C} and hgFUS^{R521H} mice. Data are represented as mean ± SEM from n=15 animals (both genders) per genotype. * $p<0.05$, ns: non-significant using one-way ANOVA.

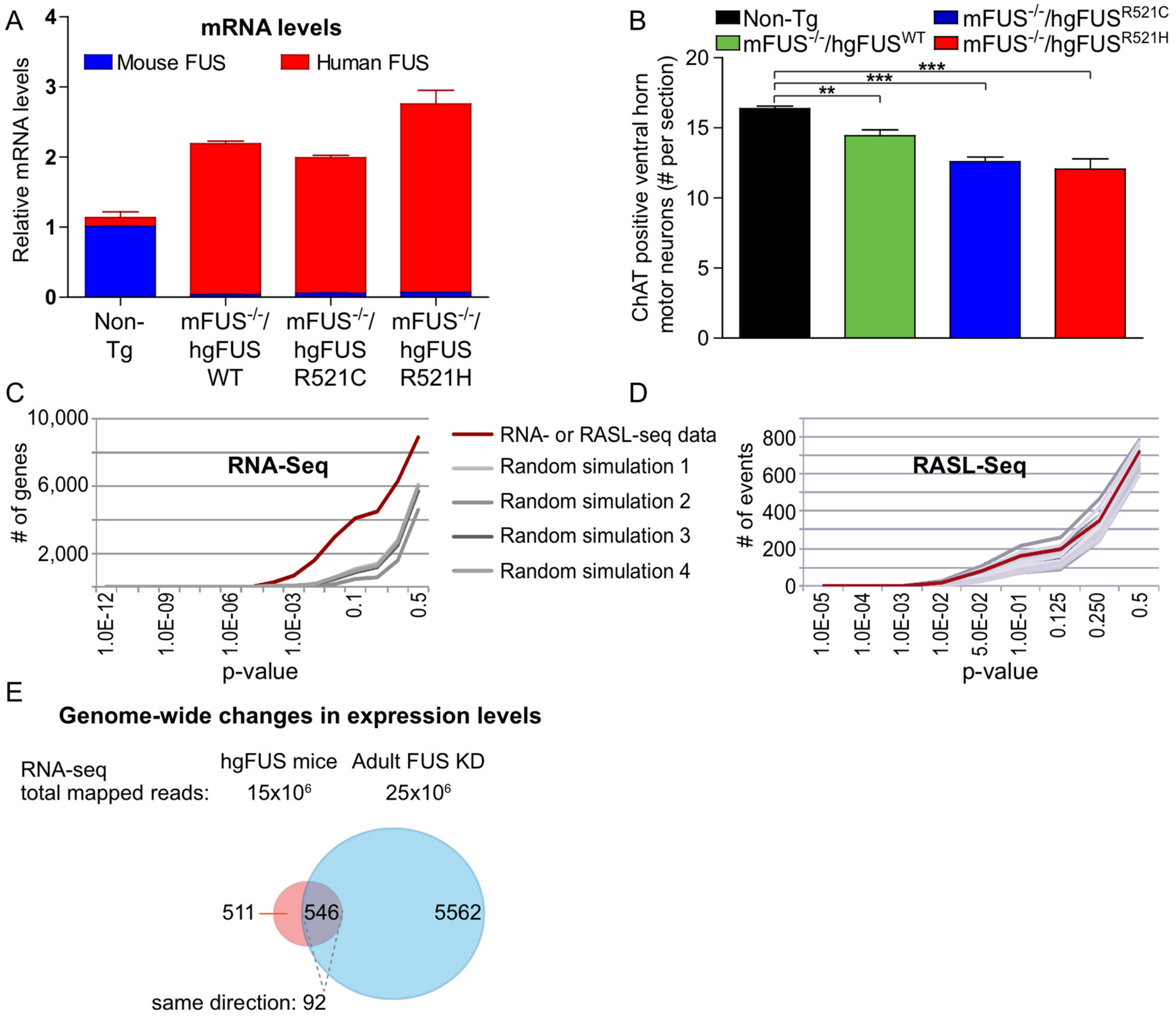

Figure S4: Mutant-dependent RNA signature associated with motor deficits by toxic gain-, but not loss-of-function of ALS-linked mutants of human FUS. (Related to Figures 4-5) (A) Expression levels of total (mouse and human) *FUS* RNAs in spinal cord of Non-Tg, m*Fus*$^{-/-}$/hg*FUS*^{WT}, m*Fus*$^{-/-}$/hg*FUS*^{R521C} and m*Fus*$^{-/-}$/hg*FUS*^{R521H} mice measured by qRT-PCR, normalized to the level of endogenous *FUS* RNA (n=3 mice per line). (B) Quantification of the total number of ChAT positive motor neurons in lumbar spinal cords of 18-month-old Non-Tg, m*Fus*$^{-/-}$/hg*FUS*^{WT}, m*Fus*$^{-/-}$/hg*FUS*^{R521C} and m*Fus*$^{-/-}$/hg*FUS*^{R521H} mice. The bar graph represents mean ± SEM from n≥3 animals per genotype. ** p<0.01 and *** p<0.001, two-sided unpaired Student's t-test. (C) RNA-seq analysis of all the genes that were differentially expressed in spinal cords from mutants m*Fus*$^{-/-}$/hg*FUS*^{R521C} and m*Fus*$^{-/-}$/hg*FUS*^{R521H} mice (red curve) compared with the dataset where the genotypes were randomly shuffled. (D) RASL-seq analysis of all the splicing events that were identified in spinal cords from mutants m*Fus*$^{-/-}$/hg*FUS*^{R521C} and m*Fus*$^{-/-}$/hg*FUS*^{R521H} mice (red curve) compared with the dataset where the genotypes were randomly shuffled. No significant splicing events were identified that are distinct in the mutant mice compared to randomized splicing events. (E) Venn diagrams showing the overlap of significantly differentially expressed genes in spinal cords from mutant m*Fus*$^{-/-}$/hg*FUS*^{R521C} and m*Fus*$^{-/-}$/hg*FUS*^{R521H} animals compared to adult mouse spinal cords depleted of FUS upon antisense oligonucleotide treatment identified by (Kapeli et al., 2016).

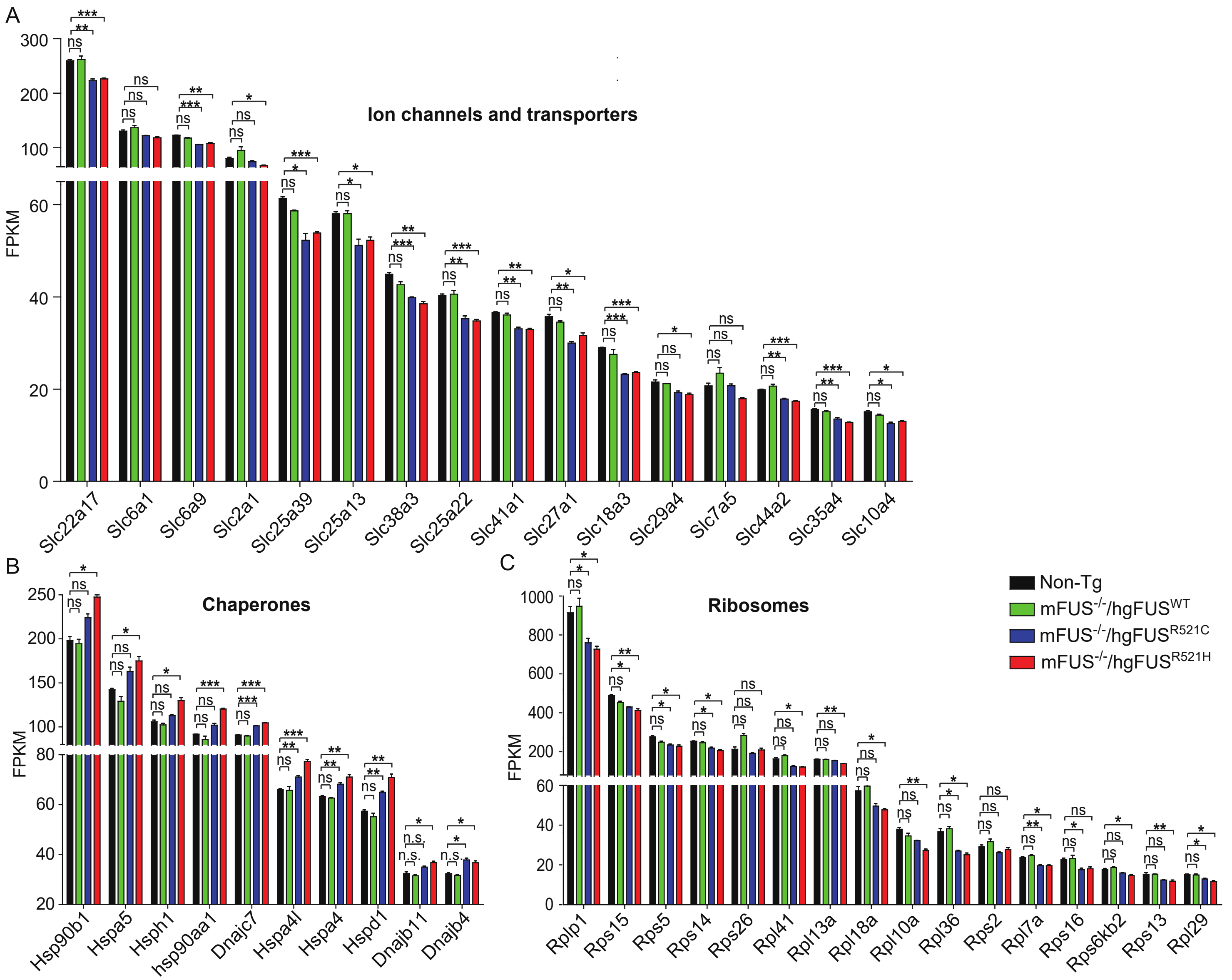

Figure S5: Mutant-dependent RNA signature associated with motor deficits caused by toxic gain-of-function of ALS-linked mutants of human FUS. (Related to Figures 5) (A) Example of expression of ion channels and transporters essential for synaptic function found to be down-regulated by RNA-seq analysis [FPKM (fragments per kilobase of transcript per million mapped reads)] of spinal cord of 18-month-old mutant m*Fus*$^{-/-}$/hg*FUS*^{R521C} and m*Fus*$^{-/-}$/hg*FUS*^{R521H} mice compared to age-matched Non-Tg. Data are represented as mean ± SEM from n≥3 animals per genotype. * p<0.05, ** p<0.01 and *** p<0.001, two-sided unpaired Student's t-test. (B) Example of the chaperone encoded genes found to be up-regulated by RNA-seq analysis [FPKM values] of spinal cord of 18-month-old mutant m*Fus*$^{-/-}$/hg*FUS*^{R521C} and m*Fus*$^{-/-}$/hg*FUS*^{R521H} mice compared to age-matched Non-Tg and m*Fus*$^{-/-}$/hg*FUS*^{WT} animals. Data are represented as mean ± SEM from n≥3 animals per genotype. * p<0.05 and ** p<0.01, two-sided unpaired Student's t-test. (C) Example of the ribosome genes found to be down-regulated by RNA-seq analysis [FPKM values] of spinal cord of 18-month-old mutant m*Fus*$^{-/-}$/hg*FUS*^{R521C} and m*Fus*$^{-/-}$/hg*FUS*^{R521H} mice compared to age-matched Non-Tg and m*Fus*$^{-/-}$/hg*FUS*^{WT} animals. Data are represented as mean ± SEM from n≥3 animals per genotype. * p<0.05 and ** p<0.01, two-sided unpaired Student's t-test.

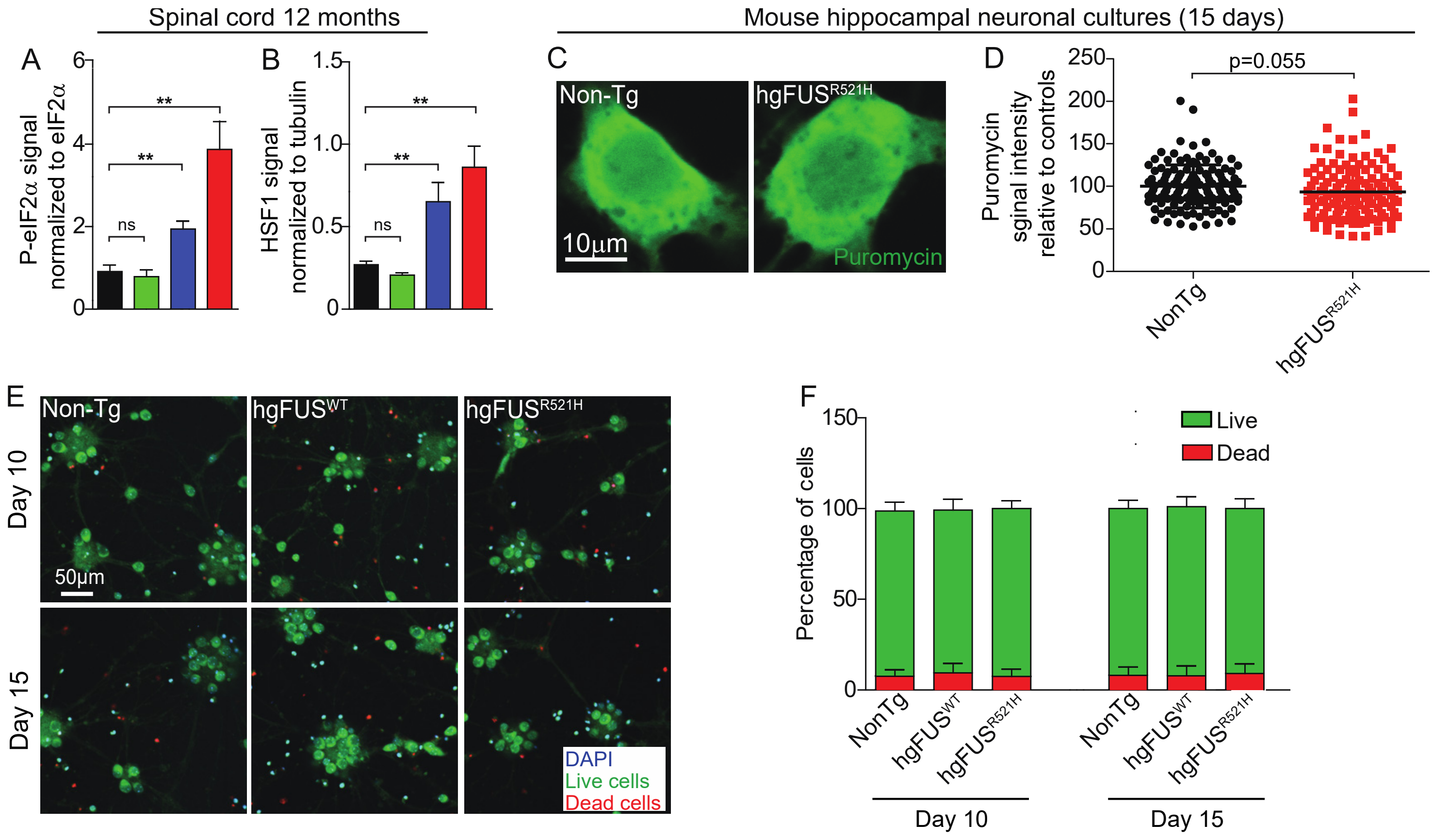

Figure S6: Mutation in FUS causes stress-mediated reduction in axonal protein synthesis (related to Figure 6) (A-B) Quantification of immunoblotting using P-eIF2α and HSF1 antibodies in mouse spinal cords (at 12 months of age), shown in Figure 6A. Data are represented as mean ± SEM from three independent experiments. (C-D) Additional representative images of primary hippocampal cell bodies (cultured for 15 days) stained with antibody against puromycin and quantification of the relative fluorescence intensity of puromycin per cell bodies. Each dot represents the fluorescence intensity per cell body in each genotype. N=130. (E-F) Representative micrograph and quantification of the viability of hippocampal neurons after 10 or 15 days in culture. Live cells are in green, dead cells in red and nuclei in blue.

Table S1: List of genes with altered expression in spinal cords of mutant FUS mice (related to Figure 5C). See individually uploaded Supplemental Table 1 file.

Table S2: Related to Figures 1C, 5F, 5G, 5H, 6B, S1C and S4A. qPCR primers sequences

Gene Name	Forward 5'-3'	Reverse 5'-3'
Mouse FUS	GGCCAGTCAGCTGACACTTCAG	TGCTGCCATAGCCTCCAGTGGAA
Human/Mouse FUS	TCCCAGCAGAGCAGTCAGCCCTA	CCTGGGGAGTTGACTGAGTTCC
Human/Mouse FUS (genomic DNA)	TGGGTTCATGACTTAGGTGTCATTG	CATGTCCCAGGGAACAGACCAG
Slc25a15	CAGGACTCGTCAGGACCTTC	TGCTTCCAATTGGTTCATCA
Slc2a1	GTCCTGCTGCTATTGCTGTG	GAGCACCGTGAAGATGATGA
Scl19a3	CAGGCCTTCAGAGGCATTCT	GGTAGTCGGTGAGGACAAACAC
Slc17a7	AGCATCTTGATGGGCATTTC	CGAAGCAAAGACCCCATAGA
Slc1a3	AGAACCCCTGGGTTTTCATT	GGACAAGTGCTCGACAATCC
Slc39a2	GTCATCACCACAGGGTCCTC	GAAGCAGCATCACGAGAAGA
Slc41a1	ACATGACTGCTGCACTGCTC	ATGGGATGGAGAAGTTGTCG
Slc22a17	CCCACCAGACTTCAACCACT	GCAAAGCCCAAGATGAAGAG
Slc44a2	CGGATCAGAATTGTGCAAGA	CATGCCATAGACGCTGAAGA
Slc10a4	GGCAGTCACCCTGACTCTCT	AGAACCACCAGGGTCACAAG
Dnajc7	GATCCTGGGAGTGGACAAGA	TCCTTCTGAACTTCGGCACT
Hspa5	ATAAACCCCGATGAGGCTGT	GGGGACAAACATCAAGCAGT
Hsp90b1	GGATGCAGGGACAGAAGAAG	CTCCACACGGGATTCATAGC
Hspa4	GCAGAACAAACAGAGCCTGA	TGTTTTGGTTCCTCCTTTGG
Rpl41	AGGATGGCTACCATCACCTG	TCTTGGCAGCATACTCCTGA
Rps6kb2	AAGAGGGCTTCTCCTTCCAG	AACCCCTCAAAGGGAGAGAA
Rpl18a	CGCGAAAGACAACACTTCCT	GGTACAGTGGTGGTGTGTGG
Rps26	GGATCCTCTTTCGAGTCTTGG	TTCTTGATGGCCTTATCCTTG
Rps15	GAGAAGCCTGAGGTGGTGAA	CAGGTAGTGGCCGATCATCT
ATF4	GAAACCTCATGGGTTCTCCA	TCTCCAACATCCAATCTGTCC
Actin B	TTGCTGACAGGATGCAGAAG	ACATCTGCTGGAAGGTGGAC
Rps9	GACCAGGAGCTAAAGTTGATTGGA	GCGTCAACAGCTCCCGGGC
Cyclophilin	CAGACGCCACTGTCGCTT T	TGTCTTTGGAACTTTGTCTGCAA
